# Synthesis of a Ribose‐Incorporating Medium Ring Scaffold via a Challenging Ring‐Closing Metathesis Reaction

**DOI:** 10.1002/ejoc.201600756

**Published:** 2016-08-15

**Authors:** Stuart S. Rankin, John J. Caldwell, Nora B. Cronin, Rob L. M. van Montfort, Ian Collins

**Affiliations:** ^1^ Cancer Research UK Cancer Therapeutics UnitThe Institute of Cancer ResearchSM2 5NGLondonUnited Kingdom; ^2^ Division of Structural BiologyThe Institute of Cancer ResearchSW7 3RPLondonUnited Kingdom

**Keywords:** Medium‐ring compounds, Fused‐ring systems, Carbohydrates, Metathesis, Cyclization

## Abstract

A practical synthesis of a novel oxabicyclo[6.2.1]undecenetriol useful as a medicinal chemistry scaffold has been developed starting from l‐ribose. The sequence involves an oxidation/Grignard addition sequence and a challenging ring‐closing metathesis (RCM) reaction as the ring forming step. Exploration of the RCM substrate protecting groups revealed the key factor for successful nine‐membered medium ring formation to be conformational bias of the reacting alkenes of the RCM substrate by very bulky silyl ether protecting groups. The synthesis also allowed access to an epimeric triol and saturated and unsaturated variants of the nine‐membered ring. The medium ring conformation of the oxabicyclo[6.2.1]undecenetriol was determined by X‐ray crystallography and correlated to the solution state conformation by NMR experiments.

## Introduction

**Scheme 1:**
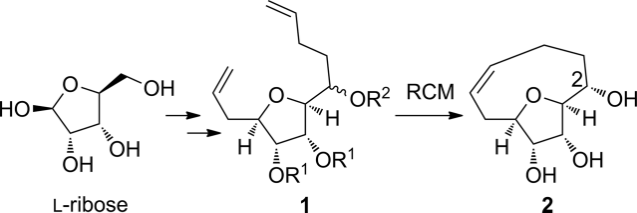
Overview of the synthesis of triol **2**.

Oxygen‐containing medium rings (defined as rings of 8–11 atoms) are found in bioactive natural products such as the cladiellanes,^1^ *Laurencia* medium ring ethers,^2^ and polycyclic ethers, e.g. brevetoxin‐A and ciguatoxin.^3^ The synthesis of medium rings is challenging due to both enthalpic and entropic penalties involved in their formation,^4^ but they can offer unique conformations and novel scaffolds for biological activity.^5^, ^6^, ^7^ We required a synthesis of an oxabicyclo[6.2.1]undecenetriol (**2**, Scheme 1) as a building block to investigate constrained nucleotide mimics. Herein we describe the development of a practical synthetic route to the novel triol **2**, as well as the *C*‐2 epimer and a saturated analogue, and elucidation of the conformation of the fused medium ring.

**Scheme 2:**
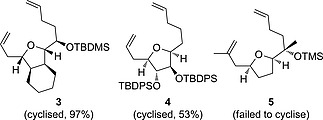
Reported RCM cyclisation of related bis‐alkenes.

The key ring forming reaction in the envisaged synthesis of triol **2** (Scheme 1) was a ring‐closing metathesis (RCM) reaction. RCM reactions have found extensive use in the synthesis of natural products.^8^, ^9^, ^10^, ^11^ This approach has been used with varying degrees of success on a broad range of substrates to overcome the challenges of medium ring and macrocycle formation.^12^, ^13^, ^14^ In particular, substrates related to the bis‐alkene **1**, such as a cyclohexane‐fused precursor (**3**)^15^ and a tetrahydrofuran diol with an *anti*‐configuration (**4**)^16^ have been reported to cyclise in good yields (Scheme 2). However, a substrate with no 3,4‐substitution of the tetrahydrofuran (**5**)^17^ failed to cyclise, highlighting how a nine‐membered ring closure can be very sensitive to changes in substrate structure. A key challenge in forming products such as **2** is overcoming the conformational flexibility of the precursor alkenyl chains, which is typically achieved by the introduction of conformational modifiers such as *gem*‐dimethyl substitution that favour ring formation.^18^ Another challenge is the potential for unfavourable chelation between the RCM catalyst and substrate oxygen atoms which stalls the reaction. This can often be overcome by the use of titanium isopropoxide as an additive.^18^

## Results and Discussion

**Scheme 3:**
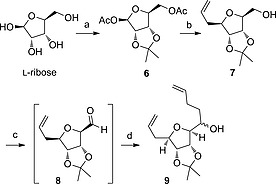
a) 1) H_2_SO_4_, acetone, 83 %. 2) Ac_2_O, pyridine, 75 %. b) 1) allylTMS, ZnBr_2_, MeNO_2_. 2) NaOMe, MeOH, 63 % over 2 steps. c) 1) See Table 1. 2) 3‐buten‐1‐ylmagnesium bromide, THF, yield over 2 steps in Table 1.

Synthesis began from the unnatural sugar, l‐ribose, which was fully protected as the acetonide and diacetate **6** using literature procedures (Scheme 3).^19^ Stereoselective allylation at the anomeric *C*‐1 was achieved using conditions reported for protected d‐ribose,^20^ giving an approximately 5:1 ratio of diastereoisomers as determined by ^1^H NMR spectroscopy. Hydrolysis of the acetyl ester gave alcohol **7**,^21^ which was purified as the major diastereoisomer in 50 % yield over the two steps. Alcohol **7** was oxidised to the aldehyde **8** to allow addition of 3‐buten‐1‐ylmagnesium bromide to install the second alkene for the RCM. The Grignard addition showed some stereoselectivity, giving alcohol **9** as a 3:2 mixture of epimers at the newly formed stereocentre as determined by ^1^H NMR spectroscopy. The epimers could not be separated at this stage and the major diastereoisomer was not determined.

The aldehyde **8** appeared unstable upon attempted purification, generating a complex mixture of products, and therefore the oxidation and Grignard addition were carried out as a tandem procedure with minimal handling of the aldehyde. Significant optimisation of the oxidation step was required to achieve a satisfactory yield for the two step process (Table 1). Initially Swern conditions gave the best overall yield from the oxidation/Grignard addition sequence. Changing the solvents used in the aqueous extraction of the intermediate aldehyde (from CH_2_Cl_2_ and saturated aqueous sodium hydrogen carbonate solution to ethyl acetate and water) improved the overall yield from 17 % to 29 %. By quenching the Swern reaction at –78 °C with addition of water and allowing the reaction to warm slowly before extracting the aldehyde, the two‐step yield was increased to 47 %. These optimised conditions were successfully scaled to 3 g of starting material **7** and gave a comparable yield of 53 %.

**Table 1:** Reaction conditions for formation of alcohol **9**

Oxidation	Aqueous addition	Isolated
reagents^a^	Temp. /°C^b^	yield of **9** /%^c^
A	room temp.	0
B	r.t.	7
C	r.t.^d^	17
C	r.t.^e^	29
C	< 0^e^	42
C	–78^e^	47

a A = TPAP, NMO, CH_2_Cl_2_, MS 4 Å, room temp.; B = Dess–Martin periodinane, CH_2_Cl_2_, r.t.; C = (COCl)_2_, DMSO, NEt_3_, CH_2_Cl_2_.

b Temperature of addition of NaHCO_3_ (aq.) or water to oxidation reaction.

c Yield over 2 steps from **7**.

d Extraction of intermediate **8** using CH_2_Cl_2_/NaHCO_3_ (aq.).

e Extraction of intermediate **8** using EtOAC/water.

**Scheme 4:**
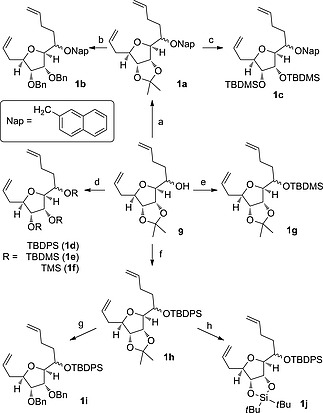
a) NapBr, NaH, *t*Bu_4_NI, DMF, 85 %. b) 1) HCl, MeOH, 54 %. 2) BnBr, NaH, DMF, 76 %. c) 1) (CH_2_SH)_2_, BF_3_ **·**OEt_2_, CH_2_Cl_2_, 55 %. 2) TBDMSOTf, 2,6‐lutidine, CH_2_Cl_2_, 89 %. d) 1) 9:1 TFA/water, 86 %. 2) ROTf, 2,6‐lutidine, CH_2_Cl_2_, 71–88 %. e) TBDMSOTf, DIPEA, CH_2_Cl_2_, quant. f) TBDPSOTf, 2,6‐lutidine, CH_2_Cl_2_ 80 %. g) 1) (CH_2_SH)_2_, PTSA, CH_3_Cl, 60 °C, 64 %. 2) BnBr, NaH, DMF, 65 %. h) 1) (CH_2_SH)_2_, PTSA, CH_3_Cl, 60 °C, 64 %. 2) (*t*Bu)_2_Si(OTf)_2_, 2,6‐lutidine, CH_2_Cl_2_, 68 %.

Several RCM precursor compounds were synthesised from the alcohol **9** with various combinations of protecting groups (Scheme 4), chosen to explore the effects of substituent size, conformational restriction and coordinating ability of the protected oxygen atoms on the success of the cyclisation. Protection of alcohol **9** as a benzyl, naphthalen‐2‐ylmethyl or silyl ether was followed by optional removal of the acetonide and re‐protection of the diol as benzyl or silyl ethers.

**Scheme 5:**
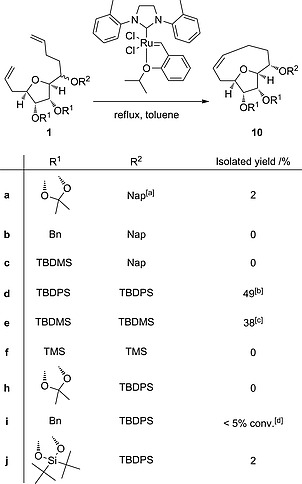
a) Nap = naphthalene‐2‐ylmethyl. b) Product was a 70:30 mixture of diastereoisomers at *C*‐2. c) Yield of triol **2** after deprotection. d) Conversion estimated by HPLC.

The medium ring forming RCM reaction was initially attempted on the TBDMS‐ and acetonide‐protected bis‐alkene **1g** using the conditions reported in the successful precedent to make **3** from our laboratory (Grubbs II catalyst in dichloromethane at room temperature or 40 °C),^15^ but no formation of the desired medium ring product was observed. Screening of alternative conditions was conducted using the naphthalene‐2‐ylmethyl‐ and acetonide‐protected substrate **1a** which allowed easier reaction monitoring by HPLC due to the presence of a strong UV chromophore. Four catalysts (Grubbs I and II, Hoveyda–Grubbs I and Stewart–Grubbs)^22^ and two solvents (dichloromethane and toluene) were tested at reflux and only one set of conditions displayed formation of the desired product: Stewart–Grubbs catalyst in toluene gave the medium ring product **10a** in 2 % yield (Scheme 5). Several by‐products were observed in the reaction and we attempted to limit these by reducing the concentration of the reaction (from 0.003 m to 0.0015 m or 0.0003 m) but at the lower concentrations no significant conversion was observed after five days. Inclusion of additives, titanium isopropoxide (to prevent chelation) or 1,4‐benzoquinone (to prevent isomerisation of the double bonds by ruthenium hydride species),^23^ did not improve the reaction profile.

The variously protected RCM precursors **1a**–**f** and **1h**–**j** were submitted to the most productive RCM reaction conditions (Scheme 5). The results allow some conclusions to be drawn about the key factors of successful medium ring formation. Firstly, prevention of chelation by global silyl ether protection was not in itself sufficient to allow medium ring formation as the tri‐TMS‐protected substrate **1f** did not cyclise. However, the bulkier tri‐TBDMS‐ and TBDMS‐protected substrates **1d** and **1e** did cyclise, suggesting a role for the larger size of these substituents. Substrates with larger protecting groups that prevented chelation at only one or two of the three pendant ether oxygen atoms (**1c**, **1h** and **1i**) were also unreactive. Another conclusion drawn is that the introduction of conformational bias to these substrates is beneficial to medium ring formation. Two of the three substrates **1a**, **1h**, and **1j** with a fused ring protecting group on the tetrahydrofuran diol gave a very small yield of medium ring product which was an improvement on the lack of significant medium ring product detected in the RCM of substrates with no ring fusion and less bulky protecting groups (**1b**, **1c**, **1i**). However, by far the most successful medium ring formation was observed for the tri‐TBDMS‐ and TBDPS‐protected substrates **1d** and **1e**. We speculate that the improved yield in these reactions is due to steric clash between the adjacent very bulky silyl ethers of the substrate, thereby restricting the conformational space available to the alkene arms, which are consequently more frequently close together and reducing the energy barrier for the initial ruthenium alkylidene and the second alkene to come into sufficient proximity for intramolecular reaction.

In the case of the tri‐TBDPS‐protected medium ring product **10d**, both diastereoisomers of the substrate **1d** cyclised, to give a 70:30 mixture of *C*‐2 diastereoisomers of the product. Every other isolated medium ring product was a single diastereoisomer, subsequently identified as the *C*‐2 (*S*) configuration, see below, with no evidence for formation of the other medium ring diastereoisomer. This suggests that the *C*‐2 (*S*)‐diastereoisomer of **1** more readily adopts a suitable conformation for successful medium ring formation than the (*R*)‐diastereoisomer. Consistent with this hypothesis, Cusson et al. recently reported the formation of a nine‐membered lactone by RCM reaction which was highly dependent on the configuration of one of three of the stereocentres in the substrate.^24^ Other examples of diastereoselective RCM reactions have been reported for five‐ and six‐membered ring formations.^25^, ^26^ Interestingly, there appear to be subtle differences between the tri‐TBDPS‐protected and tri‐TBDMS‐protected substrates that allows medium ring formation of both epimers for the former, possibly simply due to increased protecting group size.

We screened catalysts and reaction temperatures for the RCM using the optimal tri‐TBDPS‐protected substrate **1d**. All three catalysts tested were similarly effective at cyclisation at reflux in toluene (Table 2) and significant product formation was observed at reflux in dichloromethane for the Hoveyda–Grubbs II and Stewart–Grubbs catalysts. Some product formation was also observed at room temperature in both solvents using Stewart–Grubbs catalyst. The nature of the protecting groups therefore appears to be the most important factor for successful RCM on this substrate. However, the need for high temperatures and a less‐hindered catalyst revealed this medium ring to be particularly challenging to form by RCM.

**Table 2:** RCM conditions for conversion of **1d** to give **10d**

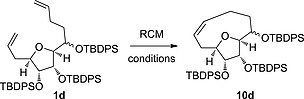					
Temperature /°C	Solvent	Catalyst^a^	Conversion of substrate **1d** to		
			product **10d** by HPLC /%^b^		
			1 h	18 h	48 h
Room temp.	CH_2_Cl_2_	SG	1.0	14	19
Room temp.	toluene	SG	4.8	30	38
40	CH_2_Cl_2_	HGII	32	58	66
40	CH_2_Cl_2_	SG	11	39	44
110	toluene	GII	88	92	94
110	toluene	HGII	80	80	83
110	toluene	SG	92	95	96

a SG = Stewart Grubbs; HG = Hoveyda–Grubbs II; GII = Grubbs II.

b Calculated from ratio of UV AUCs of **1d** and **10d** by HPLC.

**Scheme 6:**
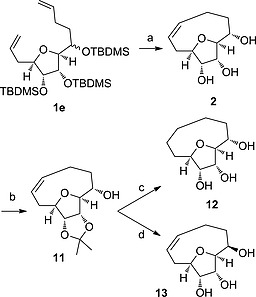
a) 1) Stewart Grubbs catalyst (10 mol‐%), toluene, reflux. 2) HCl, MeOH/THF, 38 %. b) 2,2‐dimethoxypropane, PTSA, acetone/DMF, 86 %. c) 1) H_2_, Pd/C, EtOAC, 72 %. 2) HCl, MeCN, 56 %. c) 1) Dess–Martin periodinane, CH_2_Cl_2_, 94 %. 2) NaBH_4_, MeOH, 0 °C, 71 %. 3) HCl, MeOH, 37 %.

We took advantage of the apparently stereoselective cyclisation of the mixture of epimers of tri‐TBDMS‐protected substrate **1e** to access a single diastereoisomer medium ring product. The RCM product **10e** was challenging to isolate and a significantly improved yield was possible by directly deprotecting the crude mixture (Scheme 6) to give the medium ring triol **2**. In addition to triol **2**, isolated as a single diastereoisomer, a mixture of epimers of the deprotected, uncyclised bisalkene **1e** was recovered (14 %). ^1^H NMR analysis of this uncyclized material showed it was enriched in the minor epimer of the original 60:40 mixture (Figure S1). Further material was isolated from the deprotection, accounting for the mass balance, which could not be identified due to its complex NMR spectrum, but MS analysis showed an *m/z* value corresponding to the dimer metathesis product. It therefore appears that one diastereoisomer of tri‐TBDMS‐protected substrate **1e** undergoes RCM to form the desired medium ring product, while the other instead forms a dimer product and/or degrades. A clear‐cut demonstration of the fate of each epimer in the RCM reaction would require pure samples of each diastereoisomer to be submitted to the reaction conditions individually. However, we were unable to separate the epimers of the substrates **1** at any stage in their synthesis.

**Figure 1:**
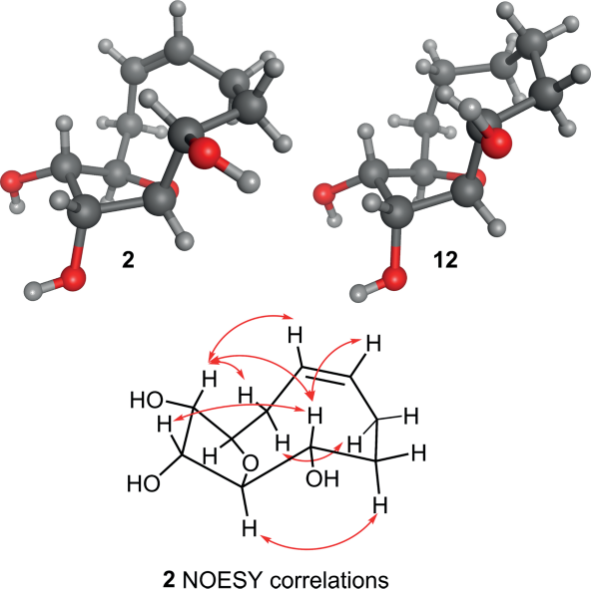
Crystal structures of the unsaturated and saturated medium ring triols (**2** and **12**). The NOESY correlations observed for **2** are indicated for protons separated by four or more bonds. Crystal structure images generated using MOE.^27^

The route allowed efficient synthesis of the desired building block **2** on 0.5 g scale. Triol **2** was selectively protected to give acetonide **11**, which was readily converted into the saturated triol **12** or the *C*‐2 epimer **13** by hydrogenation or sequential oxidation and reduction, respectively. Crystal structures of the unsaturated and saturated medium ring triols (**2** and **12**) were obtained (Figure 1) and showed the (*S*)‐stereochemistry at *C*‐2. In both crystal structures the *C*‐2 alcohol adopts a *pseudo*‐equatorial orientation. The change in conformation of the medium ring between the saturated and unsaturated systems is quite small; however the unsaturated alkane system more clearly disposes the hydrogen atoms in *pseudo*‐axial and *pseudo*‐equatorial orientations. The ^1^H NMR spectrum of triol **2** displayed well‐defined peaks and the observed NOESY correlations were consistent with the crystal structure conformation, suggesting that the solution phase conformation of triol **2** is similar to that in the crystal structure. Unfortunately overlap of CH_2_ signals in the ^1^H NMR spectrum of triol **12** prevented a complete NOESY analysis, but the data available were again consistent with similar solution‐ and solid‐phase conformations.

## Conclusions

A practical synthesis of a novel oxabicyclo[6.2.1]undecenetriol and its epimer and saturated analogue was developed. The key medium ring forming RCM reaction was thoroughly explored and the key factors for success were identified: very bulky silyl protecting groups and higher temperatures were required to provide sufficient conformational bias for the reaction to proceed. The cyclisation showed greater dependency on the substitution of the substrate than on the choice of RCM catalyst or reaction conditions. Crystal structures of the triol and its unsaturated analogue were obtained and NOESY data suggested the medium rings to adopt similar solid and solution phase conformations.

The well‐defined conformations of these nine‐membered ring fused bicyclic systems are potentially relevant for their use as novel scaffolds for drug discovery. A rigid, medium ring core that holds its substituents in fixed positions may allow the construction of molecules with entirely new overall conformations.

## Experimental Section

**General Remarks:** All anhydrous solvents and reagents were obtained from commercial suppliers and used without further purification. Flash column chromatography was carried out using Merck silica gel 60 (0.040–0.063 mm). The Biotage SP4 purification system, with Biotage SNAP KP‐Sil columns, was used for automated purification where stated. Analytical thin layer chromatography (TLC) was performed on aluminium sheets pre‐coated with silica (60 F_254_, Merck) and visualised by short‐wave UV light (254 nm) or potassium permanganate dip. Specific rotations were measured on a Bellingham & Stanley ADP440 polarimeter with a path length of 0.5 or 0.05 dm. Infrared spectra were recorded on a Bruker Alpha‐P FT‐IR spectrometer. ^1^H NMR spectra were recorded at 500 MHz on Bruker AMX500 spectrometers using an internal deuterium lock. Chemical shifts were measured in parts per million (ppm) relative to tetramethylsilane (*δ* = 0 ppm) using the following internal references: CDCl_3_ (*δ*_H_ = 7.26), CD_3_OD (*δ*_H_ = 3.31), [D_6_]DMSO (*δ*_H_ = 2.50) and [D_8_]toluene (*δ*_H_ = 7.00). ^13^C NMR spectra were recorded at 126 MHz on Bruker AMX500 spectrometers using an internal deuterium lock. Chemical shifts were measured in parts per million (ppm) relative to tetramethylsilane (*δ* = 0 ppm) using the following internal references: CDCl_3_ (*δ*_C_ = 77.2), CD_3_OD (*δ*_C_ = 49.0), [D_6_]DMSO (*δ*_C_ = 39.5) and [D_8_]toluene (*δ*_H_ = 137.9).

LC‐MS and HRMS analysis was performed on an Agilent 1200 series HPLC and diode array detector coupled to a 6210 time of flight mass spectrometer with dual multimode APCI/ESI source. The following LC‐MS methods were used. **Method A:** Analytical separation was carried out at 30 °C on a Merck Purospher STAR column (RP‐18e, 30 × 4 mm) using a flow rate of 1.5 mL/min in a 4 min gradient elution with detection at 222 nm. The mobile phase was a mixture of MeOH (solvent A) and water containing formic acid at 0.1 % (solvent B). Gradient elution was as follows: 1:9 (A/B) to 9:1 (A/B) over 1 min, 9:1 (A/B) for 2.5 min, and then reversion back to 1:9 (A/B) over 0.3 min, finally 1:9 (A/B) for 0.2 min. **Method B:** Analytical separation was carried out at 30 °C on a Merck Purospher STAR column (RP‐18e, 30 × 4 mm) using a flow rate of 1.5 mL/min in a 4 min gradient elution with detection at 222 nm. The mobile phase was a mixture of MeOH (solvent A) and water containing formic acid at 0.1 % (solvent B). Gradient elution was as follows: 1:9 (A/B) to 9:1 (A/B) over 0.5 min, 9:1 (A/B) for 3.0 min, and then reversion back to 1:9 (A/B) over 0.3 min, finally 1:9 (A/B) for 0.2 min. HRMS references: caffeine [M + H^+^] 195.087652; hexakis(2,2‐difluoroethoxy)phosphazene [M + H^+^] 622.02896 and hexakis(1*H*,1*H*,3*H*‐tetrafluoropentoxy)phosphazene [M + H^+^] 922.009798.

CCDC 1485214 (for **2**), and 1485215 (for **12**) contain the supplementary crystallographic data for this paper. These data can be obtained free of charge from The Cambridge Crystallographic Data Centre.

**[(3a*S*,4*S*,6*R*,6a*S*)‐6‐(Acetyloxy)‐2,2‐dimethyl‐tetrahydro‐2*H*‐furo[3,4‐*d*][1,3]dioxol‐4‐yl]methyl Acetate (6):** H_2_SO_4_ (conc., 0.13 mL, 0.003 mmol) was added to a stirred slurry of l‐(+)‐ribose (5.0 g, 33 mmol) in acetone (50 mL) under a N_2_ atmosphere. After 6 h the reaction was neutralised with Ca(OH)_2_ (approx. 20 mg), filtered through Celite and the solvents evaporated under vacuum. The residue was purified by column chromatography (60 % hexane in EtOAc) to yield (3a*S*,4*S*,6*S*,6a*S*)‐6‐(hydroxymethyl)‐2,2‐dimethyl‐tetrahydro‐2*H*‐furo[3,4‐*d*][1,3]dioxol‐4‐ol as a yellow oil (5.3 g, 83 %). [*α*]_D_^21^ = +22.3 (*c* = 1.0, CHCl_3_) {ref.^28^ [*α*]_D_^24^ = +21.3 (*c* = 1.02, CHCl_3_)}. ^1^H NMR (500 MHz, MeOD): *δ* = 5.25 (s, 1 H, 4‐H), 4.77 (dd, *J* = 6.0, 1.0 Hz, 1 H, 6a‐H), 4.52 (d, *J* = 6.0 Hz, 1 H, 3a‐H), 4.19 (td, *J* = 5.5, 4.5, 1.0 Hz, 1 H, 6‐H), 3.62 (dd, *J* = 11.5, 4.5 Hz, 1 H, CH_2_), 3.59 (dd, *J* = 11.5, 5.5 Hz, 1 H, CH_2_), 1.44 (s, 3 H, CH_3_), 1.30 (s, 3 H, CH_3_) ppm. ^13^C NMR (126 MHz, MeOD): *δ* = 113.2, 104.0, 88.6, 87.9, 83.4, 64.3, 26.8, 25.0 ppm. ^1^H and ^13^C NMR spectroscopic data match literature values.^28^ Acetic anhydride (55 mL, 580 mmol) was added to a stirred solution of (3a*S*,4*S*,6*S*,6a*S*)‐6‐(hydroxymethyl)‐2,2‐dimethyl‐tetrahydro‐2*H*‐furo[3,4‐*d*][1,3]dioxol‐4‐ol (11 g, 58 mmol) in pyridine (200 mL) under a N_2_ atmosphere at room temp. After 20 h the reaction was quenched at 0 °C with NaHCO_3_ solution (satd. aq., 300 mL). The mixture was extracted with EtOAc (300 mL) and the extract was evaporated under vacuum to afford **6** as a yellow oil (12 g, 75 %). [*α*]_D_^23^ = +57.2 (*c* = 1.0, CHCl_3_) {ref.^29^ enantiomer [*α*]_D_^25^ = –56.7 (*c* = 2.0, CHCl_3_)}. TLC *R*_f_ = 0.2 (20 % EtOAc in hexane). IR (thin film): ν̃ = 2988, 2934, 1745, 1234 cm^–1^. ^1^H NMR (500 MHz, [D_6_]DMSO): *δ* = 5.99 (s, 1 H, 4‐H), 4.81 (dd, *J* = 6.0, 0.5 Hz, 1 H, 6a‐H), 4.77 (d, *J* = 6.0 Hz, 1 H, 3a‐H), 4.36 (apt. t, *J* = 6.5 Hz, 1 H, 6‐H), 4.08 (dd, *J* = 11.5, 6.0 Hz, 1 H, CH_2_), 4.05 (dd, *J* = 11.5, 7.0 Hz, 1 H, CH_2_), 2.04 [s, 3 H, C(O)CH_3_], 2.01 [s, 3 H, C(O)CH_3_], 1.40 [s, 3 H, C(CH_3_)_2_], 1.28 [s, 3 H, C(CH_3_)_2_] ppm. ^13^C NMR (126 MHz, [D_6_]DMSO): *δ* = 170.5, 169.5, 112.5, 102.2, 85.2, 84.9, 81.3, 64.3, 26.6, 25.0, 21.4, 21.0 ppm. LC‐MS (ESI^+^): *m/z* 297 [M + Na^+^]. HRMS (ESI^+^): [M + Na^+^] calcd. for C_12_H_18_O_7_Na 297.0945, found 297.0941.

**[(3a*S*,4*S*,6*R*,6a*R*)‐2,2‐Dimethyl‐6‐(prop‐2‐en‐1‐yl)‐tetrahydro‐2*H*‐furo[3,4‐*d*][1,3]dioxol‐4‐yl]methanol (7):** Allyltrimethylsilane (40 mL, 250 mmol) was added to a stirred slurry of [(3a*S*,4*S*,6*R*,6a*S*)‐6‐(acetyloxy)‐2,2‐dimethyl‐tetrahydro‐2*H*‐furo[3,4‐*d*][1,3]dioxol‐4‐yl]methyl acetate (**6**) (15 g, 56 mmol) and ZnBr_2_ (32 g, 140 mmol) in nitromethane (280 mL) under a N_2_ atmosphere at room temp. After 2 h NaHCO_3_ solution (satd. aq., 30 mL) was added. The mixture was filtered through Celite washing with CH_2_Cl_2_ (500 mL). The filtrate was dried (Na_2_SO_4_), filtered and the solvents evaporated under vacuum. The residue (13 g) was then dissolved in MeOH (100 mL) and stirred under a N_2_ atmosphere at room temp. NaOMe (0.93 g, 17 mmol) was added and the mixture stirred for 1.5 h. NH_4_Cl solution (satd. aq., 50 mL) and brine (150 mL) were added. The mixture was extracted with Et_2_O (3 × 150 mL) and the combined organic layers were dried (MgSO_4_), filtered and the solvents evaporated under vacuum. The residue was purified by automated column chromatography (Biotage 340 g of SNAP KP‐Sil column, 15–25 % EtOAc in hexane) to afford **7** as a yellow oil (7.6 g, 63 %). [*α*]_D_^24^ = +5.8 (*c* = 1.0, CH_2_Cl_2_). TLC *R*_f_ = 0.35 (33 % EtOAc in hexane). IR (thin film): ν̃ = 3467, 2988, 2932, 1077 cm^–1^. ^1^H NMR (500 MHz, CDCl_3_): *δ* = 5.83 (ddt, *J* = 17.0, 10.0, 7.0 Hz, 1 H, 2′‐H), 5.20–5.11 (m, 2 H, 3′‐H), 4.60 (dd, *J* = 7.0, 4.5 Hz, 1 H, 3a‐H), 4.36 (dd, *J* = 7.0, 5.0 Hz, 1 H, 6a‐H), 4.01–3.95 (m, 2 H, 4‐H and 6‐H), 3.82 (dd, *J* = 12.0, 3.5 Hz, 1 H, CH_2_), 3.66 (dd, *J* = 12.0, 4.5 Hz, 1 H, CH_2_), 2.44–2.34 (m, 2 H, 1′‐H), 1.53 (s, 3 H, CH_3_), 1.33 (s, 3 H, CH_3_) ppm. ^13^C NMR (126 MHz, CDCl_3_): *δ* = 133.5, 118.2, 114.8, 84.3, 84.2, 83.7, 81.4, 62.9, 37.8, 27.5, 25.6 ppm. HRMS (ESI^+^): [M + Na^+^] calcd. for C_11_H_18_O_4_Na 237.1102, found 237.1107.

**(1*S*)‐ and (1*R*)‐1‐[(3a*S*,4*S*,6*R*,6a*R*)‐2,2‐Dimethyl‐6‐(prop‐2‐en‐1‐yl)‐tetrahydro‐2*H*‐furo[3,4‐*d*][1,3]dioxol‐4‐yl]pent‐4‐en‐1‐ol (9):** Oxalyl chloride (1.8 mL, 21 mmol) was added slowly to a stirred solution of DMSO (2.5 mL, 35 mmol) in CH_2_Cl_2_ (140 mL) at –78 °C under a N_2_ atmosphere. After 20 min at –78 °C a solution of [(3a*S*,4*S*,6*R*,6a*R*)‐2,2‐dimethyl‐6‐(prop‐2‐en‐1‐yl)‐tetrahydro‐2*H*‐furo[3,4‐*d*][1,3]dioxol‐4‐yl]methanol (**7**) (3.0 g, 14 mmol) in CH_2_Cl_2_ (20 mL) was added slowly over 45 min at –78 °C. The mixture was stirred for 45 min at –78 °C and NEt_3_ (7.8 mL, 56 mmol) was added slowly over 20 min at –78 °C. Water (30 mL) was added at –78 °C and the mixture was warmed to room temp. Further water (120 mL) was added and the mixture was separated. The aqueous layer was further extracted with EtOAc (2 × 150 mL) and the combined organic layers were dried (Na_2_SO_4_), filtered through a silica pad and the solvents evaporated under vacuum to afford the crude aldehyde (**8**) as a yellow oil (3.4 g). A solution of 4‐bromo‐1‐butene (8.5 mL, 84 mmol) in THF (20 mL) was added to stirred Mg turnings (2.2 g, 92 mmol) under a N_2_ atmosphere. After 30 min further THF (20 mL) was added to afford but‐3‐en‐1‐ylmagnesium bromide as a black slurry (2.1 m in THF). Crude aldehyde **8** (3.4 g) was dissolved in THF (47 mL) and stirred at 0 °C under a N_2_ atmosphere. Freshly prepared but‐3‐en‐1‐ylmagnesium bromide (2.1 m in THF, 33 mL, 70 mmol) was added. The mixture was warmed to room temp. and stirred for 2 h. The mixture was cooled to 0 °C and NH_4_Cl solution (satd. aq., 60 mL) and water (30 mL) were added. The mixture was extracted with Et_2_O (3 × 90 mL) and the combined organic layers were dried (Na_2_SO_4_), filtered and the solvents evaporated under vacuum. The residue was purified by column chromatography (25–35 % EtOAc in hexane) to afford a 60:40 epimeric mixture of **9** as a colourless oil (2.0 g, 53 %). TLC *R*_f_ = 0.26 (14 % EtOAc in hexane). IR (thin film): ν̃ = 3486, 3077, 2982, 2935, 1642, 1074 cm^–1^. ^1^H NMR (500 MHz, CDCl_3_): *δ* = (diastereomeric ratio 60:40) 5.88–5.78 (m, 2 H, 4′‐H and 2′′‐H), 5.19–5.12 (m, 2 H, 3′′‐H), 5.09–5.04 (m, 1 H, 5′‐H), 5.00–4.97 (m, 1 H, 5′‐H), 4.68 (dd, *J* = 7.0, 4.5 Hz, 0.6 H, 6a‐H), 4.52 (dd, *J* = 7.0, 4.5 Hz, 0.4 H, 6a‐H), 4.36 (dd, *J* = 7.0, 4.5 Hz, 0.4 H, 3a‐H), 4.32 (dd, *J* = 7.0, 5.0 Hz, 0.6 H, 3a‐H), 4.01–3.92 (m, 1 H, 4‐H), 3.86–3.75 (m, 1.6 H, 6‐H and 1′‐H), 3.63–3.57 (m, 0.4 H, 1′‐H), 2.45–2.34 (m, 2 H, 1′′‐H), 2.34–2.25 (m, 1 H, 3′‐H), 2.23–2.13 (m, 1 H, 3′‐H), 1.66–1.59 (m, 2 H, 2′‐H), 1.53 (s, 3 H, CH_3_), 1.34 (s, 1.8 H, CH_3_), 1.33 (s, 1.2 H, CH_3_) ppm. ^13^C NMR (126 MHz, CDCl_3_): *δ* = 138.3, 138.2, 133.50, 133.48, 118.27, 118.25, 115.3, 115.2, 114.8, 114.7, 86.8, 86.7, 84.2, 84.0, 83.5, 83.2, 82.4, 79.8, 71.7, 70.2, 37.8, 37.7, 32.9, 31.5, 30.1, 30.0, 27.6, 27.5, 25.63, 25.61 ppm. HRMS (ESI^+^): [M + Na^+^] calcd. for C_15_H_24_O_4_Na 291.1572, found 295.1565.

**(1*S*)‐ and (1*R*)‐(3a*S*,4*S*,6*R*,6a*R*)‐2,2‐Dimethyl‐4‐[1‐(naphthalen‐2‐ylmethoxy)pent‐4‐en‐1‐yl]‐6‐(prop‐2‐en‐1‐yl)‐tetrahydro‐2*H*‐furo[3,4‐*d*][1,3]dioxole (1a):** NaH (60 % dispersion in oil, 0.11 g, 2.8 mmol) was added to a stirred solution of a 60:40 epimeric mixture of (1*S*)‐ and (1*R*)‐1‐[(3a*S*,4*S*,6*R*,6a*R*)‐2,2‐dimethyl‐6‐(prop‐2‐en‐1‐yl)‐tetrahydro‐2*H*‐furo[3,4‐*d*][1,3]dioxol‐4‐yl]pent‐4‐en‐1‐ol (**9**) (0.24 g, 0.89 mmol) in DMF (8.9 mL) at 0 °C under a N_2_ atmosphere. After 1 h at 0 °C, 2‐(bromomethyl)naphthalene (0.40 g, 1.8 mmol) and tetrabutylammonium iodide (0.33 g, 0.89 mmol) were added and the mixture was warmed to room temp. After 1 h NaHCO_3_ solution (satd. aq., 10 mL) and water (20 mL) were added at 0 °C and the mixture was extracted with Et_2_O (3 × 30 mL). The combined organic layers were washed with brine (300 mL), dried (MgSO_4_), filtered and the solvents evaporated under vacuum. The residue was purified by column chromatography (5 % EtO_2_ in hexane) to afford a 60:40 epimeric mixture of **1a** as a colourless oil (0.31 g, 85 %). TLC *R*_f_ = 0.63 (15 % EtOAc in hexane). IR (thin film): ν̃ = 3061, 2981, 2933, 1641, 1077 cm^–1^. ^1^H NMR (500 MHz, CDCl_3_): *δ* = (diastereomeric ratio 60:40) 7.87–7.82 (m, 3 H, ArH), 7.81–7.79 (m, 1 H, ArH), 7.51–7.46 (m, 3 H, ArH), 5.95–5.74 (m, 2 H, 4′‐H and 2′′‐H), 5.19–5.08 (m, 2 H, 3′′‐H), 5.06–4.95 (m, 2 H, 5′‐H), 4.88 (d, *J* = 11.5 Hz, 0.6 H, CH_2_), 4.84 (d, *J* = 11.5 Hz, 0.4 H, CH_2_), 4.81–4.74 (m, 1.6 H, 6a‐H and CH_2_), 4.48 (dd, *J* = 7.0, 4.5 Hz, 0.4 H, 3a‐H), 4.35 (dd, *J* = 7.0, 5.0 Hz, 0.6 H, 3a‐H), 4.33 (dd, *J* = 7.0, 5.5 Hz, 0.4 H, 6a‐H), 4.06 (dd, *J* = 5.5, 4.5 Hz, 0.4 H, 4‐H), 4.00–3.95 (m, 1 H, 4‐H and 6‐H), 3.90 (dt, *J* = 6.5, 5.5 Hz, 0.6 H, 6‐H), 3.71 (ddd, *J* = 8.0, 4.5, 3.0 Hz, 0.6 H, 1′‐H), 3.53 (dt, *J* = 7.5, 5.5 Hz, 0.4 H, 1′‐H), 2.48–2.37 (m, 2 H, 1′′‐H), 2.33–2.23 (m, 1 H, 3′‐H), 2.23–2.12 (m, 1 H, 3′‐H), 1.81–1.69 (m, 1.2 H, 2′‐H), 1.69–1.59 (m, 0.8 H, 2′‐H), 1.55 (s, 2 H, CH_3_), 1.54 (s, 1 H, CH_3_), 1.35 (s, 1.8 H, CH_3_), 1.32 (s, 1.2 H, CH_3_) ppm. ^13^C NMR (126 MHz, CDCl_3_): *δ* = 138.5, 138.3, 136.4, 134.0, 133.9, 133.4, 133.1, 128.2, 128.0, 127.8, 126.5, 126.2, 126.1, 125.9, 117.8, 117.7, 115.2, 115.1, 114.7, 114.5, 87.0, 85.7, 84.1, 83.8, 83.4, 82.5, 80.8, 78.8, 78.0, 73.7, 72.8, 37.9, 31.0, 30.0, 29.9, 29.6, 27.6, 27.6, 25.7, 25.7 ppm. LC‐MS (ESI^+^): *m/z* 409 [M + H^+^]. HRMS (ESI^+^): [M + H^+^] calcd. for C_26_H_33_O_4_ 409.2373, found 409.2364. HPLC (Method A): *t*_R_ = 2.19 min; purity (AUC) ≥ 95 %.

**(1*S*)‐ and (1*R*)‐(2S,3R,4R,5R)‐3,4‐Bis(benzyloxy)‐2‐[1‐(naphthalen‐2‐ylmethoxy)pent‐4‐en‐1‐yl]‐5‐(prop‐2‐en‐1‐yl)oxolane (1b):** Aqueous HCl (6 n, 2.4 mL) was added to a stirred solution of (1*S*)‐ and (1*R*)‐(3a*S*,4*S*,6*R*,6a*R*)‐2,2‐dimethyl‐4‐[1‐(naphthalen‐2‐ylmethoxy)pent‐4‐en‐1‐yl]‐6‐(prop‐2‐en‐1‐yl)‐tetrahydro‐2*H*‐furo[3,4‐*d*][1,3]dioxole (**1a**) (0.050 g, 0.12 mmol) in MeOH (2.4 mL) at 0 °C under a N_2_ atmosphere. The solution was stirred at room temp. for 1 h then NaOH solution (6 m aq., 2.4 mL) was added at 0 °C and the mixture was absorbed onto silica. Purification by column chromatography (5 % MeOH in CH_2_Cl_2_) afforded (1*S*)‐ and (1*R*)‐(2*R*,3*R*,4*S*,5*R*)‐2‐[1‐(naphthalen‐2‐ylmethoxy)pent‐4‐en‐1‐yl]‐5‐(prop‐2‐en‐1‐yl)oxolane‐3,4‐diol as a colourless oil (0.024 g, 54 %). TLC *R*_f_ = 0.36 (5 % MeOH in CH_2_Cl_2_). IR (thin film): ν̃ = 3387, 3074, 2923, 1641, 1097 cm^–1^. ^1^H NMR (500 MHz, CDCl_3_): *δ* = (diastereomeric ratio 60:40) 7.86–7.81 (m, 3 H, ArH), 7.79–7.76 (m, 1 H, ArH), 7.51–7.44 (m, 3 H, ArH), 5.93–5.77 (m, 2 H, 4′‐H and 2′′‐H), 5.17–4.95 (m, 4 H, 5′‐H and 3′′‐H), 4.78 (s, 2 H, CH_2_), 4.16–4.11 (m, 0.6 H, 3‐H), 4.05–4.00 (m, 0.4 H, 3‐H), 3.94 (t, *J* = 5.0 Hz, 0.4 H, 2‐H), 3.85–3.78 (m, 2.6 H, 2‐H, 4‐H and 5‐H), 3.67 (dt, *J* = 7.0, 4.5 Hz, 0.6 H, 1′‐H), 3.62 (dt, *J* = 8.0, 4.5 Hz, 0.4 H, 1′‐H), 2.60 (d, *J* = 3.5 Hz, 0.6 H, OH), 2.51 (apt. d, *J* = 4.0 Hz, 1 H, OH), 2.46 (d, *J* = 4.5 Hz, 0.4 H, OH), 2.43–2.31 (m, 2 H, 1′′‐H), 2.31–2.11 (m, 2 H, 3′‐H), 1.84–1.64 (m, 2 H, 2′′‐H) ppm. ^13^C NMR (126 MHz, CDCl_3_): *δ* = 138.5, 138.4, 136.01, 135.99, 134.18, 134.16, 133.40, 133.37, 133.1, 128.38, 128.35, 128.0, 127.84, 126.82, 126.7, 126.32, 126.30, 126.13, 126.09, 126.01, 117.74, 117.73, 115.12, 115.11, 85.6, 84.2, 82.6, 82.3, 79.3, 78.5, 74.7, 74.5, 73.3, 72.9, 72.1, 71.7, 37.9, 37.8, 30.7, 30.2, 29.5, 29.4 ppm. LC‐MS (ESI^+^): *m/z* 369 [M + H^+^]. HRMS (ESI^+^): [M + H^+^] calcd. for C_23_H_29_O_4_ 369.2060, found 369.2066. HPLC (Method A): *t*_R_ = 1.78 min; purity (AUC) = 93 %. NaH (60 % in oil, 0.013 g, 0.33 mmol) was added to a stirred solution of (1*S*)‐ and (1*R*)‐(2*R*,3*R*,4*S*,5*R*)‐2‐[1‐(naphthalen‐2‐ylmethoxy)pent‐4‐en‐1‐yl]‐5‐(prop‐2‐en‐1‐yl)oxolane‐3,4‐diol (0.024 g, 0.07 mmol) in DMF (1.5 mL) at 0 °C under a N_2_ atmosphere. The solution was warmed to room temp. and stirred for 30 min then benzyl bromide (0.033 g, 0.20 mmol) was added. After 2 h NaHCO_3_ solution (satd. aq., 2 mL) and water (2 mL) were added at 0 °C and the mixture was extracted with Et_2_O (3 × 4 mL). The combined organic layers were washed with brine (6 mL), dried (Na_2_SO_4_), filtered and the solvents evaporated under vacuum. The residue was purified by column chromatography (15 % EtOAc in hexane) to afford a 60:40 epimeric mixture of **1b** as a colourless oil (0.029 g, 76 %). TLC *R*_f_ = 0.30 (10 % EtOAc in hexane). IR (thin film): ν̃ = 3063, 3030, 2917, 1640, 1124, 1095 cm^–1^. ^1^H NMR (500 MHz, CDCl_3_): *δ* = (diastereomeric ratio 60:40) 7.86–7.77 (m, 3 H, ArH), 7.75–7.72 (m, 1 H, ArH), 7.52–7.45 (m, 2 H, ArH), 7.41 (ddd, *J* = 8.5, 6.5, 1.5 Hz, 1 H, ArH), 7.33–7.21 (m, 10 H, ArH), 5.90–5.73 (m, 2 H, 4′‐H and 2′′‐H), 5.10–4.94 (m, 4 H, 5′‐H and 3′′‐H), 4.80–4.70 [m, 1.6 H, C*H*_2_(C_10_H_7_)], 4.65 [d, *J* = 11.7 Hz, 0.4 H, C*H*_2_(C_10_H_7_)], 4.60–4.37 (m, 4 H, C*H*_2_Ph), 4.18 (t, *J* = 4.0 Hz, 0.4 H, 2‐H), 4.12–4.10 (m, 0.6 H, 2‐H), 4.09–4.04 (m, 1 H, 5‐H), 4.00 (dd, *J* = 5.0, 3.5 Hz, 0.6 H, 3‐H), 3.85 (t, *J* = 5.5, 4.5 Hz, 0.4 H, 3‐H), 3.60–3.54 (m, 1.6 H, 4‐H and 1′‐H), 3.50–3.46 (m, 0.4 H, 1′‐H), 2.46–2.36 (m, 1 H, 1′′‐H), 2.31–2.17 (m, 2 H, 3′‐H and 1′′‐H), 2.17–2.07 (m, 1 H, 3′‐H), 1.74–1.52 (m, 2 H, 2′‐H) ppm. ^13^C NMR (126 MHz, CDCl_3_): *δ* = 138.5, 138.4, 138.2, 138.0, 137.98, 137.97, 136.2, 134.53, 134.52, 133.39, 133.38, 133.08, 133.07, 128.48, 128.45, 128.21, 128.16, 128.1, 128.0, 127.93, 127.91, 127.8, 126.7, 126.6, 126.24, 126.17, 126.1, 126.0, 117.4, 115.1, 115.0, 85.4, 83.4, 81.0, 80.3, 80.1, 79.5, 78.7, 78.2, 77.2, 76.6, 73.6, 72.7, 72.3, 72.1, 71.9, 71.9, 37.9, 37.8, 30.7, 30.1, 29.9, 29.6 ppm. LC‐MS (ESI^+^): *m/z* 549 [M + H^+^]. HRMS (ESI^+^): [M + H^+^] calcd. for C_37_H_41_O_4_ 549.2999, found 549.2991. HPLC (Method A): *t*_R_ = 2.06 min; purity (AUC) ≥ 95 %.

**(1*R*)‐ and (1*S*)‐*tert*‐Butyl[({(2*R*,3*R*,4*S*,5*S*)‐4‐[(*tert*‐butyldimethylsilyl)oxy]‐5‐[1‐(naphthalen‐2‐ylmethoxy)pent‐4‐en‐1‐yl]‐2‐(prop‐2‐en‐1‐yl)oxolan‐3‐yl}oxy)]dimethylsilane (1c):** BF_3_ **·**OEt_2_ (0.060 mL, 0.49 mmol) and ethanedithiol (0.041 mL, 0.49 mmol) were added to a stirred solution of (1*S*)‐ and (1*R*)‐(3a*S*,4*S*,6*R*,6a*R*)‐2,2‐dimethyl‐4‐[1‐(naphthalen‐2‐ylmethoxy)pent‐4‐en‐1‐yl]‐6‐(prop‐2‐en‐1‐yl)‐tetrahydro‐2*H*‐furo[3,4‐*d*][1,3]dioxole (**1a**) (0.10 g, 0.24 mmol) in CH_2_Cl_2_ at 0 °C under a N_2_ atmosphere. After 30 min at 0 °C NaHCO_3_ solution (satd. aq., 10 mL) was added and the mixture was extracted with CH_2_Cl_2_ (3 × 10 mL). The combined organic layers were dried (Na_2_SO_4_), filtered and the solvents evaporated under vacuum. The residue was purified by column chromatography (40 % EtOAc in hexane) to afford (1*S*)‐ and (1*R*)‐(2*R*,3*R*,4*S*,5*R*)‐2‐[1‐(naphthalen‐2‐ylmethoxy)pent‐4‐en‐1‐yl]‐5‐(prop‐2‐en‐1‐yl)oxolane‐3,4‐diol (0.049 g, 55 %). 2,6‐Lutidine (0.071 mL, 0.61 mmol) and *tert*‐butyldimethylsilyl trifluoromethanesulfonate (0.11 mL, 0.49 mmol) were added to a stirred solution of (1*S*)‐ and (1*R*)‐(2*R*,3*R*,4*S*,5*R*)‐2‐[1‐(naphthalen‐2‐ylmethoxy)pent‐4‐en‐1‐yl]‐5‐(prop‐2‐en‐1‐yl)oxolane‐3,4‐diol (0.045 g, 0.12 mmol) in CH_2_Cl_2_ (2.0 mL) under a N_2_ atmosphere at room temp. The solution was stirred at room temp. for 72 h then water (5.0 mL) was added. The mixture was extracted with CH_2_Cl_2_ (3 × 5.0 mL) and the combined organic layers were dried (Na_2_SO_4_), filtered and the solvents evaporated under vacuum. The residue was purified by column chromatography (5 % EtOAc in hexane) to afford a 60:40 epimeric mixture of **1c** as a colourless oil (0.064 g, 89 %). TLC *R*_f_ = 0.73 (15 % EtOAc in hexane). IR (thin film): ν̃ = 2928, 2856, 1641, 836, 774 cm^–1^. ^1^H NMR (500 MHz, CDCl_3_): *δ* = (diastereomeric ratio 60:40) 7.85–7.79 (m, 3 H, ArH), 7.78 (s, 1 H, ArH), 7.50–7.44 (m, 3 H, ArH), 6.01–5.77 (m, 2 H, 4′‐H and 2′′‐H), 5.15–4.94 (m, 4 H, 5′‐H and 3′′‐H), 4.83–4.73 (m, 1.6 H, CH_2_), 4.68–4.63 (m, 0.4 H, CH_2_), 4.07 (dd, *J* = 4.5, 2.0 Hz, 0.6 H, 3‐H), 3.97–3.94 (m, 0.8 H, 2‐H and 3‐H), 3.93 (dd, *J* = 3.5, 2.0 Hz, 0.6 H, 2‐H), 3.91–3.85 (m, 1 H, 5‐H), 3.73 (dd, *J* = 7.0, 4.5 Hz, 0.4 H, 4‐H), 3.68 (dd, *J* = 7.5, 4.5 Hz, 0.6 H, 4‐H), 3.51 (ddd, *J* = 8.0, 4.5, 3.5 Hz, 0.6 H, 1′‐H), 3.47 (td, *J* = 6.5, 3.0 Hz, 0.4 H, 1′‐H), 2.45–2.37 (m, 1 H, 1′′‐H), 2.31–2.06 (m, 3 H, 3′‐H and 1′′‐H), 1.86–1.71 (m, 1.4 H, 2′‐H), 1.62 (dddd, *J* = 14.0, 9.5, 6.4, 4.5 Hz, 0.6 H, 2′‐H), 0.89 (s, 5 H, CCH_3_), 0.87 (s, 4 H, CCH_3_), 0.86 (s, 4 H, CCH_3_), 0.84 (s, 5 H, CCH_3_), 0.07 (s, 1.8 H, SiCH_3_), 0.06 (s, 1.8 H, SiCH_3_), 0.00 (s, 1.2 H, SiCH_3_), –0.00 (s, 1.2 H, SiCH_3_), –0.03 (s, 1.8 H, SiCH_3_), –0.03 (s, 1.2 H, SiCH_3_), –0.05 (s, 1.2 H, SiCH_3_), –0.10 (s, 1.8 H, SiCH_3_) ppm. ^13^C NMR (126 MHz, CDCl_3_): *δ* = 138.48, 138.46, 136.3, 136.2, 135.53, 135.45, 133.43, 133.41, 133.07, 133.06, 128.23, 128.19, 128.0, 127.82, 127.81, 126.6, 126.4, 126.20, 126.18, 126.1, 126.02, 125.95, 125.9, 117.0, 116.8, 115.1, 115.0, 87.2, 85.8, 80.4, 79.9, 79.2, 78.0, 76.4, 76.2, 74.1, 73.4, 73.2, 72.1, 37.9, 37.7, 30.7, 30.1, 30.0, 29.2, 26.11, 26.05, 26.0, 18.21, 18.20, 18.17, 18.1, –3.8, –3.9, –4.07, –4.11, –4.2, –4.3, –4.4 ppm. LC‐MS (ESI^+^): *m/z* 597 [M + H^+^]. HRMS (ESI^+^): [M + H^+^] calcd. for C_35_H_56_O_4_Si_2_ 597.3790, found 597.3774. HPLC (Method A): *t*_R_ = 2.50 min; purity (AUC) ≥ 95 %.

**(1*R*)‐ and (1*S*)‐*tert*‐Butyl({[(2*R*,3*R*,4*S*,5*S*)‐4‐[(*tert*‐butyldiphenylsilyl)oxy]‐5‐[1‐[(*tert*‐butyldiphenylsilyl)oxy]pent‐4‐en‐1‐yl]‐2‐(prop‐2‐en‐1‐yl)oxolan‐3‐yl]oxy})diphenylsilane (1d):** TFA (90 % aq., 10 mL) was added to (1*S*)‐ and (1*R*)‐1‐[(3a*S*,4*S*,6*R*,6a*R*)‐2,2‐dimethyl‐6‐(prop‐2‐en‐1‐yl)‐tetrahydro‐2*H*‐furo[3,4‐*d*][1,3]dioxol‐4‐yl]pent‐4‐en‐1‐ol (**9**) (0.63 g, 2.4 mmol) and the resultant solution stirred for 20 min at room temp. then evaporated under vacuum. The residue was purified by column chromatography (5 % MeOH in CH_2_Cl_2_) to afford a 60:40 epimeric mixture of (1*S*)‐ and (1*R*)‐(2*S*,3*R*,4*S*,5*R*)‐2‐[1‐hydroxypent‐4‐en‐1‐yl]‐5‐(prop‐2‐en‐1‐yl)oxolane‐3,4‐diol as a yellow gum (0.47 g, 86 %). TLC *R*_f_ = 0.31 (5 % MeOH in CH_2_Cl_2_). IR (thin film): ν̃ = 3367, 3077, 2920, 1641, 1099, 914 cm^–1^. ^1^H NMR (500 MHz, CDCl_3_): *δ* = (diastereomeric ratio 60:40) 5.89–5.78 (m, 2 H, 2′′‐H and 4′‐H), 5.19–4.96 (m, 4 H, 3′′‐H and 5′‐H), 4.12 (app‐q, *J* = 5.5, 3.5 Hz, 0.6 H, 3‐H), 4.05 (app‐q, *J* = 5.5 Hz, 0.4 H, 3‐H), 3.89–3.75 (m, 2.6 H, 1′‐H, 4‐H and 5‐H), 3.70–3.66 (m, 1 H, 2‐H), 3.66–3.60 (m, 0.4 H, 1′‐H), 2.88 (d, *J* = 3.5 Hz, 0.6 H, 3‐OH), 2.85 (d, *J* = 5.5 Hz, 0.4 H, 3‐OH), 2.78 (d, *J* = 4.5 Hz, 0.6 H, 4‐OH), 2.72 (d, *J* = 5.0 Hz, 0.4 H, 4‐OH), 2.45–2.22 (m, 3 H, 1′′‐H and 3′‐H), 2.22–2.12 (m, 1 H, 3′‐H), 2.05 (d, *J* = 8.0 Hz, 0.4 H, 1′‐OH), 1.79 (s, 0.6 H, 1′‐OH), 1.72–1.56 (m, 2 H, 2′‐H) ppm. ^13^C NMR (126 MHz, CDCl_3_): *δ* = 138.25, 138.24, 133.9, 133.7, 118.3, 118.2, 115.3, 115.2, 86.2, 85.7, 82.5, 82.3, 74.6, 74.4, 72.1, 71.5, 71.1, 70.9, 37.63, 37.56, 33.3, 31.9, 30.11, 30.07 ppm. LC‐MS (ESI^+^): *m/z* 229 [M + H^+^], 211.13 [M – H_2_O + H]^+^. HRMS (ESI^+^): [M + H^+^] calcd. for C_12_H_21_O_4_ 229.1434, found 229.1437. *tert*‐Butyldiphenylsilyl trifluoromethanesulfonate (0.57 mL, 1.9 mmol) and 2,6‐lutidine (0.22 mL, 1.9 mmol) were added to a stirred solution of (1*S*)‐ and (1*R*)‐(2*S*,3*R*,4*S*,5*R*)‐2‐[1‐hydroxypent‐4‐en‐1‐yl]‐5‐(prop‐2‐en‐1‐yl)oxolane‐3,4‐diol (0.25 g, 0.93 mmol) in CH_2_Cl_2_ (3.1 mL) under a N_2_ atmosphere in a sealed vessel. The solution was heated at 40 °C for 72 h then cooled and poured into NaHCO_3_ solution (satd. aq., 10 mL). The mixture was extracted with CH_2_Cl_2_ (3 × 10 mL) and the combined organic layers were dried (Na_2_SO_4_), filtered and the solvents evaporated under vacuum. The residue was purified by column chromatography (5 % EtOAc in hexane) to afford a 60:40 epimeric mixture of **1d** as a white foam (0.37 g, 79 %). TLC *R*_f_ = 0.51 (5 % EtOAc in hexane). IR (thin film): ν̃ = 3072, 2931, 2857, 1110, 702 cm^–1^. ^1^H NMR (500 MHz, CDCl_3_): *δ* = (diastereomeric ratio 60:40) 7.78–7.49 (m, 11 H, ArH), 7.48–7.18 (m, 19 H, ArH), 5.58–5.46 (m, 0.6 H, 2′′‐H), 5.30–5.14 (m, 0.8 H, 2′′‐H and 4′‐H), 5.03–4.91 (m, 0.6 H, 4′‐H), 4.83–4.77 (m, 0.6 H, 3′′‐H), 4.76–4.71 (m, 0.6 H, 3′′‐H), 4.71–4.67 (m, 0.6 H, 3′′‐H), 4.65–4.62 (m, 0.4 H, 5′‐H), 4.62–4.61 (m, 0.2 H, 3′′‐H), 4.57–4.55 (m, 0.6 H, 3‐H), 4.55–4.53 (m, 0.2 H, 5′‐H), 4.52–4.48 (m, 0.8 H, 5′‐H), 4.32 (app‐dd, *J* = 3.5, 1.5 Hz, 0.3 H, 5‐H), 4.30–4.26 (m, 0.6 H, 3‐H and 5′‐H), 4.23 (dd, *J* = 5.0, 3.5 Hz, 0.3 H, 2‐H), 4.11–4.04 (m, 1.2 H, 4‐H and 5‐H), 3.98 (dt, *J* = 7.5, 5.0 Hz, 0.4 H, 5‐H), 3.81 (t, *J* = 4.5 Hz, 0.4 H, 4‐H), 3.77–3.75 (m, 0.6 H, 2‐H), 3.57–3.53 (m, 0.4 H, 2′‐H), 2.93 (dt, *J* = 10.0, 4.0, 2.5 Hz, 0.6 H, 1′‐H), 2.08–2.00 (m, 0.6 H, 1′′‐H), 1.77–1.70 (m, 0.4 H, 1′′‐H), 1.63–1.52 (m, 1 H, 1′′‐H), 1.52–1.50 (m, 0.4 H, 3′‐H), 1.45–1.36 (m, 0.6 H, 2′‐H), 1.30–1.14 (m, 1.4 H, 2′‐H and 3′‐H), 1.10 (s, 11 H, CH_3_), 1.04 (s, 3.6 H, CH_3_), 1.00 (s, 3.6 H, CH_3_), 0.96 (s, 3.6 H, CH_3_), 0.95–0.82 (m, 0.6 H, 3′‐H), 0.80–0.67 (m, 6.4 H, 2′‐H and CH_3_) ppm. ^13^C NMR (126 MHz, CDCl_3_): *δ* = 138.8, 138.1, 136.4, 136.3, 136.24, 136.21, 136.18, 136.10, 135.99, 135.9, 135.8, 135.6, 134.80, 134.77, 134.7, 134.5, 134.30, 134.25, 134.2, 134.0, 133.9, 133.8, 133.7, 133.6, 129.83, 129.80, 129.74, 129.70, 129.68, 129.6, 129.5, 129.3, 127.80, 127.76, 127.73, 127.69, 127.66, 127.6, 127.5, 127.3, 116.7, 115.9, 113.9, 113.8, 85.2, 84.9, 81.5, 79.6, 77.4, 76.9, 76.2, 74.4, 73.7, 73.4, 37.9, 36.8, 31.9, 31.2, 29.44, 29.40, 27.33, 27.28, 27.23, 27.22, 27.1, 19.64, 19.58, 19.57, 19.51, 19.48, 19.3 ppm. LC‐MS (ESI^+^): *m/z* 965 [M + Na]^+^. HRMS (ESI^+^): [M + Na^+^] calcd. for C_60_H_74_O_4_Si_3_Na 965.4787, found 965.4785. HPLC (Method A): *t*_R_ = 3.69 min; purity (AUC) = 95 %.

**(1*R*)‐ and (1*S*)‐*tert*‐Butyl({[(2*R*,3*R*,4*S*,5*S*)‐4‐[(*tert*‐butyldimethylsilyl)oxy]‐5‐[1‐[(*tert*‐butyldimethylsilyl)oxy]pent‐4‐en‐1‐yl]‐2‐(prop‐2‐en‐1‐yl)oxolan‐3‐yl]oxy})dimethylsilane (1e):** (1*S*)‐ and (1*R*)‐(2*S*,3*R*,4*S*,5*R*)‐2‐[1‐Hydroxypent‐4‐en‐1‐yl]‐5‐(prop‐2‐en‐1‐yl)oxolane‐3,4‐diol was prepared as for **1d**. *tert*‐Butyldimethylsilyl trifluoromethanesulfonate (3.7 mL, 16 mmol) and 2,6‐lutidine (1.9 mL, 16 mmol) were added to a stirred solution of (1*S*)‐ and (1*R*)‐(2*S*,3*R*,4*S*,5*R*)‐2‐[1‐hydroxypent‐4‐en‐1‐yl]‐5‐(prop‐2‐en‐1‐yl)oxolane‐3,4‐diol (0.61 g, 2.7 mmol) in CH_2_Cl_2_ (8.9 mL) under a N_2_ atmosphere in a sealed vessel. The solution was heated at 40 °C for 18 h then cooled and poured into NaHCO_3_ solution (satd. aq., 20 mL). The mixture was extracted with CH_2_Cl_2_ (3 × 20 mL) and the combined organic layers were dried (Na_2_SO_4_), filtered and the solvents evaporated under vacuum. The residue was purified by column chromatography (2 % EtOAc in hexane) to afford a 60:40 epimeric mixture of **1e** as a colourless oil (1.4 g, 88 %). TLC *R*_f_ 0.63 (5 % EtOAc in hexane). IR (thin film): ν̃ = 2953, 2929, 2896, 2857, 833, 822, 771 cm^–1^. ^1^H NMR (500 MHz, CDCl_3_): *δ* = (diastereomeric ratio 60:40) 6.01–5.74 (m, 2 H, 2′′‐H and 4′‐H), 5.14–4.92 (m, 4 H, 3′′‐H and 5′‐H), 4.06 (dd, *J* = 4.0, 2.5 Hz, 0.6 H, 3‐H), 3.93 (dd, *J* = 5.0, 1.5 Hz, 0.4 H, 3‐H), 3.90–3.81 (m, 2 H, 2‐H and 5‐H), 3.72–3.58 (m, 2 H, 1′‐H and 4‐H), 2.47–2.34 (m, 1 H, 1′′‐H), 2.20–2.00 (m, 3 H, 1′′‐H and 3′‐H), 1.91–1.82 (m, 0.4 H, 2′‐H), 1.72–1.63 (m, 0.6 H, 2′‐H), 1.63–1.53 (m, 0.6 H, 2′‐H), 1.51–1.42 (m, 0.4 H, 2′‐H), 1.01–0.82 [m, 27 H, C(CH_3_)_3_], 0.12–0.02 [m, 18 H, Si(CH_3_)_2_] ppm. ^13^C NMR (126 MHz, CDCl_3_): *δ* = 138.7, 138.6, 135.6, 135.4, 117.0, 116.7, 114.9, 114.7, 86.8, 86.3, 80.3, 79.8, 76.17, 75.9, 74.8, 72.9, 72.5, 72.3, 37.9, 37.1, 33.3, 32.9, 30.0, 29.1, 26.22, 26.19, 26.13, 26.11, 26.07, 18.3, 18.24, 18.22, 18.17, –3.8, –4.0, –4.1, –4.2, –4.3 ppm. LC‐MS (ESI^+^): *m/z* 593 [M + Na]^+^. HRMS (ESI^+^): [M + Na^+^] calcd. for C_30_H_62_O_4_Si_3_Na 593.3848, found 593.3831.

**(1*S*)‐ and (1*R*)‐Trimethyl({[(2*S*,3*S*,4*R*,5*R*)‐5‐(prop‐2‐en‐1‐yl)‐4‐[(trimethylsilyl)oxy]‐5‐[1‐[(trimethylsilyl)oxy]pent‐4‐en‐1‐yl]oxolan‐3‐yl]oxy})silane (1f):** (1*S*)‐ and (1*R*)‐(2*S*,3*R*,4*S*,5*R*)‐2‐[1‐Hydroxypent‐4‐en‐1‐yl]‐5‐(prop‐2‐en‐1‐yl)oxolane‐3,4‐diol was prepared as for **1d**. Trimethylsilyl trifluoromethanesulfonate (0.48 mL, 2.6 mmol) and 2,6‐lutidine (0.48 mL, 2.6 mmol) were added to a stirred solution of (1*S*)‐ and (1*R*)‐(2*S*,3*R*,4*S*,5*R*)‐2‐[1‐hydroxypent‐4‐en‐1‐yl]‐5‐(prop‐2‐en‐1‐yl)oxolane‐3,4‐diol (0.10 g, 0.44 mmol) in CH_2_Cl_2_ (4.4 mL) under a N_2_ atmosphere in a sealed vessel. The solution was stirred at room temp. for 24 h then cooled and poured into NaHCO_3_ solution (satd. aq., 10 mL). The mixture was extracted with CH_2_Cl_2_ (3 × 10 mL) and the combined organic layers were dried (Na_2_SO_4_), filtered and the solvents evaporated under vacuum. The residue was purified by column chromatography (5 % EtOAc in hexane) to afford a 60:40 epimeric mixture of **1f** as a colourless oil (0.14 g, 71 %). TLC *R*_f_ = 0.47 (5 % EtOAc in hexane). IR (thin film): ν̃ = 2956, 1249, 833, 747 cm^–1^. ^1^H NMR (500 MHz, CDCl_3_): *δ* = (diastereomeric ratio 60:40) 5.97–5.75 (m, 2 H, 2′′‐H and 4′‐H), 5.16–4.92 (m, 4 H, 5′‐H and 3′′‐H), 4.03 (dd, *J* = 5.0, 2.0 Hz, 0.6 H, 3‐H), 3.94 (dd, *J* = 5.0, 2.0 Hz, 0.4 H, 3‐H), 3.89–3.81 (m, 1.4 H, 2‐H and 5‐H), 3.75 (dd, *J* = 3.5, 2.0 Hz, 0.6 H, 2‐H), 3.70–3.64 (m, 1 H, 1′‐H), 3.62 (dd, *J* = 8.0, 5.0 Hz, 0.4 H, 4‐H), 3.56 (dd, *J* = 8.0, 5.0 Hz, 0.6 H, 4‐H), 2.48–2.40 (m, 1 H, 1′′‐H), 2.21–2.01 (m, 3 H, 1′′‐H and 3′‐H), 1.75 (ddt, *J* = 13.0, 9.5, 6.0 Hz, 0.4 H, 2′‐H), 1.65–1.50 (m, 1.2 H, 2′‐H), 1.42 (dddd, *J* = 13.5, 7.0, 6.5, 6.5 Hz, 0.4 H, 2′‐H), 0.16 (s, 6 H, CH_3_), 0.15 (s, 3 H, CH_3_), 0.14 (s, 10 H, CH_3_), 0.13 (s, 8 H, CH_3_) ppm. ^13^C NMR (126 MHz, CDCl_3_): *δ* = 138.6, 138.4, 135.4, 135.2, 116.9, 116.8, 114.84, 114.80, 88.3, 87.3, 79.8, 79.4, 76.1, 75.7, 73.9, 72.62, 72.57, 72.1, 37.4, 37.0, 33.7, 32.4, 30.2, 29.9, 0.9, 0.8, 0.8, 0.6, 0.5 ppm. LC‐MS (ESI^+^): *m/z* 467 [M + Na]^+^. HRMS (ESI^+^): [M + Na^+^] calcd. for C_21_H_44_O_4_Si_3_Na 467.2440, found 467.2429.

**{[(1*S*)‐ and (1*R*)‐1‐[(3a*S*,4*R*,6*R*,6a*R*)‐2,2‐Dimethyl‐6‐(prop‐2‐en‐1‐yl)‐tetrahydro‐2*H*‐furo[3,4‐*d*][1,3]dioxol‐4‐yl]pent‐4‐en‐1‐yl]oxy}(*tert*‐butyl)dimethylsilane (1g):** *tert*‐Butyldimethylsilyl trifluoromethanesulfonate (0.083 mL, 0.36 mmol) was added to a stirred solution of a 60:40 epimeric mixture of (1*S*)‐ and (1*R*)‐1‐[(3a*S*,4*S*,6*R*,6a*R*)‐2,2‐dimethyl‐6‐(prop‐2‐en‐1‐yl)‐tetrahydro‐2*H*‐furo[3,4‐*d*][1,3]dioxol‐4‐yl]pent‐4‐en‐1‐ol (**9**) (0.040 g, 0.15 mmol) and DIPEA (0.11 mL, 0.60 mmol) in dry CH_2_Cl_2_ (1.5 mL) under a N_2_ atmosphere at room temp. After 1 h the reaction was quenched with NH_4_Cl solution (satd. aq., 5 mL) and extracted with EtOAc (3 × 5 mL). The combined organic layers were dried (MgSO_4_), filtered and the solvents evaporated under vacuum. The residue was purified by column chromatography (14 % EtOAc in hexane) to afford a 67:33 epimeric mixture of **1g** as a colourless oil (0.058 g, 100 %). TLC *R*_f_ = 0.75 (14 % EtOAc in hexane). IR (thin film): ν̃ = 3079, 2929, 2857, 1734, 1643, 1079 cm^–1^. ^1^H NMR (500 MHz, CDCl_3_): *δ* = (diastereomeric ratio 67:33) 5.90–5.76 (m, 2 H, 4′‐H and 2′′‐H), 5.18–5.07 (m, 2 H, 3′′‐H), 5.03 (dq, *J* = 17.0, 2.0 Hz, 1 H, 5′‐H), 4.99–4.94 (m, 1 H, 5′‐H), 4.72 (dd, *J* = 7.0, 4.5 Hz, 0.67 H, 3a‐H), 4.48 (dd, *J* = 7.0, 4.0 Hz, 0.33 H, 3a‐H), 4.30 (dd, *J* = 7.0, 5.0 Hz, 0.33 H, 6a‐H), 4.21 (dd, *J* = 7.0, 6.0 Hz, 0.67 H, 6a‐H), 3.94–3.89 (m, 0.66 H, 4‐H and 6‐H), 3.85 (td, *J* = 6.5, 2.5 Hz, 0.67 H, 1′‐H), 3.81–3.75 (m, 1.34 H, 4‐H and 6‐H), 3.70 (td, *J* = 7.0, 5.0 Hz, 0.33 H, 1′‐H), 2.41–2.35 (m, 2 H, 1′′‐H), 2.23–2.06 (m, 2 H, 3′‐H), 1.77–1.69 (m, 0.33 H, 2′‐H), 1.64–1.58 (m, 1.67 H, 2′‐H), 1.52 [s, 3 H, C(C*H*_3_)_2_], 1.34 [s, 2 H, C(C*H*_3_)_2_], 1.32 [s, 1 H, C(C*H*_3_)_2_], 0.90 [s, 3 H, SiC(C*H*_3_)_3_], 0.90 [s, 6 H, SiC(C*H*_3_)_3_], 0.07 [apt. dd, *J* = 9.8, 4.9 Hz, 6 H, Si(CH_3_)_2_] ppm. ^13^C NMR (126 MHz, CDCl_3_): *δ* = 138.7, 138.5, 134.3, 134.2, 117.6, 117.5, 114.84, 114.83, 114.79, 86.5, 85.9, 84.4, 84.2, 83.9, 83.4, 82.4, 80.0, 72.7, 71.1, 38.0, 37.9, 33.8, 32.4, 29.9, 29.8, 27.7, 27.6, 26.1, 25.7, 18.4, 18.3, –3.9, –4.1, –4.3 ppm. HRMS (ESI^+^): [M + H^+^] calcd. for C_21_H_39_O_4_Si 383.2612, found 383.2605.

**(1*R*)‐ and (1*S*)‐({1‐[(3a*S*,4*R*,6*R*,6a*R*)‐2,2‐Dimethyl‐6‐(prop‐2‐en‐1‐yl)‐tetrahydro‐2*H*‐furo[3,4‐*d*][1,3]dioxol‐4‐yl]‐pent‐4‐en‐1‐yl}oxy)(*tert*‐butyl)diphenylsilane (1h):** *tert*‐Butyldiphenylsilyl trifluoromethanesulfonate (0.46 mL, 1.5 mmol) and 2,6‐lutidine (0.17 mL, 1.5 mmol) were added to a stirred solution of (1*S*)‐ and (1*R*)‐1‐[(3a*S*,4*S*,6*R*,6a*R*)‐2,2‐dimethyl‐6‐(prop‐2‐en‐1‐yl)‐tetrahydro‐2*H*‐furo[3,4‐*d*][1,3]dioxol‐4‐yl]pent‐4‐en‐1‐ol (**9**) (0.20 g, 0.75 mmol) in CH_2_Cl_2_ (2.5 mL) under a N_2_ atmosphere in a sealed vessel. The solution was heated at 40 °C for 96 h then cooled and water (10 mL) was added. The mixture was extracted with CH_2_Cl_2_ (3 × 10 mL) and the combined organic layers were dried (Na_2_SO_4_), filtered and the solvents evaporated under vacuum. The residue was purified by column chromatography (5 % EtOAc in hexane) to afford a 60:40 epimeric mixture of **1h** as a colourless oil (0.31 g, 80 %). TLC *R*_f_ 0.47 (10 % EtOAc in hexane). IR (thin film): ν̃ = 3073, 2932, 2857, 1111, 1080, 702 cm^–1^. ^1^H NMR (500 MHz, CDCl_3_): *δ* = (diastereomeric ratio 60:40) 7.73–7.69 (m, 4 H, ArH), 7.45–7.33 (m, 6 H, ArH), 5.87–5.69 (m, 1 H, 2′′‐H), 5.57–5.43 (m, 1 H, 4′‐H), 5.15–5.03 (m, 2 H, 3′′‐H), 4.84 (dd, *J* = 7.0, 4.5 Hz, 0.6 H, 3a‐H), 4.82–4.77 (m, 2 H, 5′‐H), 4.55 (dd, *J* = 7.0, 4.0 Hz, 0.4 H, 3a‐H), 4.30 (dd, *J* = 7.0, 5.0 Hz, 0.4 H, 6a‐H), 4.20 (dd, *J* = 7.0, 5.5 Hz, 0.6 H, 6a‐H), 3.94 (t, *J* = 4.5 Hz, 0.4 H, 4‐H), 3.92–3.87 (m, 0.6 H, 1′‐H), 3.85–3.80 (m, 1 H, 4‐H and 6‐H), 3.80–3.74 (m, 1 H, 1′‐H and 6‐H), 2.39–2.33 (m, 0.8 H, 1′′‐H), 2.28–2.18 (m, 1.2 H, 1′′‐H), 2.01–1.86 (m, 2 H, 3′‐H), 1.74–1.66 (m, 0.4 H, 2′‐H), 1.60–1.48 [m, 4 H, 2′‐H and C(C*H*_3_)_2_], 1.48–1.40 (m, 0.6 H, 2′‐H), 1.34 [s, 1.8 H, C(C*H*_3_)_2_], 1.31 [s, 1.2 H, C(C*H*_3_)_2_], 1.07 [s, 5.2 H, SiC(C*H*_3_)_3_], 1.05 [s, 3.8 H, SiC(C*H*_3_)_3_] ppm. ^13^C NMR (126 MHz, CDCl_3_): *δ* = 138.3, 138.2, 136.3, 136.23, 136.17, 134.5, 134.4, 134.3, 134.1, 133.92, 133.88, 129.8, 129.71, 129.69, 129.6, 127.64, 127.57, 127.56, 127.5, 117.50, 117.45, 114.68, 114.65 and 114.63, 114.2, 86.1, 85.4, 84.4, 84.1, 83.60, 83.59, 82.4, 80.5, 73.7, 72.2, 37.8, 33.4, 32.3, 29.6, 29.2, 27.64, 27.61, 27.3, 27.2, 25.72, 25.68, 19.7, 19.6 ppm. HPLC (Method B): *t*_R_ = 1.51 min; purity (AUC) ≥ 95 %. HRMS (ESI^+^): [M + Na^+^] calcd. for C_31_H_42_O_4_SiNa 383.2605, found 383.2612.

**(1*R*)‐ and (1*S*)‐{[1‐[(2*R*,3*R*,4*R*,5*R*)‐3,4‐Bis(benzyloxy)‐5‐(prop‐2‐en‐1‐yl)oxolan‐2‐yl]pent‐4‐en‐1‐yl]oxy}(*tert*‐butyl)diphenylsilane (1i):** Ethanedithiol (0.12 mL, 1.4 mmol) and *p*‐toluenesulfonic acid (0.002 g, 0.010 mmol) were added to a stirred solution of (1*R*)‐ and (1*S*)‐({1‐[(3a*S*,4*R*,6*R*,6a*R*)‐2,2‐dimethyl‐6‐(prop‐2‐en‐1‐yl)‐tetrahydro‐2*H*‐furo[3,4‐*d*][1,3]dioxol‐4‐yl]‐pent‐4‐en‐1‐yl}oxy)(*tert*‐butyl)diphenylsilane (**1h**) (0.10 g, 0.20 mmol) in CHCl_3_ (4.0 mL) under a N_2_ atmosphere. The solution was heated to 60 °C for 18 h then cooled. NaHCO_3_ solution (satd. aq., 10 mL) was added and the mixture was extracted with CH_2_Cl_2_ (3 × 10 mL). the combined organic layers were dried (Na_2_SO_4_), filtered and the solvents evaporated under vacuum. The residue was purified by column chromatography (40 % EtOAc in hexane) to afford a 60:40 epimeric mixture of (1*R*)‐ and (1*S*)‐(2*R*,3*R*,4*S*,5*R*)‐2‐{1‐[(*tert*‐butyldiphenylsilyl)oxy]pent‐4‐en‐1‐yl}‐5‐(prop‐2‐en‐1‐yl)oxolane‐3,4‐diol as a colourless oil (0.060 g, 64 %). TLC *R*_f_ 0.47 (33 % EtOAc in hexane). IR (thin film): ν̃ = 3385, 3071, 2930, 2857, 1110, 703 cm^–1^. ^1^H NMR (500 MHz, CDCl_3_): *δ* = (diastereomeric ratio 60:40) 7.74–7.68 (m, 4 H, ArH), 7.46–7.41 (m, 2 H, ArH), 7.41–7.35 (m, 4 H, ArH), 5.91–5.79 (m, 1 H, 2′′‐H), 5.56–5.42 (m, 1 H, 4′‐H), 5.16–5.05 (m, 2 H, 3′′‐H), 4.83–4.75 (m, 2 H, 5′‐H), 4.22 (dt, *J* = 6.0, 4.5 Hz, 0.6 H, 3‐H), 4.10 (dt, *J* = 6.0, 4.5 Hz, 0.4 H, 3‐H), 3.93–3.87 (m, 1 H, 1′‐H), 3.82–3.76 (m, 0.8 H, 2‐H and 4‐H), 3.76–3.69 (m, 1 H, 2‐H and 5‐H), 3.69–3.62 (m, 1.2 H, 4‐H and 5‐H), 2.42–2.33 (m, 2.4 H, 1′′‐H and 2 × OH), 2.33–2.25 (m, 1.4 H, 1′′‐H and OH), 2.10 (d, *J* = 4.5 Hz, 0.4 H, OH), 2.01–1.83 (m, 2 H, 3′‐H), 1.79–1.70 (m, 0.4 H, 2′‐H), 1.60–1.39 (m, 1.6 H, 2′‐H), 1.07 (s, 5 H, CH_3_), 1.06 (s, 4 H, CH_3_) ppm. ^13^C NMR (126 MHz, CDCl_3_): *δ* = 138.3, 138.2, 136.2, 136.10, 136.09, 136.07, 134.5, 134.31, 134.29, 134.26, 133.8, 133.7, 123.0, 129.89, 129.87, 129.84, 127.81, 127.75, 127.71, 117.6, 117.5, 114.7, 114.6, 86.6, 84.9, 82.1, 81.8, 74.8, 74.6, 73.3, 73.0, 71.5, 71.0, 37.7, 37.6, 33.4, 32.1, 30.0, 29.2, 27.3, 27.2, 19.7, 19.6 ppm. LC‐MS (ESI^+^): *m/z* 489 [M + Na^+^]. HRMS (ESI^+^): [M + Na^+^] calcd. for C_28_H_38_O_4_SiNa 489.2432, found 489.2419. HPLC (Method B): *t*_R_ = 1.26 min; purity (AUC) ≥ 95 %. NaH (60 % in oil, 0.029 g, 0.73 mmol) was added to a stirred solution of (1*R*)‐ and (1*S*)‐(2*R*,3*R*,4*S*,5*R*)‐2‐{1‐[(*tert*‐butyldiphenylsilyl)oxy]pent‐4‐en‐1‐yl}‐5‐(prop‐2‐en‐1‐yl)oxolane‐3,4‐diol (0.085 g, 0.18 mmol) in DMF (1.8 mL) at 0 °C under a N_2_ atmosphere. After 5 min at 0 °C benzyl bromide (0.090 mL, 0.73 mmol) was added and the solution was warmed to room temp. After 5 h NaHCO_3_ solution (satd. aq., 5 mL) and water (5 mL) were added at 0 °C and the mixture was extracted with Et_2_O (3 × 10 mL). The combined organic layers were dried (Na_2_SO_4_), filtered and the solvents evaporated under vacuum. The residue was purified by column chromatography (5 % EtOAc in hexane) to afford a 60:40 epimeric mixture of **1i** as a yellow oil (0.076 g, 65 %). TLC *R*_f_ 0.33 (10 % EtOAc in hexane). IR (thin film): ν̃ = 3071, 2930, 2857, 1111, 701 cm^–1^. ^1^H NMR (500 MHz, CDCl_3_): *δ* = (diastereomeric ratio 60:40) 7.75–7.70 (m, 2 H, ArH), 7.67 (ddd, *J* = 8.1, 5.4, 1.4 Hz, 2 H, ArH), 7.46–7.40 (m, 2 H, ArH), 7.39–7.27 (m, 14 H, ArH), 5.88 (dddt, *J* = 17.0, 14.0, 10.0, 7.0 Hz, 1 H, 2′′‐H), 5.41 (ddt, *J* = 17.0, 10.0, 6.5 Hz, 0.6 H, 4′‐H), 5.37–5.28 (m, 0.4 H, 4′‐H), 5.12–5.03 (m, 2 H, 3′′‐H), 4.80–4.69 (m, 2 H, 5′‐H), 4.60–4.43 (m, 4 H, CH_2_), 4.10–4.03 (m, 1.8 H, 2‐H, 4‐H and 5‐H), 4.03–3.97 (m, 1.2 H, 2‐H and 5‐H), 3.89–3.85 (m, 0.6 H, 1′‐H), 3.76 (dt, *J* = 8.0, 4.5, 3.0 Hz, 0.4 H, 1′‐H), 3.71 (dd, *J* = 6.5, 5.5 Hz, 0.4 H, 3‐H), 3.51 (dd, *J* = 8.0, 5.5 Hz, 0.6 H, 3‐H), 2.49–2.39 (m, 1 H, 1′′‐H), 2.37–2.29 (m, 0.4 H, 1′′‐H), 2.26–2.18 (m, 0.6 H, 1′′‐H), 1.96–1.71 (m, 2.4 H, 2′‐H and 3′‐H), 1.48–1.37 (m, 1 H, 2′‐H), 1.37–1.23 (m, 0.6 H, 2′‐H), 1.04 (s, 5 H, CH_3_), 0.99 (s, 4 H, CH_3_) ppm. ^13^C NMR (126 MHz, CDCl_3_): *δ* = 138.3, 138.21, 138.20, 138.16, 138.07, 138.06 (4′), 136.2, 136.09, 136.06, 136.0, 134.9, 134.7, 134.6, 134.5, 133.7, 133.6, 129.85, 129.82, 129.79, 129.6, 128.54, 128.50, 128.47, 128.46, 128.2, 128.1, 127.97, 127.95, 127.88, 127.83, 127.73, 127.67, 127.5, 117.2, 117.1, 114.6, 114.5, 85.2, 83.7, 81.7, 80.8, 79.8, 79.6, 77.5, 76.3, 73.3, 73.1, 72.6, 72.2, 72.1, 72.1, 37.8, 37.7, 33.0, 32.3, 29.8, 29.4, 27.3, 27.2, 19.6, 19.6 ppm. LC‐MS (ESI^+^): *m/z* 669 [M + Na^+^]. HRMS (ESI^+^): [M + Na^+^] calcd. for C_42_H_50_O_4_SiNa 669.3371, found 669.3383. HPLC (Method B): *t*_R_ = 1.56 min; purity (AUC) ≥ 95 %.

**(1*R*)‐ and (1*S*)‐{[1‐[(3a*S*,4*S*,6*R*,6a*R*)‐2,2‐Di‐*tert*‐butyl‐6‐(prop‐2‐en‐1‐yl)‐tetrahydro‐2*H*‐furo[3,4‐*d*][1,3,2]dioxasilol‐4‐yl]pent‐4‐en‐1‐yl]oxy}(*tert*‐butyl)diphenylsilane (1j):** (1*R*)‐ and (1*S*)‐(2*R*,3*R*,4*S*,5*R*)‐2‐{1‐[(*tert*‐Butyldiphenylsilyl)oxy]pent‐4‐en‐1‐yl}‐5‐(prop‐2‐en‐1‐yl)oxolane‐3,4‐diol was prepared as for **1i**. Di‐*tert*‐butylsilyl bis(trifluoromethanesulfonate) (0.24 mL, 0.75 mmol) and 2,6‐lutidine (0.17 mL, 1.5 mmol) were added to a stirred solution of (1*R*)‐ and (1*S*)‐(2*R*,3*R*,4*S*,5*R*)‐2‐{1‐[(*tert*‐butyldiphenylsilyl)oxy]pent‐4‐en‐1‐yl}‐5‐(prop‐2‐en‐1‐yl)oxolane‐3,4‐diol (0.18 g, 0.38 mmol) in CH_2_Cl_2_ (3.8 mL) under a N_2_ atmosphere in a sealed vessel. The solution was heated at 40 °C for 48 h then cooled and poured into NaHCO_3_ solution (satd. aq., 10 mL). The mixture was extracted with CH_2_Cl_2_ (3 × 10 mL) and the combined organic layers were dried (Na_2_SO_4_), filtered and the solvents evaporated under vacuum. The residue was purified by column chromatography (5 % EtOAc in hexane) to afford a 60:40 epimeric mixture of **1j** as a colourless oil (0.16 g, 68 %). TLC *R*_f_ 0.41 (5 % EtOAc in hexane). IR (thin film): ν̃ = 3073, 2932, 2859, 1077, 822, 702 cm^–1^. ^1^H NMR (500 MHz, CDCl_3_): *δ* = (diastereomeric ratio 60:40) 7.75–7.68 (m, 4 H, ArH), 7.45–7.31 (m, 6 H, ArH), 5.85 (ddt, *J* = 17.0, 10.5, 7.0 Hz, 0.6 H, 2′′‐H), 5.75–5.51 (m, 1.4 H, 2′′‐H and 4′‐H), 5.12–5.05 (m, 1.6 H, 3′′‐H), 5.03–4.99 (m, 0.4 H, 3′′‐H), 4.88–4.77 (m, 2 H, 5′‐H), 4.67 (dd, *J* = 8.0, 6.0 Hz, 0.6 H, 3a‐H), 4.30 (dd, *J* = 8.0, 6.0 Hz, 0.4 H, 3a‐H), 4.10–4.04 (m, 1 H, 6a‐H), 4.01–3.96 (m, 0.6 H, 1′‐H), 3.85 (q, *J* = 5.5 Hz, 0.4 H, 1′‐H), 3.70 (t, *J* = 6.0 Hz, 0.4 H, 4‐H), 3.68–3.61 (m, 1.2 H, 4‐H and 6‐H), 3.61–3.54 (m, 0.4 H, 6‐H), 2.46–2.36 (m, 1 H, 1′′‐H), 2.32–2.24 (m, 0.4 H, 1′′‐H), 2.19–2.11 (m, 0.6 H, 1′′‐H), 2.10–1.94 (m, 2 H, 3′‐H), 1.73–1.48 (m, 2 H, 2′‐H), 1.08–1.02 (m, 24 H, CH_3_), 0.99 (s, 3 H, CH_3_) ppm. ^13^C NMR (126 MHz, CDCl_3_): *δ* = 138.8, 138.5, 136.32, 136.29, 136.19, 136.15, 134.9, 134.7, 134.51, 134.50, 134.2, 129.7, 129.6, 129.51, 129.47, 127.6, 127.49, 127.47, 127.4, 117.1, 117.0, 114.5, 114.4, 86.8, 86.7, 84.2, 83.7, 82.3, 81.7, 79.2, 77.4, 73.7, 72.3, 37.7, 37.5, 33.2, 32.5, 29.6, 29.5, 27.71, 27.70, 27.3, 27.2, 26.9, 26.9, 22.10, 22.06, 19.81, 19.80, 19.7, 19.6 ppm. HRMS (ESI^+^): [M + Na^+^] calcd. for C_36_H_54_O_4_Si_2_Na 629.3453, found 629.3437. HPLC (Method B): *t*_R_ = 1.88 min; purity (AUC) ≥ 95 %.

**(1*R*,3*Z*,7*S*,8*S*,9*S*,13*R*)‐11,11‐Dimethyl‐7‐(naphthalen‐2‐ylmethoxy)‐10,12,14‐trioxatricyclo[6.5.1.0^9,13^]tetradec‐3‐ene (10a):** A solution of dichloro[1,3‐bis(2‐methylphenyl)‐2‐imidazolidinylidene](2‐isopropoxyphenylmethylene)ruthenium(II) (0.014 g, 0.024 mmol) in toluene (20 mL) was added to a stirred solution of a 60:40 epimeric mixture of (1*S*)‐ and (1*R*)‐(3a*S*,4*S*,6*R*,6a*R*)‐2,2‐dimethyl‐4‐[1‐(naphthalen‐2‐ylmethoxy)pent‐4‐en‐1‐yl]‐6‐(prop‐2‐en‐1‐yl)‐tetrahydro‐2*H*‐furo[3,4‐*d*][1,3]dioxole (**1a**) (0.10 g, 0.24 mmol) in toluene (62 mL) under a N_2_ atmosphere. The mixture was stirred at 110 °C for 48 h then cooled to room temp. The mixture was passed through an Isolute NH_2_ basic ion exchange cartridge (5 g), eluting with toluene (approx. 150 mL). The eluent was evaporated under vacuum and the residue was purified by column chromatography (10 % EtOAc in hexane), then by preparative TLC (100 % CH_2_Cl_2_). Further purification by preparative TLC (10 % EtOAc in hexane) afforded **10a** as a white solid (0.002 g, 2 %). TLC *R*_f_ = 0.48 (15 % EtOAc in hexane). IR (thin film): ν̃ = 2924, 2855, 1073 cm^–1^. ^1^H NMR (500 MHz, CDCl_3_): *δ* = 7.85–7.81 (m, 3 H, ArH), 7.78 (s, 1 H, ArH), 7.50–7.45 (m, 3 H, ArH), 5.77 (dddd, *J* = 11.0, 11.0, 6.0, 1.5 Hz, 1 H, 4‐H), 5.59 (ddd, *J* = 11.0, 9.0, 6.5 Hz, 1 H, 3‐H), 4.79 (d, *J* = 12.0 Hz, 1 H, OCH_2_), 4.55 (d, *J* = 12.0 Hz, 1 H, OCH_2_), 4.36–4.33 (m, 2 H, 9‐H and 13‐H), 4.22–4.19 (m, 1 H, 1‐H), 4.09 (d, *J* = 9.5 Hz, 1 H, 8‐H), 3.17 (td, *J* = 9.5, 2.5 Hz, 1 H, 7‐H), 2.81–2.71 (m, 1 H, 5‐H), 2.50–2.43 (m, 1 H, 2‐H), 2.08–1.92 (m, 3 H, 2‐H, 5‐H and 6‐H), 1.88–1.80 (m, 1 H, 6‐H), 1.49 (s, 3 H, CH_3_), 1.23 (s, 3 H, CH_3_) ppm. ^13^C NMR (126 MHz, CDCl_3_): *δ* = 135.8, 134.5, 133.4, 133.1, 128.3, 128.0, 127.8, 126.8, 126.3, 126.1 (2 C), 124.9, 112.7, 86.3, 86.2, 85.6, 82.9, 79.7, 71.0, 30.9, 28.8, 27.4, 25.5, 25.1 ppm. LC‐MS (ESI^+^): *m/z* 381 [M + H^+^]. HRMS (ESI^+^): [M + H^+^] calcd. for C_24_H_29_O_4_ 381.2050, found 381.2060.

**(7*R*)‐ and (7*S*)‐{[(1*R*,3*Z*,8*S*,9*S*,10*R*)‐7,10‐Bis[(*tert*‐butyldiphenylsilyl)oxy]‐11‐oxabicyclo[6.2.1]undec‐3‐en‐9‐yl]oxy}(*tert*‐butyl)diphenylsilane (10d):** Dichloro[1,3‐bis(2‐methylphenyl)‐2‐imidazolidinylidene](2‐isopropoxyphenylmethylene)ruthenium(II) (0.006 g, 0.011 mmol) was added to a stirred solution of (1*R*)‐ and (1*S*)‐*tert*‐butyl({[(2*R*,3*R*,4*S*,5*S*)‐4‐[(*tert*‐butyldiphenylsilyl)oxy]‐5‐[1‐[(*tert*‐butyldiphenylsilyl)oxy]pent‐4‐en‐1‐yl]‐2‐(prop‐2‐en‐1‐yl)oxolan‐3‐yl]oxy})diphenylsilane (**1d**) (0.10 g, 0.11 mmol) in toluene (35 mL) under a N_2_ atmosphere and heated at 110 °C for 24 h. The solution was cooled and filtered through an Isolute NH_2_ basic ion exchange cartridge (5 g), eluting with toluene (150 mL). The eluent was evaporated under vacuum and the residue was purified by column chromatography (5 % Et_2_O in hexane) to afford a 70:30 epimeric mixture of **10d** as a colourless gum (0.054 g, 49 %). TLC *R*_f_ = 0.44 (5 % Et_2_O in hexane). IR (thin film): ν̃ = 3071, 2930, 2857, 1111, 701 cm^–1^. ^1^H NMR (500 MHz, [D_8_]toluene, 355 K): *δ* = (diastereomeric ratio 70:30) 8.00–7.91 (m, 4 H, ArH), 7.88–7.74 (m, 3 H, ArH), 7.70–7.65 (m, 0.7 H, ArH), 7.58–7.48 (m, 3 H, ArH), 7.34–7.04 (m, 18 H, ArH), 5.27 (tdd, *J* = 11.0, 6.0, 2.0 Hz, 0.3 H, 4‐H), 5.00 (dd, *J* = 8.0, 4.5 Hz, 0.3 H, 9‐H), 4.91–4.81 (m, 1.3 H, 3‐H, 9‐H and 10‐H), 4.72 (tdd, *J* = 11.5, 6.0, 2.0 Hz, 0.7 H, 4‐H), 4.39 (dt, *J* = 7.0, 3.0 Hz, 0.7 H, 1‐H), 4.37–4.33 (m, 0.3 H, 7‐H), 4.30–4.23 (m, 0.7 H, 3‐H), 4.21 (dd, *J* = 7.0, 3.5 Hz, 0.7 H, 10‐H), 4.06 (d, *J* = 9.0 Hz, 0.7 H, 8‐H), 3.93 (t, *J* = 3.5 Hz, 0.3 H, 1‐H), 3.69 (d, *J* = 4.5 Hz, 0.3 H, 8‐H), 3.35–3.29 (m, 0.7 H, 7‐H), 2.72 (qd, *J* = 12.0, 6.0 Hz, 0.7 H, 5‐H), 2.30–2.22 (m, 0.7 H, 2‐H), 2.22–2.15 (m, 0.3 H, 5‐H), 1.88–1.80 (m, 0.3 H, 6‐H), 1.78–1.71 (m, 0.3 H, 2‐H), 1.68–1.60 (m, 0.3 H, 6‐H), 1.57–1.47 (m, 1 H, 2‐H and 5‐H), 1.46–1.38 (m, 0.7 H, 6‐H), 1.37–1.10 (m, 22.4 H, 5‐H, 6‐H and CH_3_), 1.00–0.91 (m, 6 H, CH_3_), 0.91–0.78 (m, 0.3 H, 2‐H) ppm. ^13^C NMR (126 MHz, [D_8_]toluene, 355 K): *δ* = 137.7, 137.5, 137.2, 136.9, 136.81, 136.79, 136.76, 136.74, 136.71, 136.54, 136.46, 136.42, 136.37, 136.3, 134.11, 130.3, 130.13, 130.10, 130.07, 130.0, 129.91, 129.90, 129.83, 129.78, 129.76, 129.1, 129.0, 128.8, 128.26, 128.25, 128.19, 128.16, 128.1, 128.02, 127.99, 127.92, 127.87, 127.83, 127.82, 125.2, 124.4, 87.6, 85.3, 84.3, 82.2, 78.9, 78.3, 76.1, 75.5, 75.0, 72.7, 34.8, 32.1, 30.2, 28.39, 28.37, 28.0, 27.9, 27.7, 27.6, 27.5, 25.6, 23.2 ppm, [*C*(CH_3_)_3_ peaks not assigned as obscured by solvent]. LC‐MS (ESI^+^): *m/z* 937 [M + Na^+^]. HRMS (ESI^+^): [M + Na^+^] calcd. for C_58_H_70_O_4_Si_3_Na 937.4474, found 937.4449. HPLC (Method B): *t*_R_ = 2.63 min; purity (AUC) ≥ 95 %.

**{[(1*S*,2*S*,5*Z*,8*R*,9*R*,10*S*)‐2,10‐Bis[(*tert*‐butyldimethylsilyl)oxy]‐11‐oxabicyclo[6.2.1]undec‐5‐en‐9‐yl]oxy}(*tert*‐butyl)dimethylsilane (10e):** Dichloro[1,3‐bis(2‐methylphenyl)‐2‐imidazolidinylidene](2‐isopropoxyphenylmethylene)ruthenium(II) (0.050 g, 0.088 mmol) was added to a stirred solution of *tert*‐butyl({[(2*R*,3*R*,4*S*,5*S*)‐4‐[(*tert*‐butyldimethylsilyl)oxy]‐5‐[(1*R*)‐ and (1*S*)‐1‐[(*tert*‐butyldimethylsilyl)oxy]pent‐4‐en‐1‐yl]‐2‐(prop‐2‐en‐1‐yl)oxolan‐3‐yl]oxy})dimethylsilane (**1e**) (0.50 g, 0.88 mmol) in toluene (290 mL) under a N_2_ atmosphere and heated at 110 °C for 24 h. The solution was cooled and filtered through an Isolute SCX‐2 acidic ion exchange cartridge (20 g), eluting with toluene (200 mL). The filtrate was evaporated under vacuum. A sample (0.083 g) of the crude product (0.43 g) was purified by column chromatography (2 % Et_2_O in hexane) and then by preparative TLC (2 % Et_2_O in cyclohexane) to afford **10e** as a white gum (0.015 g). TLC *R*_f_ = 0.39 (5 % Et_2_O in hexane). IR (thin film): ν̃ = 2929, 2857, 834, 772 cm^–1^. ^1^H NMR (500 MHz, CDCl_3_): *δ* = 5.80–5.72 (m, 1 H, 5‐H), 5.68–5.60 (m, 1 H, 6‐H), 4.07–4.01 (m, 2 H, 8‐H and 9‐H), 3.93 (d, *J* = 3.0 Hz, 1 H, 10‐H), 3.63 (d, *J* = 9.5 Hz, 1 H, 1‐H), 3.33–3.26 (m, 1 H, 2‐H), 2.94 (qd, *J* = 12.0, 7.0 Hz, 1 H, 4‐H), 2.67–2.59 (m, 1 H, 7‐H), 2.10 (ddd, *J* = 15.0, 8.5, 2.0 Hz, 1 H, 7‐H), 1.96–1.89 (m, 1 H, 4‐H), 1.81–1.73 (m, 1 H, 3‐H), 1.73–1.64 (m, 1 H, 3‐H), 0.92–0.87 [m, 27 H, C(CH_3_)_3_], 0.11–0.02 [m, 18 H, Si(CH_3_)_2_] ppm. ^13^C NMR (126 MHz, CDCl_3_): *δ* = 134.5, 125.6, 89.4, 81.4, 75.7, 73.5, 71.0, 35.0, 27.8, 26.10, 26.07, 26.0, 18.3, 18.2, 18.0, –3.9, –4.05, –4.14, –4.4, –4.7 ppm. LC‐MS (ESI^+^): *m/z* 565 [M + Na^+^]. HRMS (ESI^+^): [M + Na^+^] calcd. for C_28_H_58_O_4_Si_3_Na 543.3716, found 543.3711.

**(1*R*,3*Z*,7*S*,8*S*,9*S*,13*R*)‐11,11‐Di‐*tert*‐butyl‐7‐[(*tert*‐butyldiphenylsilyl)oxy]‐10,12,14‐trioxa‐11‐silatricyclo[6.5.1.0^9,13^]tetradec‐3‐ene (10j):** Dichloro[1,3‐bis(2‐methylphenyl)‐2‐imidazolidinylidene](2‐isopropoxyphenylmethylene)ruthenium(II) (0.009 g, 0.016 mmol) was added to a stirred solution of (1*R*)‐ and (1*S*)‐{[1‐[(3a*S*,4*S*,6*R*,6a*R*)‐2,2‐di‐*tert*‐butyl‐6‐(prop‐2‐en‐1‐yl)‐tetrahydro‐2*H*‐furo[3,4‐*d*][1,3,2]dioxasilol‐4‐yl]pent‐4‐en‐1‐yl]oxy}(*tert*‐butyl)diphenylsilane (**1j**) (0.10 g, 0.16 mmol) in toluene (55 mL) under a N_2_ atmosphere and heated at 110 °C for 18 h. The solution was cooled and filtered through an Isolute NH_2_ basic ion exchange cartridge (5 g), eluting with toluene (100 mL). The eluent was evaporated under vacuum and the residue was purified by column chromatography (5 % Et_2_O in hexane). Further purification by column chromatography (1 % Et_2_O in CH_2_Cl_2_) gave **10j** as a colourless gum (0.002 g, 2 %). [*α*]_D_^22^ = –33 (*c* = 0.5, CHCl_3_). TLC *R*_f_ = 0.32 (1 % Et_2_O in CH_2_Cl_2_). IR (thin film): ν̃ = 2927, 2587, 1472, 1056, 825, 703 cm^–1^. ^1^H NMR (500 MHz, CDCl_3_): *δ* = 7.76–7.60 (m, 5 H, ArH), 7.48–7.28 (m, 5 H, ArH), 5.32–5.24 (m, 1 H, 3‐H), 5.24–5.15 (m, 1 H, 4‐H), 4.28 (d, *J* = 6.0 Hz, 1 H, 9‐H), 4.16–4.12 (m, 1 H, 1‐H), 4.12–4.06 (m, 2 H, 8‐H and 13‐H), 3.17–3.11 (m, 1 H, 7‐H), 2.55–2.41 (m, 1 H, 5‐H), 2.41–2.33 (m, 1 H, 2‐H), 1.91 (ddd, *J* = 14.0, 8.5, 5.0 Hz, 1 H, 2‐H), 1.87–1.77 (m, 1 H, 6‐H), 1.66–1.57 (m, 2 H, 5‐H and 6‐H), 1.13–0.77 (m, 27 H, CH_3_) ppm. ^13^C NMR (126 MHz, CDCl_3_): *δ* = 136.0, 134.1, 134.0, 133.7, 129.7, 127.6, 124.0, 90.2, 87.0, 84.2, 81.4, 73.9 (C_7_ assigned from HSQCed as not visible in ^13^C), 33.2, 30.6, 27.6, 27.5, 27.2, 27.0, 26.9, 26.8, 26.7, 25.0, 21.7, 19.9, 19.1 ppm. LC‐MS (ESI^+^): *m/z* 601 [M + Na^+^]. HRMS (ESI^+^): [M + Na^+^] calcd. for C_34_H_50_O_4_Si_2_Na 601.3140, found 601.3137. HPLC (Method B): *t*_R_ = 2.77 min; purity (AUC) ≥ 95 %.

**(1*S*,2*S*,5*Z*,8*R*,9*S*,10*R*)‐11‐Oxabicyclo[6.2.1]undec‐5‐ene‐2,9,10‐triol (2):** Dichloro[1,3‐bis(2‐methylphenyl)‐2‐imidazolidinylidene](2‐isopropoxyphenylmethylene)ruthenium(II) (0.050 g, 0.088 mmol) was added to a stirred solution of *tert*‐butyl({[(2*R*,3*R*,4*S*,5*S*)‐4‐[(*tert*‐butyldimethylsilyl)oxy]‐5‐[(1*R*)‐ and (1*S*)‐1‐[(*tert*‐butyldimethylsilyl)oxy]pent‐4‐en‐1‐yl]‐2‐(prop‐2‐en‐1‐yl)oxolan‐3‐yl]oxy})dimethylsilane (**1e**) (0.50 g, 0.88 mmol) in toluene (290 mL) under a N_2_ atmosphere and heated at 110 °C for 48 h. The solution was cooled and filtered through an Isolute NH_2_ basic ion exchange cartridge (20 g), eluting with toluene (200 mL). The filtrate was evaporated under vacuum then part (0.38 g) of the residue (0.48 g) was re‐dissolved in THF (13 mL). HCl (6 n, 0.7 mL) was added dropwise to the stirred solution at 0 °C under a N_2_ atmosphere. The solution was warmed to room temp. and stirred for 120 h then evaporated under vacuum. The residue was purified by column chromatography (10 % MeOH in CH_2_Cl_2_) to afford **2** as white crystals (0.052 g, 38 %), m.p. 175.4–183.1 °C. [*α*]_D_^23^ = +11.4 (*c* = 1.0, CH_2_Cl_2_). TLC *R*_f_ = 0.28 (10 % MeOH in CH_2_Cl_2_). IR (thin film): ν̃ = 3333, 3005, 2915, 1036 cm^–1^. ^1^H NMR (500 MHz, CD_3_OD): *δ* = 5.80–5.73 (m, 1 H, 5‐H), 5.71–5.65 (m, 1 H, 6‐H), 4.11 (dd, *J* = 7.5, 4.5 Hz, 1 H, 9‐H), 4.01–3.97 (m, 2 H, 8‐H and 10‐H), 3.66 (d, *J* = 9.5 Hz, 1 H, 1‐H), 3.25–3.19 (m, 1 H, 2‐H), 2.97–2.88 (m, 1 H, 4‐H), 2.63–2.56 (m, 1 H, 7‐H), 2.18 (ddd, *J* = 15.0, 8.5, 3.0 Hz, 1 H, 7‐H), 1.97–1.91 (m, 1 H, 4‐H), 1.84–1.72 (m, 2 H, 3‐H) ppm. ^13^C NMR (126 MHz, CD_3_OD): *δ* = 135.4, 126.3, 90.2, 83.5, 76.1, 73.5, 70.3, 35.4, 29.1, 26.5 ppm. LC‐MS (ESI^+^): *m/z* 201 [M + H^+^], 183.10 [M – H_2_O + H]^+^. HRMS (ESI^+^): [M + H^+^] calcd. for C_10_H_17_O_4_ 201.1121, found 201.1124.

**(1*S*,2*S*,5*Z*,8*R*,9*R*,13*S*)‐11,11‐Dimethyl‐10,12,14‐trioxatricyclo[6.5.1.0^9,13^]tetradec‐5‐en‐2‐ol (11):** 2,2‐Dimethoxypropane (0.10 mL, 0.82 mmol) and *p*‐toluenesulfonic acid (0.11 g, 0.58 mmol) were added to a stirred solution of (1*S*,2*S*,5*Z*,8*R*,9*S*,10*R*)‐11‐oxabicyclo[6.2.1]undec‐5‐ene‐2,9,10‐triol (**2**) (0.17 g, 0.82 mmol) in acetone (13.7 mL) and DMF (6.9 mL) under a N_2_ atmosphere. The solution was stirred at room temp. for 18 h then NaHCO_3_ solution (satd. aq., 45 mL) and water (45 mL) were added. The mixture was extracted with EtOAc (3 × 45 mL) and the combined organic layers were dried (Na_2_SO_4_), filtered and the solvents evaporated under vacuum. The residue was purified by column chromatography (30 % EtOAc in hexane) to afford **11** as white crystals (0.17 g, 86 %), m.p. 107.5–110.0 °C. [*α*]_D_^23^ = +68.4 (*c* = 1.0, CH_2_Cl_2_). TLC *R*_f_ = 0.45 (5 % MeOH in CH_2_Cl_2_). IR (thin film): ν̃ = 3238, 2985, 2919, 2855, 1070, 1041 cm^–1^. ^1^H NMR (500 MHz, CDCl_3_): *δ* = 5.76 (tdd, *J* = 11.0, 6.0, 1.5 Hz, 1 H, 5‐H), 5.66–5.59 (m, 1 H, 6‐H), 4.59 (dd, *J* = 5.5, 1.5 Hz, 1 H, 13‐H), 4.53 (dd, *J* = 5.5, 3.0 Hz, 1 H, 9‐H), 4.30–4.26 (m, 1 H, 8‐H), 4.00–3.95 (m, 1 H, 1‐H), 3.47–3.40 (m, 1 H, 2‐H), 2.82–2.72 (m, 1 H, 4‐H), 2.57–2.49 (m, 1 H, 7‐H), 2.12 (ddd, *J* = 14.5, 9.0, 4.5 Hz, 1 H, 7‐H), 2.02–1.86 (m, 2 H, 3‐H and 4‐H), 1.80–1.72 (m, 1 H, H3), 1.60–1.54 (m, 1 H, OH), 1.52 (s, 3 H, CH_3_), 1.33 (s, 3 H, CH_3_) ppm. ^13^C NMR (126 MHz, CDCl_3_): *δ* = 135.0, 124.4, 112.9, 88.1, 85.9, 85.2, 83.0, 71.8, 33.5, 30.6, 27.6, 25.7, 24.5 ppm. LC‐MS (ESI^+^): *m/z* 241 [M + H^+^], 223.13 [M – H_2_O + H]^+^. HRMS (ESI^+^): [M + H^+^] calcd. for C_13_H_21_O_4_ 241.1434, found 241.1437.

**(1*S*,2*S*,8*R*,9*S*,10*R*)‐11‐Oxabicyclo[6.2.1]undecane‐2,9,10‐triol (12):** (1*S*,2*S*,5*Z*,8*R*,9*R*,13*S*)‐11,11‐Dimethyl‐10,12,14‐trioxatricyclo[6.5.1.0^9,13^]tetradec‐5‐en‐2‐ol (**11**) was added to a stirred suspension of Pd on carbon (10 %, 0.010 g) in EtOAc (10 mL) under a N_2_ atmosphere. The mixture was bubbled with H_2_ for 5 min then stirred under H_2_ (1 atm) for 24 h at room temp. The mixture was filtered through Celite, washing with EtOAc and the filtrate was evaporated under vacuum. The residue was purified by column chromatography (33 % EtOAc in hexane) to afford (1*S*,2*S*,8*R*,9*R*,13*S*)‐11,11‐dimethyl‐10,12,14‐trioxatricyclo[6.5.1.0^9,13^]tetradecan‐2‐ol as white crystals (0.073 g, 72 %), m.p. 107.6–112.3 °C. [*α*]_D_^22^ = –7.1 (*c* = 1.0, CH_2_Cl_2_). TLC *R*_f_ = 0.37 (33 % EtOAc in hexane). IR (thin film): ν̃ = 3449, 2921, 2849, 1045, 865 cm^–1^. ^1^H NMR (500 MHz, CDCl_3_): *δ* = 4.82 (d, *J* = 6.0 Hz, 1 H, 13‐H), 4.51 (d, *J* = 6.0 Hz, 1 H, 9‐H), 4.21 (dd, *J* = 12.5, 4.0 Hz, 1 H, 8‐H), 3.84 (d, *J* = 10.0 Hz, 1 H, 1‐H), 3.57 (tdd, *J* = 9.5, 7.0, 3.0 Hz, 1 H, 2‐H), 2.06–1.88 (m, 2 H, 4‐H and 5‐H), 1.83–1.59 (m, 4 H, 3‐H, 6‐H and 7‐H), 1.59–1.50 (m, 4 H, 6‐H and CH_3_), 1.50–1.38 (m, 2.3 H, 4‐H, 5‐H and OH), 1.35 (d, *J* = 0.7 Hz, 3 H, CH_3_), 1.34–1.22 (m, 1 H, 7‐H) ppm. ^13^C NMR (126 MHz, CDCl_3_): *δ* = 112.2, 90.2, 86.1, 85.8, 85.0, 73.6, 38.5, 32.6, 27.6, 27.2, 26.6, 25.1, 24.9 ppm. LC‐MS (ESI^+^): *m/z* 225 [M – H_2_O + H]^+^. HRMS (ESI^+^): [M – H_2_O + H^+^] calcd. for C_13_H_21_O_3_ 225.1485, found 225.1486. HCl (1 m, 0.5 mL) was added to a stirred solution of (1*S*,2*S*,8*R*,9*R*,13*S*)‐11,11‐dimethyl‐10,12,14‐trioxatricyclo[6.5.1.0^9,13^]tetradecan‐2‐ol in MeCN (2.0 mL) under a N_2_ atmosphere. After 48 h the mixture was evaporated under vacuum and the residue was purified by column chromatography (10 % MeOH in CH_2_Cl_2_) to afford **12** as white crystals (0.018 g, 56 %), m.p. 147.6–151.9 °C. [*α*]_D_^24^ = +1.1 (*c* = 1.0, MeOH). TLC *R*_f_ = 0.36 (10 % MeOH in CH_2_Cl_2_). IR (thin film): ν̃ = 3271, 2912, 1078, 1037, 997 cm^–1^. ^1^H NMR (500 MHz, MeOD): *δ* = 4.15 (dd, *J* = 4.0, 2.5 Hz, 1 H, 10‐H), 4.08–4.02 (m, 2 H, 8‐H and 9‐H), 3.76 (dd, *J* = 7.5, 2.5 Hz, 1 H, 1‐H), 3.58 (ddd, *J* = 9.1, 7.7, 1.7 Hz, 1 H, 2‐H), 2.07–1.98 (m, 1 H, 4‐H), 1.92–1.82 (m, 1 H, 3‐H), 1.82–1.74 (m, 1 H, 7‐H), 1.72–1.52 (m, 5 H, 3‐H, 5‐H, 6‐H and 7‐H), 1.51–1.37 (m, 2 H, 4‐H and 6‐H) ppm. ^13^C NMR (126 MHz, MeOD): *δ* = 90.1, 84.9, 75.7, 75.1, 73.5, 37.2, 32.4, 30.6, 27.2, 25.0 ppm. LC‐MS (ESI^+^): *m/z* 185 [M – H_2_O + H^+^]. HRMS (ESI^+^): [M + Na^+^] calcd. for C_10_H_18_O_4_Na 225.1097, found 225.1104.

**(1*R*,2*R*,5*Z*,8*R*,9*S*,10*R*)‐11‐Oxabicyclo[6.2.1]undec‐5‐ene‐2,9,10‐triol (13):** Dess–Martin periodinane (0.064 g, 0.15 mmol) was added to a stirred solution of (1*S*,2*S*,5*Z*,8*R*,9*R*,13*S*)‐11,11‐dimethyl‐10,12,14‐trioxatricyclo[6.5.1.0^9,13^]tetradec‐5‐en‐2‐ol (**11**) in CH_2_Cl_2_ under a N_2_ atmosphere. After 3 h Na_2_SO_3_ solution (satd. aq., 5 mL) was added and the mixture was stirred for 5 min. NaHCO_3_ solution (satd. aq., 5 mL) and water (15 mL) were added and the mixture was extracted with CH_2_Cl_2_ (3 × 15 mL). The combined organic layers were dried (Na_2_SO_2_), filtered and the solvents evaporated under vacuum. The residue was purified by column chromatography (15 % EtOAc in hexane) to afford (1*R*,5*Z*,8*R*,9*R*,13*R*)‐11,11‐dimethyl‐10,12,14‐trioxatricyclo[6.5.1.0^9,13^]tetradec‐5‐en‐2‐one as white crystals (0.027 g, 94 %), m.p. 80.6–83.3 °C. [*α*]_D_^22^ = –31.6 (*c* = 1.0, CH_2_Cl_2_). TLC *R*_f_ = 0.38 (15 % EtOAc in hexane). IR (thin film): ν̃ = 3015, 2927, 1702, 1056 cm^–1^. ^1^H NMR (500 MHz,CDCl_3_): *δ* = 5.87 (td, *J* = 10.0, 6.5 Hz, 1 H, 5‐H), 5.65–5.57 (m, 1 H, 6‐H), 5.18 (d, *J* = 6.0 Hz, 1 H, 13‐H), 4.48 (d, *J* = 6.0 Hz, 1 H, 9‐H), 4.41 (dd, *J* = 8.5, 3.0 Hz, 1 H, 8‐H), 4.30 (d, *J* = 1.0 Hz, 1 H, 1‐H), 2.94 (ddd, *J* = 12.0, 7.0, 4.5 Hz, 1 H, 3‐H), 2.77 (s, 1 H, 4‐H), 2.40–2.24 (m, 2 H, 3‐H and 7‐H), 2.19–2.11 (m, 1 H, 4‐H), 1.82 (dt, *J* = 14.0, 9.0 Hz, 1 H, 7‐H), 1.49 (s, 3 H, CH_3_), 1.34 (s, 3 H, CH_3_) ppm. ^13^C NMR (126 MHz, CDCl_3_): *δ* = 214.1, 133.3, 126.4, 112.6, 90.8, 86.4, 84.3, 82.5, 40.0, 30.9, 26.7, 25.3, 23.7 ppm. LC‐MS (ESI^+^): *m/z* 239 [M + H^+^]. HRMS (ESI^+^): [M + H^+^] calcd. for C_13_H_19_O_4_ 239.1278, found 239.1277. A solution of (1*R*,5*Z*,8*R*,9*R*,13*R*)‐11,11‐dimethyl‐10,12,14‐trioxatricyclo[6.5.1.0^9,13^]tetradec‐5‐en‐2‐one (0.018 g, 0.076 mmol) in MeOH (2.0 mL) under a N_2_ atmosphere was cooled to 0 °C for 10 min then NaBH_4_ (0.006 g, 0.15 mmol) was added and the mixture was stirred at 0 °C for 30 min. NH_4_Cl solution (satd. aq., 5 mL) was added and the mixture was poured into water (10 mL). The mixture was extracted with EtOAc (2 × 15 mL) and the combined organic layers were dried (Na_2_SO_4_), filtered and the solvents evaporated under vacuum. The residue was purified by column chromatography (33 % EtOAc in hexane) to afford a 7:1 (*R/S*) epimeric mixture of (2*S*)‐ and (2*R*)‐(1*S*,5*Z*,8*R*,9*R*,13*S*)‐11,11‐dimethyl‐10,12,14‐trioxatricyclo[6.5.1.0^9,13^]tetradec‐5‐en‐2‐ol as white crystals (0.013 g, 71 %). TLC *R*_f_ = 0.25 (33 % EtOAc in hexane). IR (thin film): ν̃ = 3214, 3008, 2920, 2896, 1215, 1070 cm^–1^. ^1^H NMR (500 MHz, CDCl_3_): *δ* = (diastereomeric ratio 7:1) 5.75 (tdd, *J* = 11.5, 6.0, 2.0 Hz, 1 H, 5‐H), 5.66–5.59 (m, 0.13 H, 6‐H), 5.56–5.49 (m, 0.88 H, 6‐H), 4.72 (t, *J* = 5.5 Hz, 0.88 H, 13‐H), 4.60 (dd, *J* = 5.5, 1.5 Hz, 0.13 H, 13‐H), 4.54 (d, *J* = 5.8 Hz, 1 H, 9‐H), 4.47 (dd, *J* = 4.4, 2.4 Hz, 0.88 H, 8‐H), 4.30–4.27 (m, 0.13 H, 8‐H), 4.18 (app‐t, *J* = 4.5 Hz, 0.88 H, 1‐H), 4.12–4.06 (m, 0.88 H, 2‐H), 4.00–3.96 (m, 0.13 H, 1‐H), 3.47–3.40 (m, 0.13 H, 2‐H), 2.82–2.73 (m, 0.13 H, 4‐H), 2.64–2.44 (m, 1.88 H, 4‐H and 7‐H), 2.18–2.09 (m, 1.88 H, 3‐H and 7‐H), 2.02–1.87 (m, 1.13 H, 3‐H and 4‐H), 1.79–1.72 (m, 0.13 H, 3‐H), 1.56–1.48 (m, 3.88 H, 3‐H and CH_3_), 1.34–1.31 (m, 3 H, CH_3_) ppm. ^13^C NMR (126 MHz, CDCl_3_): *δ* = 136.7, 135.0, 124.4, 123.6, 112.9, 112.6, 89.0, 88.1, 85.9, 85.1, 84.0, 83.5, 83.0, 79.8, 71.8, 70.0, 33.5, 31.7, 30.6, 28.0, 27.6, 25.6, 24.5, 22.0 ppm. LC‐MS (ESI^+^): *m/z* 241 [M + H^+^]. HRMS (ESI^+^): [M + H^+^] calcd. for C_13_H_21_O_4_ 241.1434, found 241.1432. HCl (6 m, 0.1 mL) was added to a stirred solution of (2*S*)‐ and (2*R*)‐(1*S*,5*Z*,8*R*,9*R*,13*S*)‐11,11‐dimethyl‐10,12,14‐trioxatricyclo[6.5.1.0^9,13^]tetradec‐5‐en‐2‐ol (0.013 g, 0.054 mmol) in MeOH (1.9 mL) under a N_2_ atmosphere. After 24 h the mixture was evaporated under vacuum. The residue was purified by column chromatography (10 % MeOH in CH_2_Cl_2_) to afford **13** as a colourless gum (0.004 g, 37 %). [*α*]_D_^22^ = +13 (*c* = 1.0, MeOH). TLC *R*_f_ = 0.28 (10 % MeOH in CH_2_Cl_2_). IR (thin film): ν̃ = 3340, 3007, 2920, 1054 cm^–1^. ^1^H NMR (500 MHz, MeOD): *δ* = 5.73 (tdd, *J* = 11.5, 6.0, 2.0 Hz, 1 H, 5‐H), 5.56–5.45 (m, 1 H, 6‐H), 4.43 (dd, *J* = 8.0, 5.5 Hz, 1 H, 10‐H), 4.15 (t, *J* = 3.0 Hz, 1 H, 8‐H), 4.08–4.02 (m, 1 H, 2‐H), 3.98 (dd, *J* = 8.0, 3.5 Hz, 1 H, 1‐H), 3.93 (d, *J* = 5.5 Hz, 1 H, 9‐H), 2.62–2.54 (m, 1 H, 4‐H), 2.43–2.35 (m, 1 H, 7‐H), 2.14–2.04 (m, 2 H, 3‐H and 7‐H), 1.99–1.91 (m, 1 H, 4‐H), 1.60–1.50 (m, 1 H, 3‐H) ppm. ^13^C NMR (126 MHz, MeOD): *δ* = 137.7, 124.6, 86.7, 86.1, 75.9, 72.4, 71.5, 32.2, 31.4, 23.1 ppm. LC‐MS (ESI^+^): *m/z* 201 [M + H^+^]. HRMS (ESI^+^): [M + H^+^] calcd. for C_10_H_17_O_4_ 201.1121, found 201.1124.

## Supporting information

Supporting Information
