# Supplementary material for: Synthesis of a Ribose‐Incorporating Medium Ring Scaffold via a Challenging Ring‐Closing Metathesis Reaction
Source: European J Org Chem. 2016 Aug 15;2016(26):4496–507. doi: 10.1002/ejoc.201600756 (PMC5157775; doi:10.1002/ejoc.201600756)

Eur. J. Org. Chem. **2016** · ISSN 1099–0690

SUPPORTING INFORMATION

DOI: 10.1002/ejoc.201600756
Title: Synthesis of a Ribose-Incorporating Medium Ring Scaffold via a Challenging Ring-Closing Metathesis Reaction
Author(s): Stuart S. Rankin, John J. Caldwell, Nora B. Cronin, Rob L. M. van Montfort, Ian Collins*

Figure S1: ^{1}H NMR spectra for the original mixture of epimers of the bisalkene triol, above, and the deprotected, uncyclised bisalkene triol isolated after the RCM reaction, below. Signals for the major (a) and minor (b) diastereoisomers of the original mixture are annotated. Hydroxyl protons are not visible in the lower spectrum.

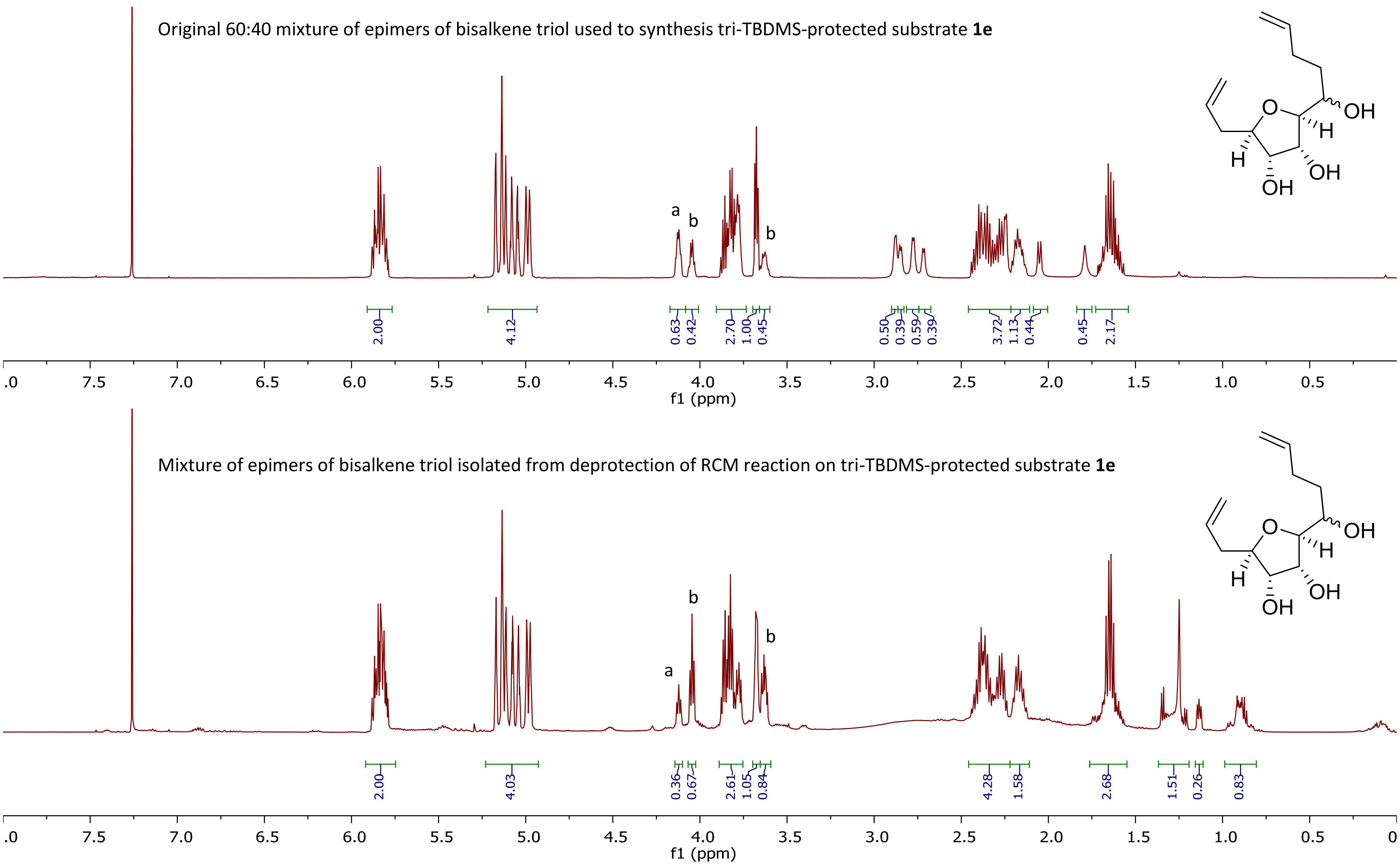

^{1}H and ^{13}C NMR spectra

[(3a*S*,4*S*,6*R*,6a*S*)-6-(acetyloxy)-2,2-dimethyl-tetrahydro-2H-furo[3,4-*d*][1,3]dioxol-4-yl]methyl acetate (6) ^{1}H NMR

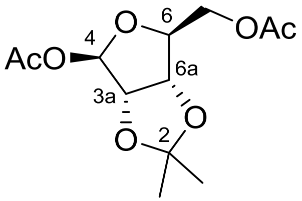

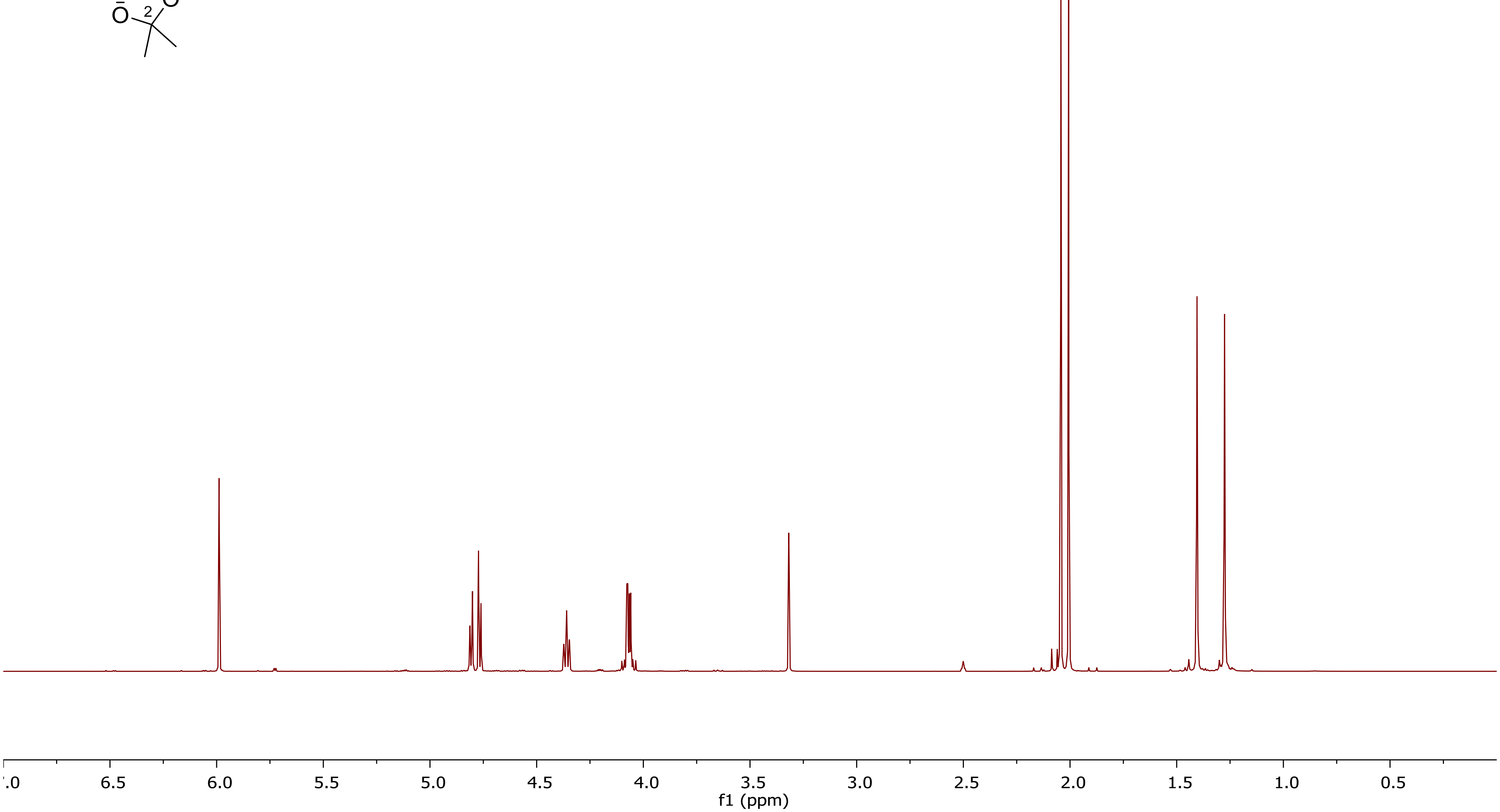

[(3a*S*,4*S*,6*R*,6a*S*)-6-(acetyloxy)-2,2-dimethyl-tetrahydro-2H-furo[3,4-*d*][1,3]dioxol-4-yl]methyl acetate (6) DEPTq ^{13}C NMR

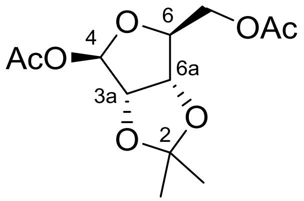

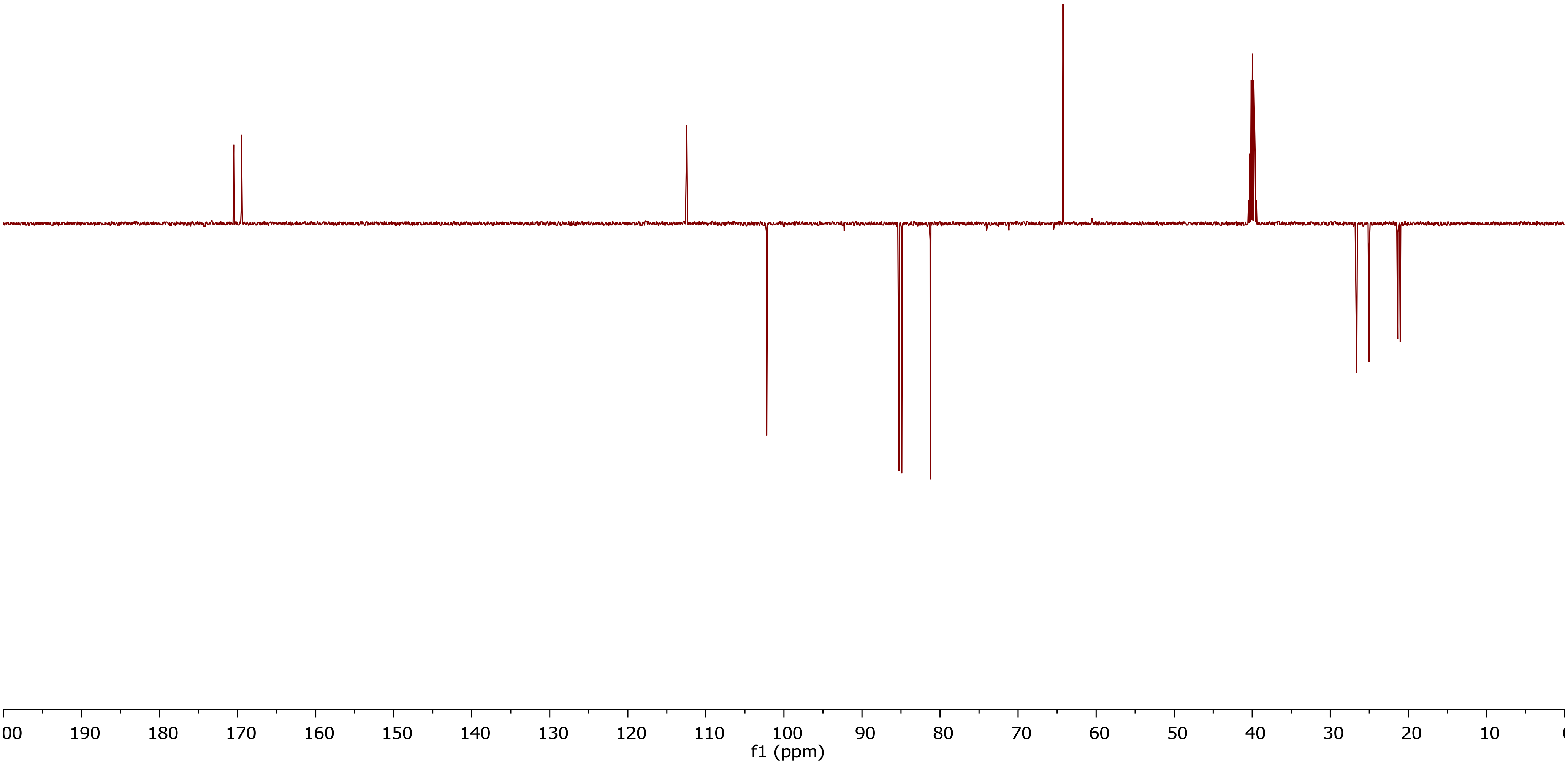

[(3a*S*,4*S*,6*R*,6a*R*)-2,2-dimethyl-6-(prop-2-en-1-yl)-tetrahydro-2H-furo[3,4-*d*][1,3]dioxol-4-yl]methanol (7) ^{1}H NMR

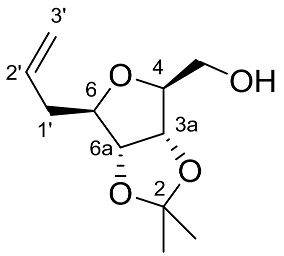

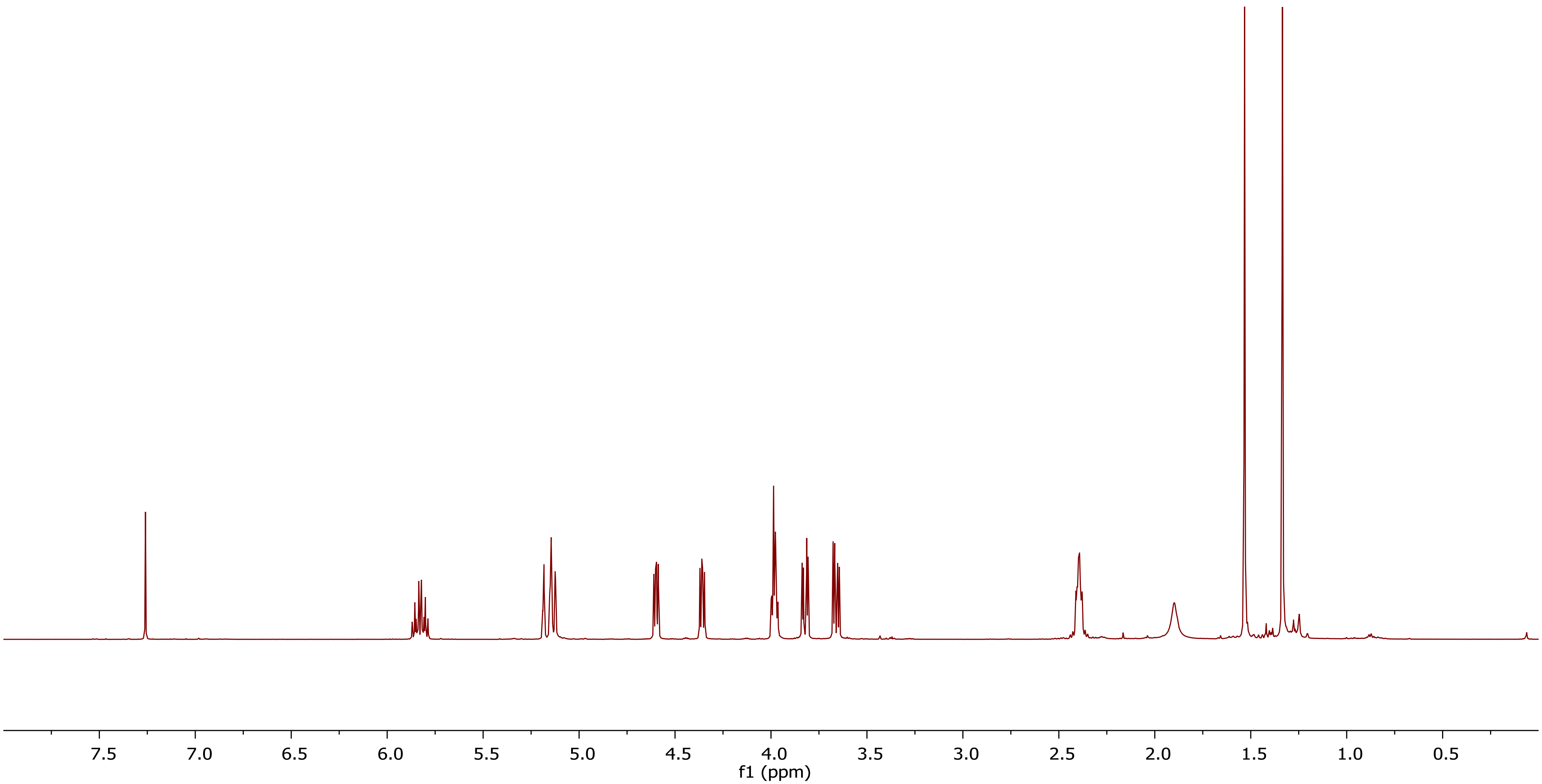

[(3a*S*,4*S*,6*R*,6a*R*)-2,2-dimethyl-6-(prop-2-en-1-yl)-tetrahydro-2H-furo[3,4-*d*][1,3]dioxol-4-yl]methanol (7) DEPTq ^{13}C NMR

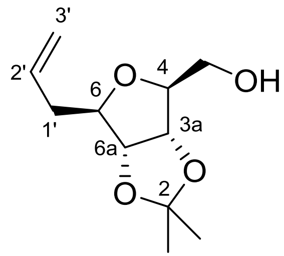

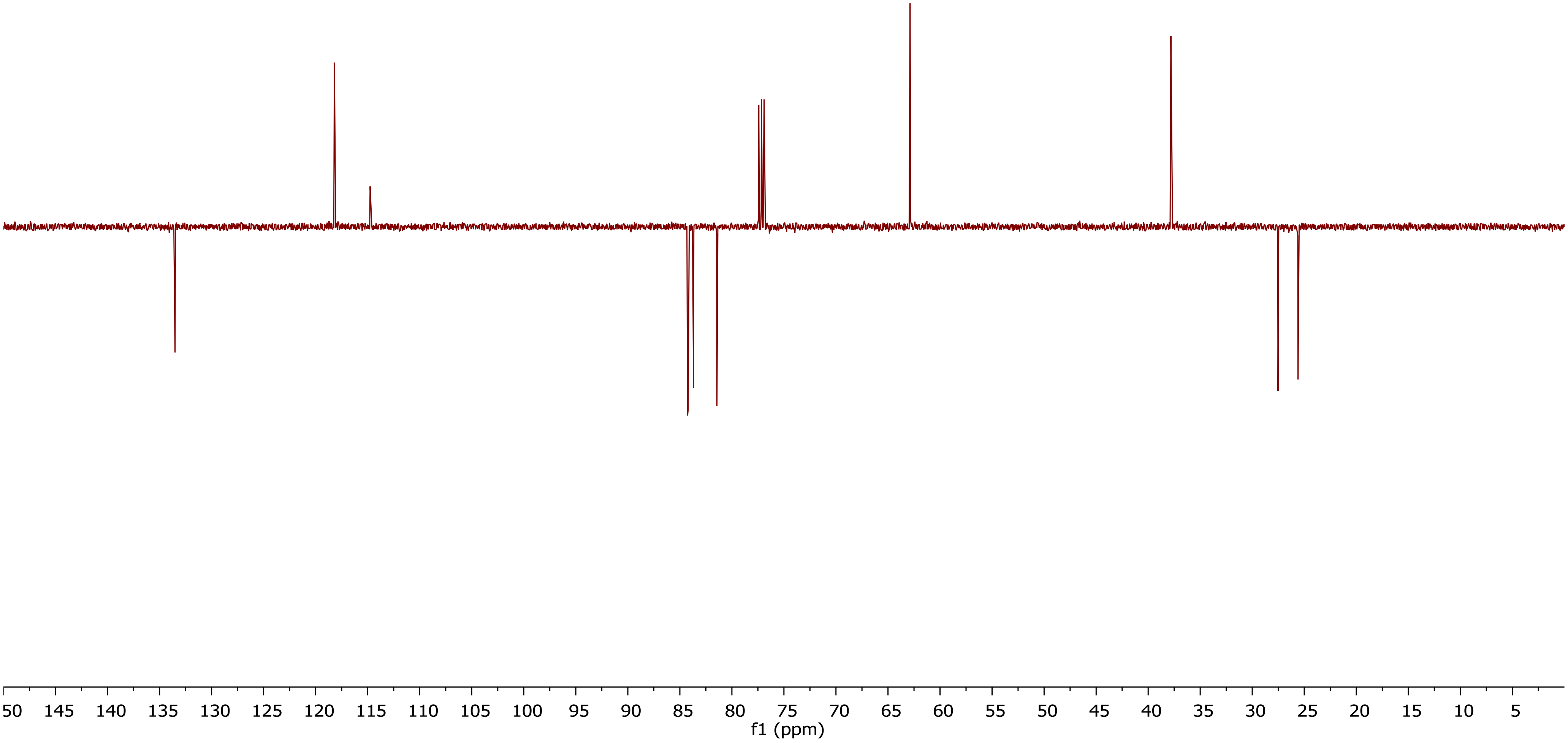

(1*S*)- and (1*R*)-1-[(3a*S*,4*S*,6*R*,6a*R*)-2,2-dimethyl-6-(prop-2-en-1-yl)-tetrahydro-2H-furo[3,4-*d*][1,3]dioxol-4-yl]pent-4-en-1-ol (9) ^{1}H NMR

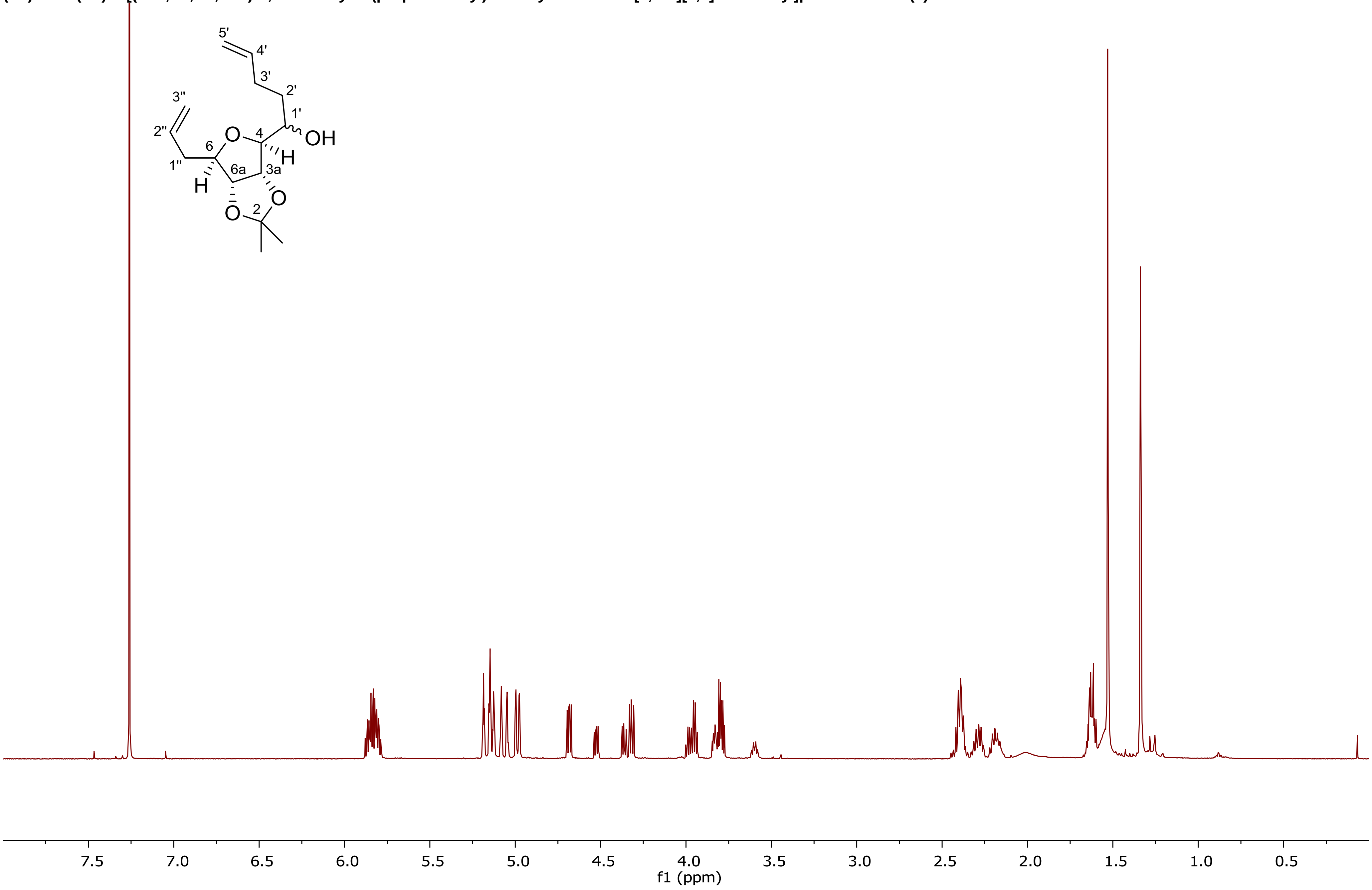

(1*S*)- and (1*R*)-1-[(3a*S*,4*S*,6*R*,6a*R*)-2,2-dimethyl-6-(prop-2-en-1-yl)-tetrahydro-2H-furo[3,4-*d*][1,3]dioxol-4-yl]pent-4-en-1-ol (9) DEPTq ^{13}C NMR

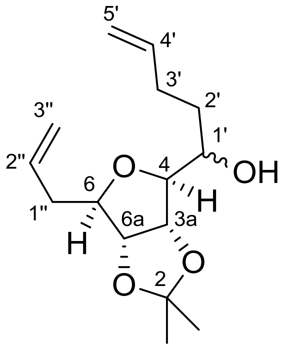

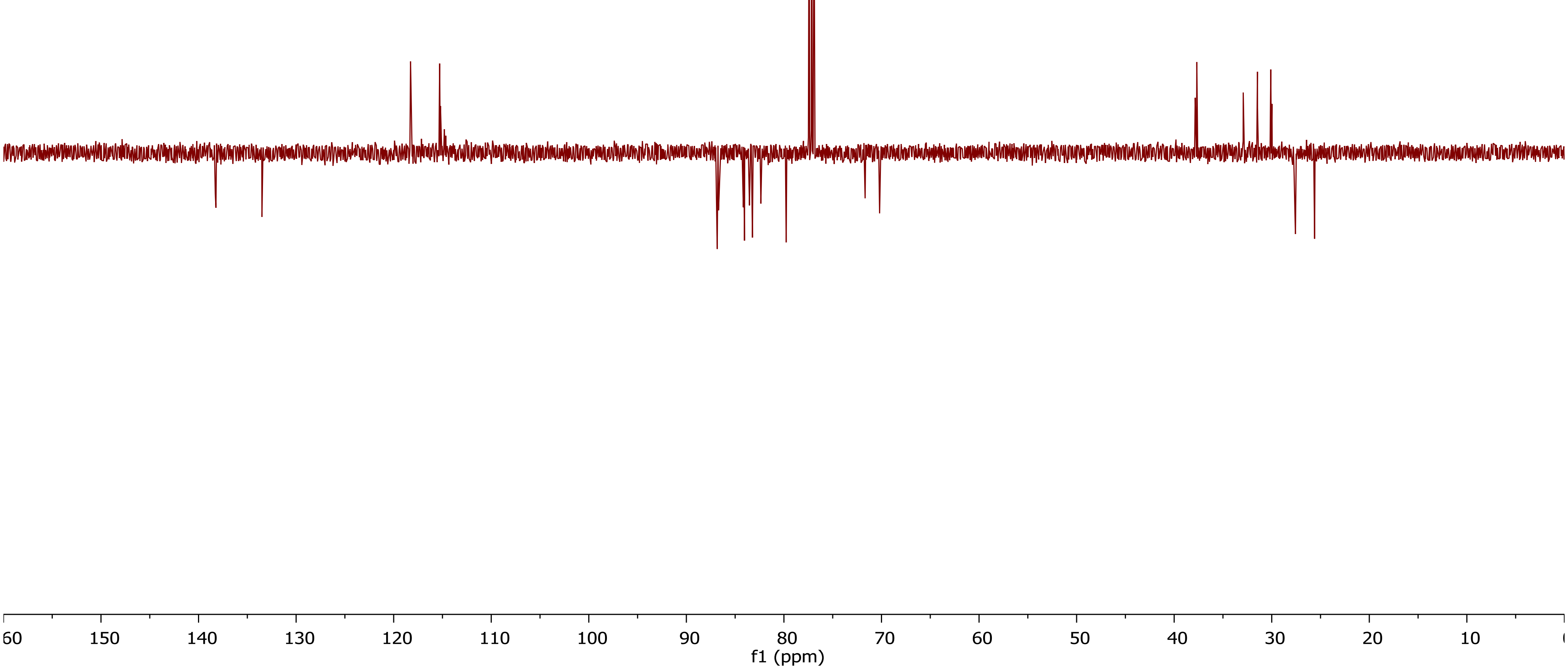

(1*S*)- and (1*R*)- (3a*S*,4*S*,6*R*,6a*R*)-2,2-dimethyl-4-[1-(naphthalen-2-ylmethoxy)pent-4-en-1-yl]-6-(prop-2-en-1-yl)-tetrahydro-2H-furo[3,4-*d*][1,3]dioxole (1a) ^{1}H NMR

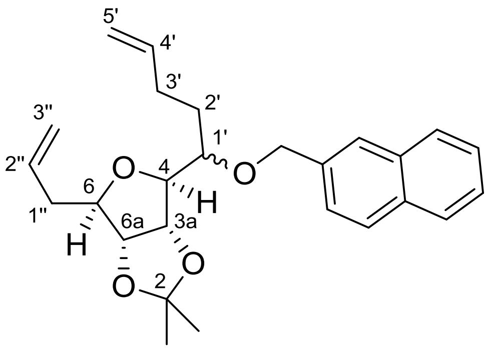

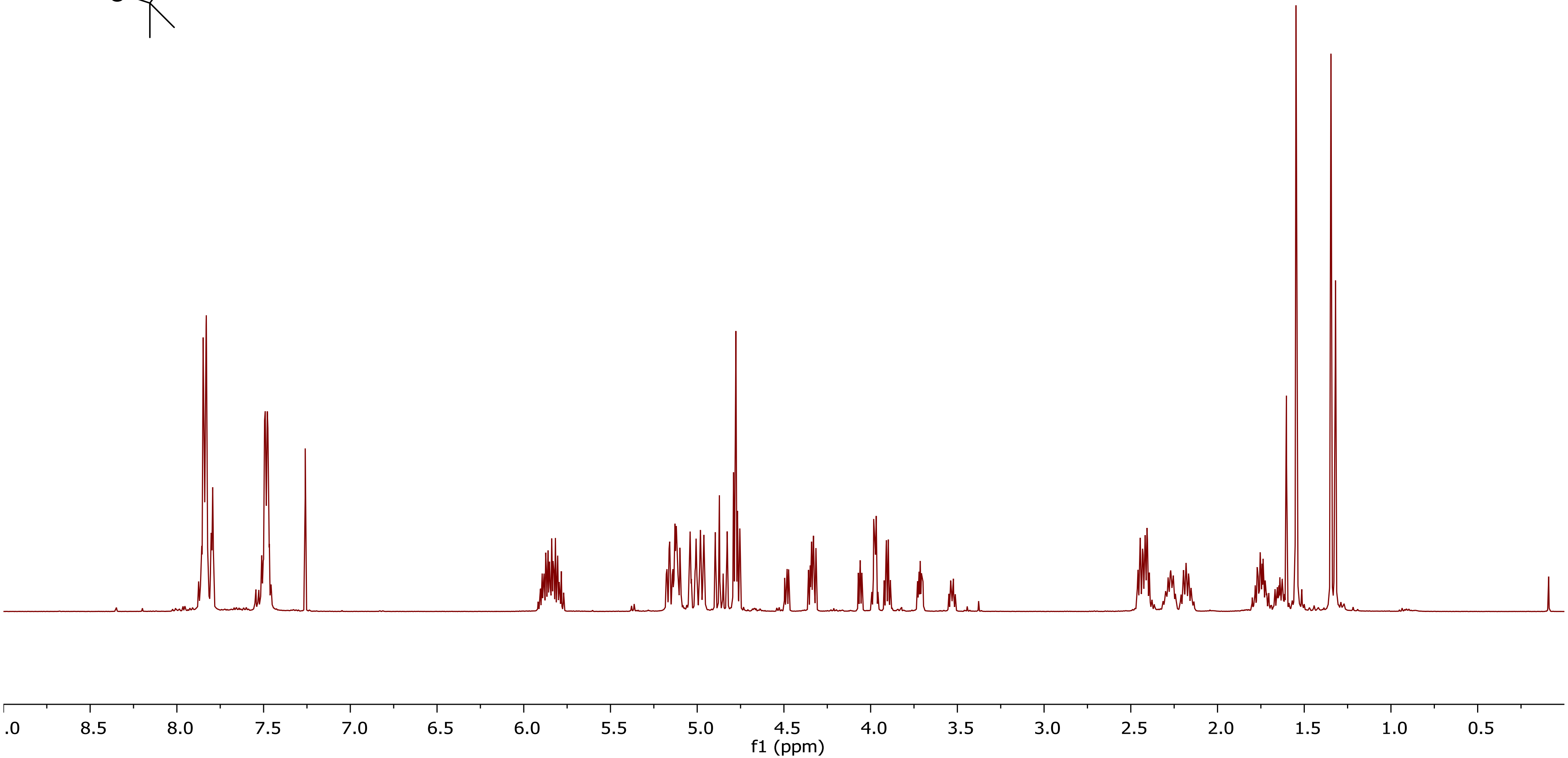

(1*S*)- and (1*R*)- (3a*S*,4*S*,6*R*,6a*R*)-2,2-dimethyl-4-[1-(naphthalen-2-ylmethoxy)pent-4-en-1-yl]-6-(prop-2-en-1-yl)-tetrahydro-2H-furo[3,4-*d*][1,3]dioxole (1a) DEPTq ^{13}C NMR

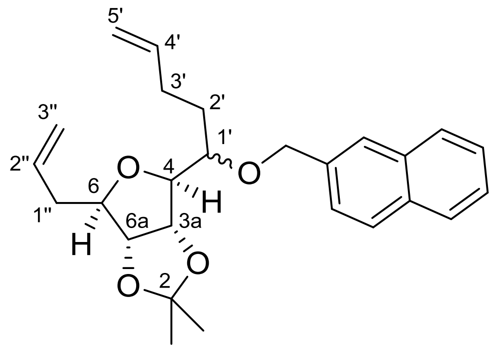

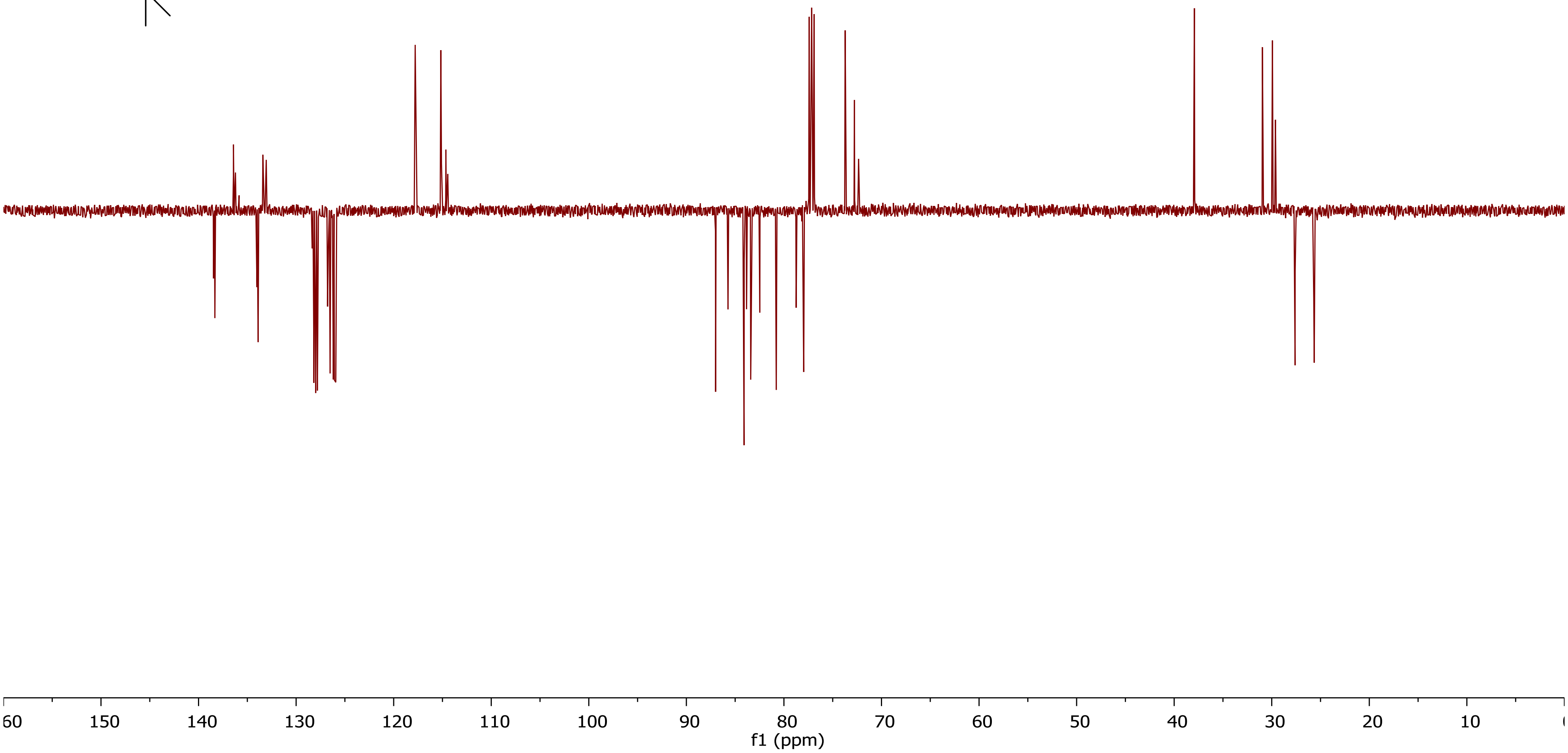

(1*S*)- and (1*R*)-(2*R*,3*R*,4*S*,5*R*)-2-[1-(naphthalen-2-ylmethoxy)pent-4-en-1-yl]-5-(prop-2-en-1-yl)oxolane-3,4-diol ^{1}H NMR

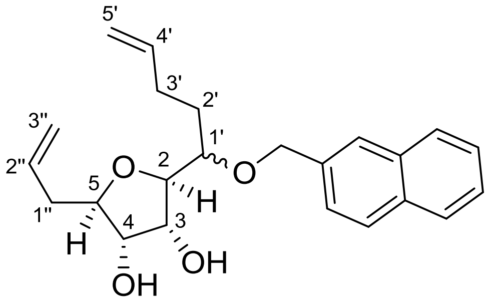

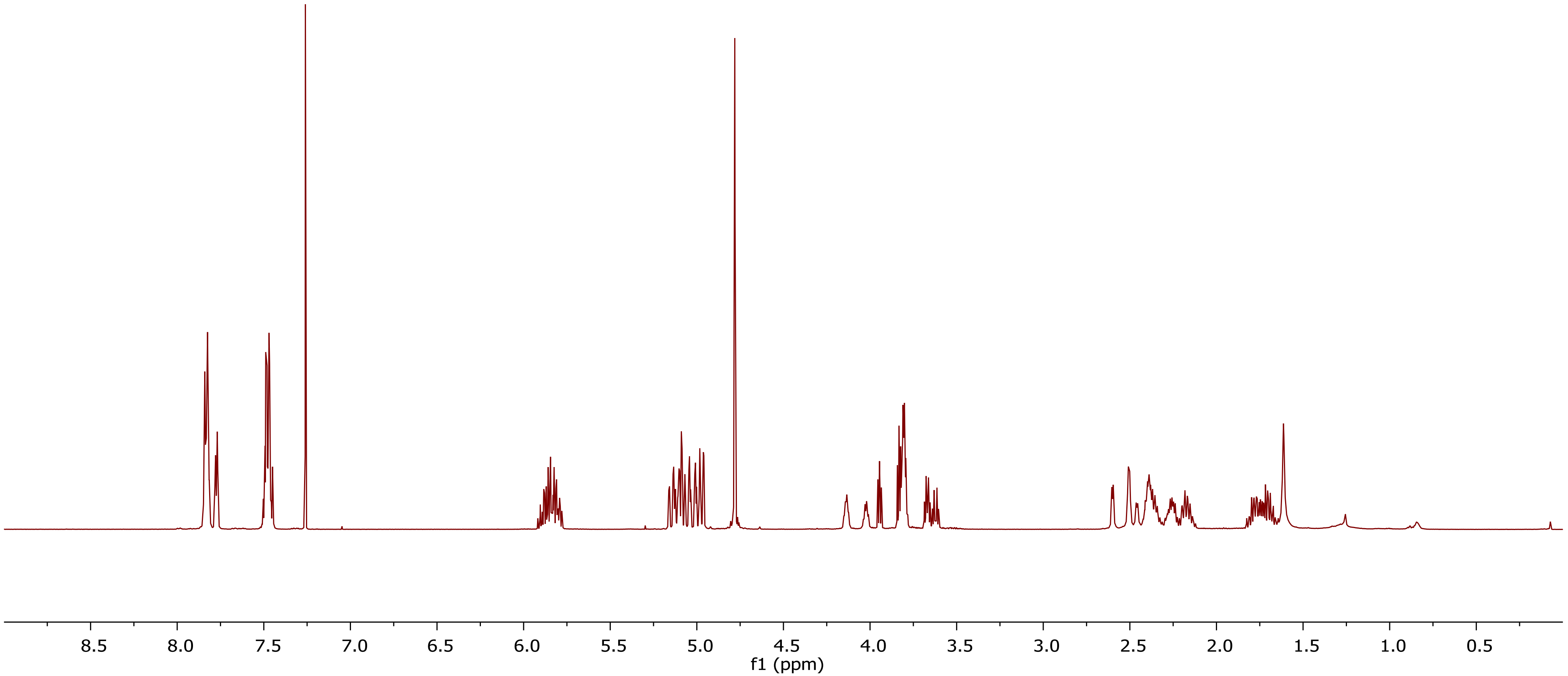

(1*S*)- and (1*R*)-(2*R*,3*R*,4*S*,5*R*)-2-[1-(naphthalen-2-ylmethoxy)pent-4-en-1-yl]-5-(prop-2-en-1-yl)oxolane-3,4-diol DEPTq ^{13}C NMR

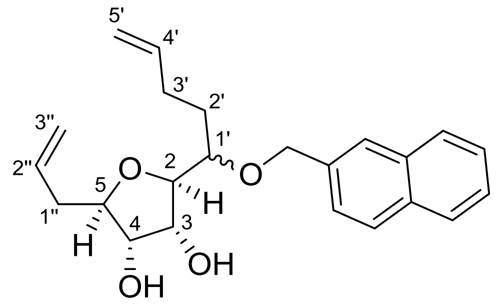

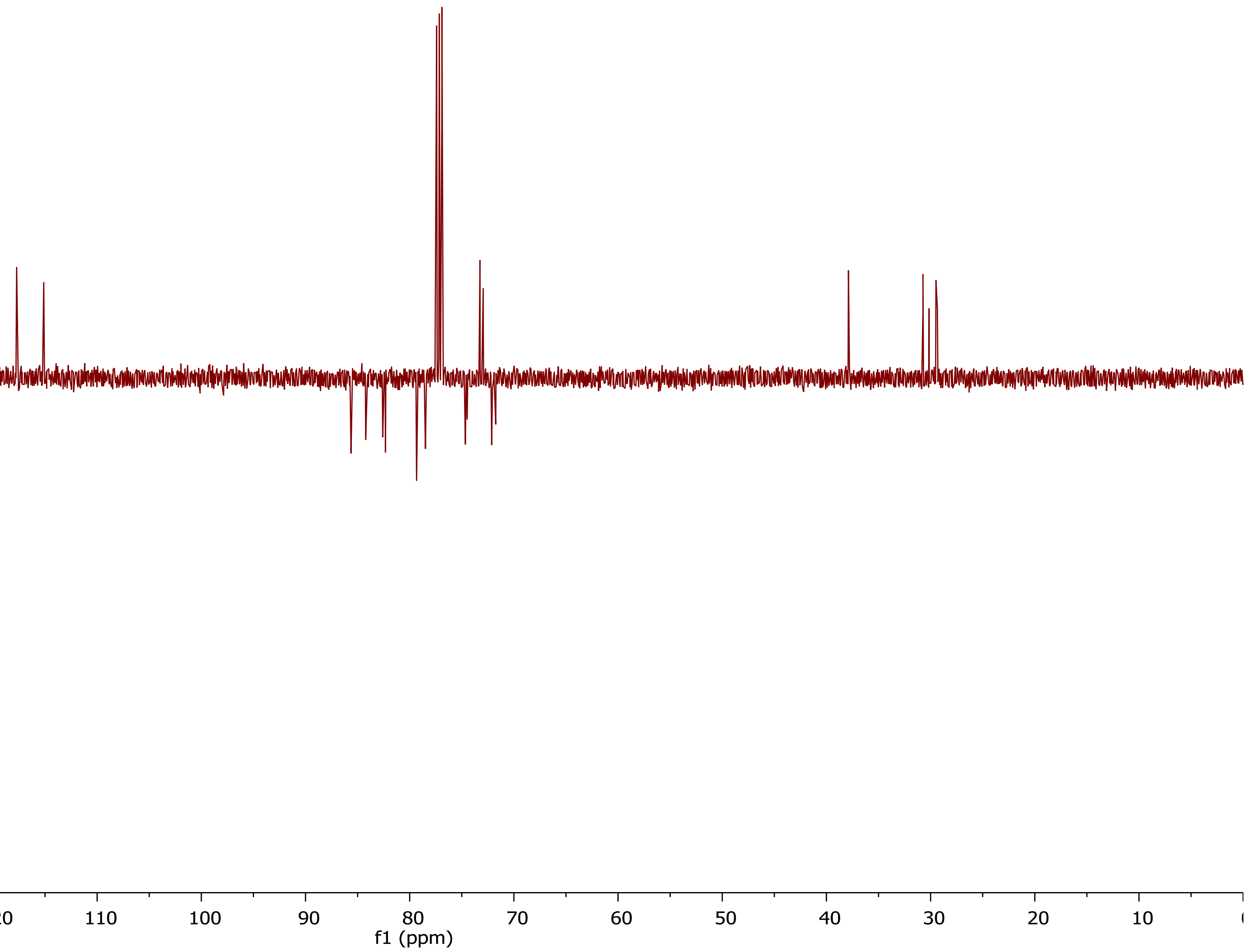

(1*S*)- and (1*R*)- (2S,3R,4R,5R)-3,4-bis(benzyloxy)-2-[1-(naphthalen-2-ylmethoxy)pent-4-en-1-yl]-5-(prop-2-en-1-yl)oxolane (1b) ^{1}H NMR

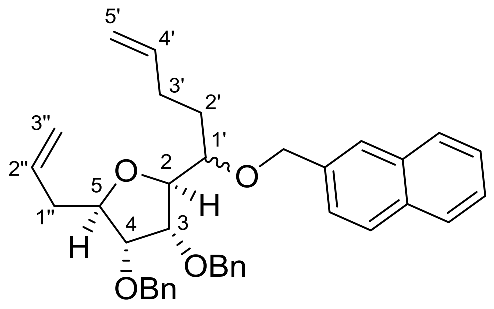

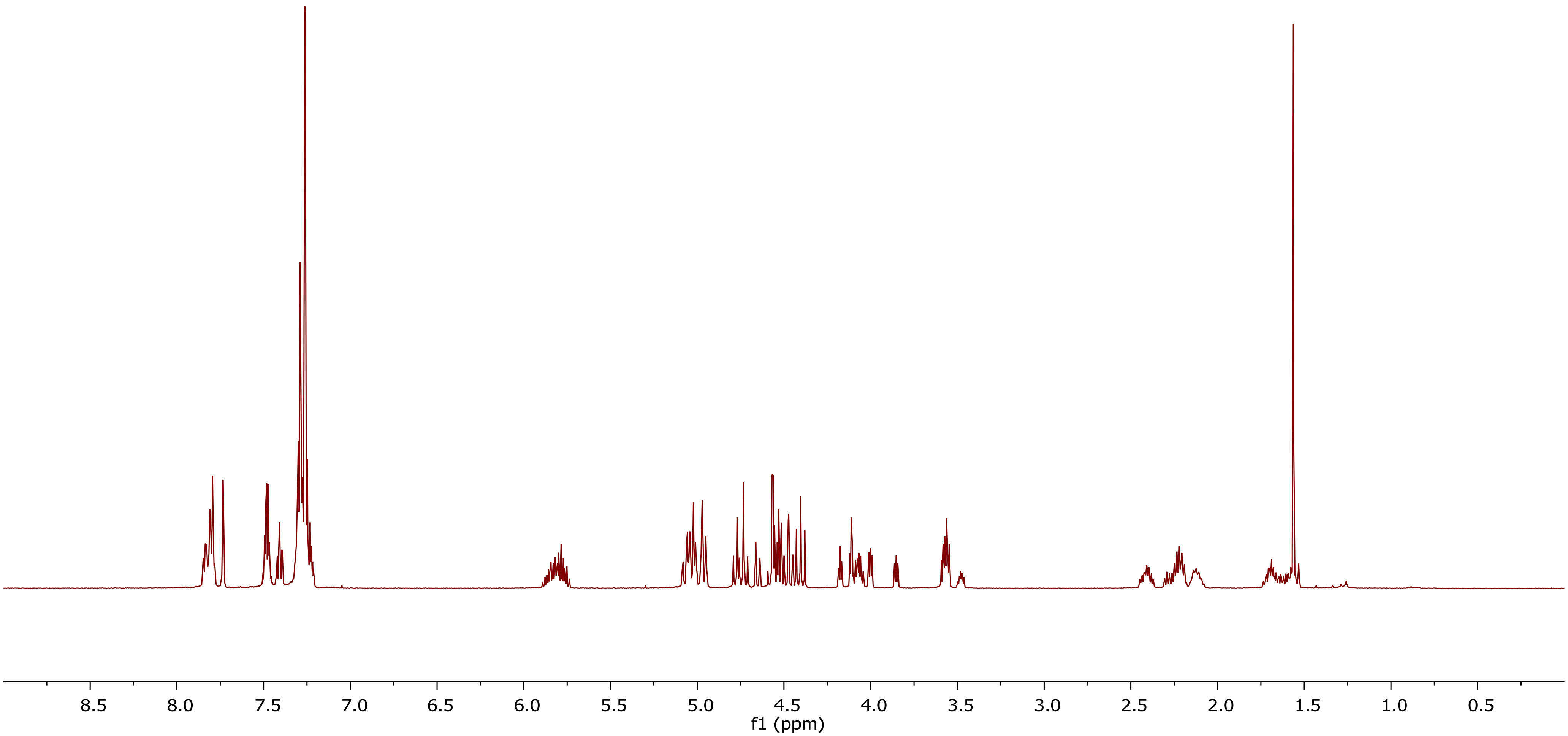

(1*S*)- and (1*R*)- (2S,3R,4R,5R)-3,4-bis(benzyloxy)-2-[1-(naphthalen-2-ylmethoxy)pent-4-en-1-yl]-5-(prop-2-en-1-yl)oxolane (1b) DEPTq ^{13}C NMR

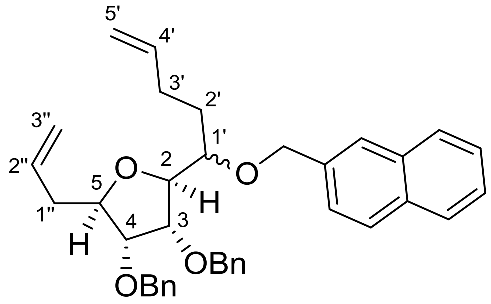

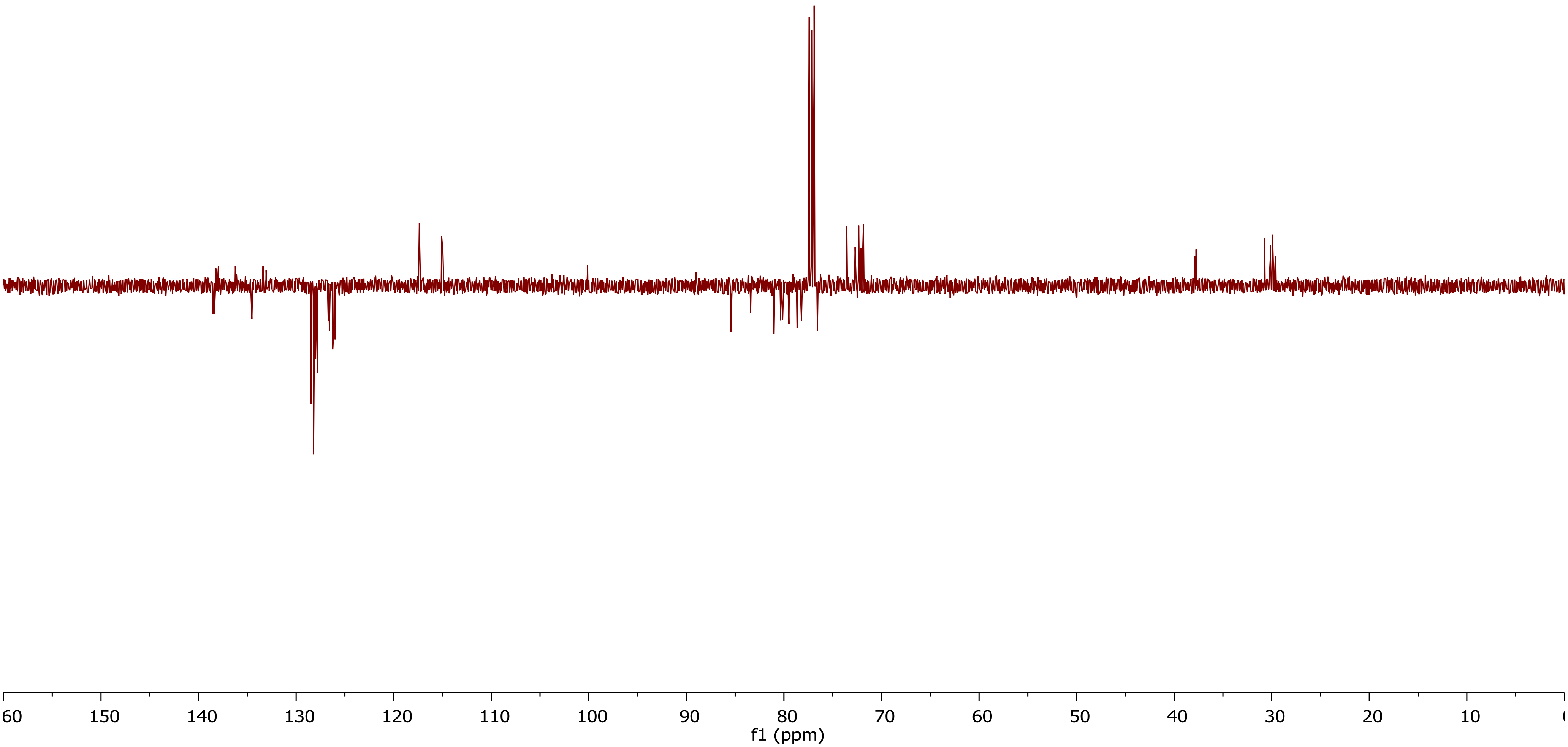

(1*R*)- and (1*S*)-*tert*-butyl({[(2*R*,3*R*,4*S*,5*S*)-4-[(*tert*-butyldimethylsilyl)oxy]-5-[1-(naphthalen-2-ylmethoxy)pent-4-en-1-yl]-2-(prop-2-en-1-yl)oxolan-3-yl]oxy})dimethylsilane (1c) ^{1}H NMR

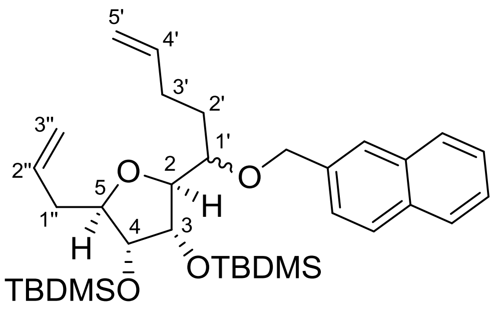

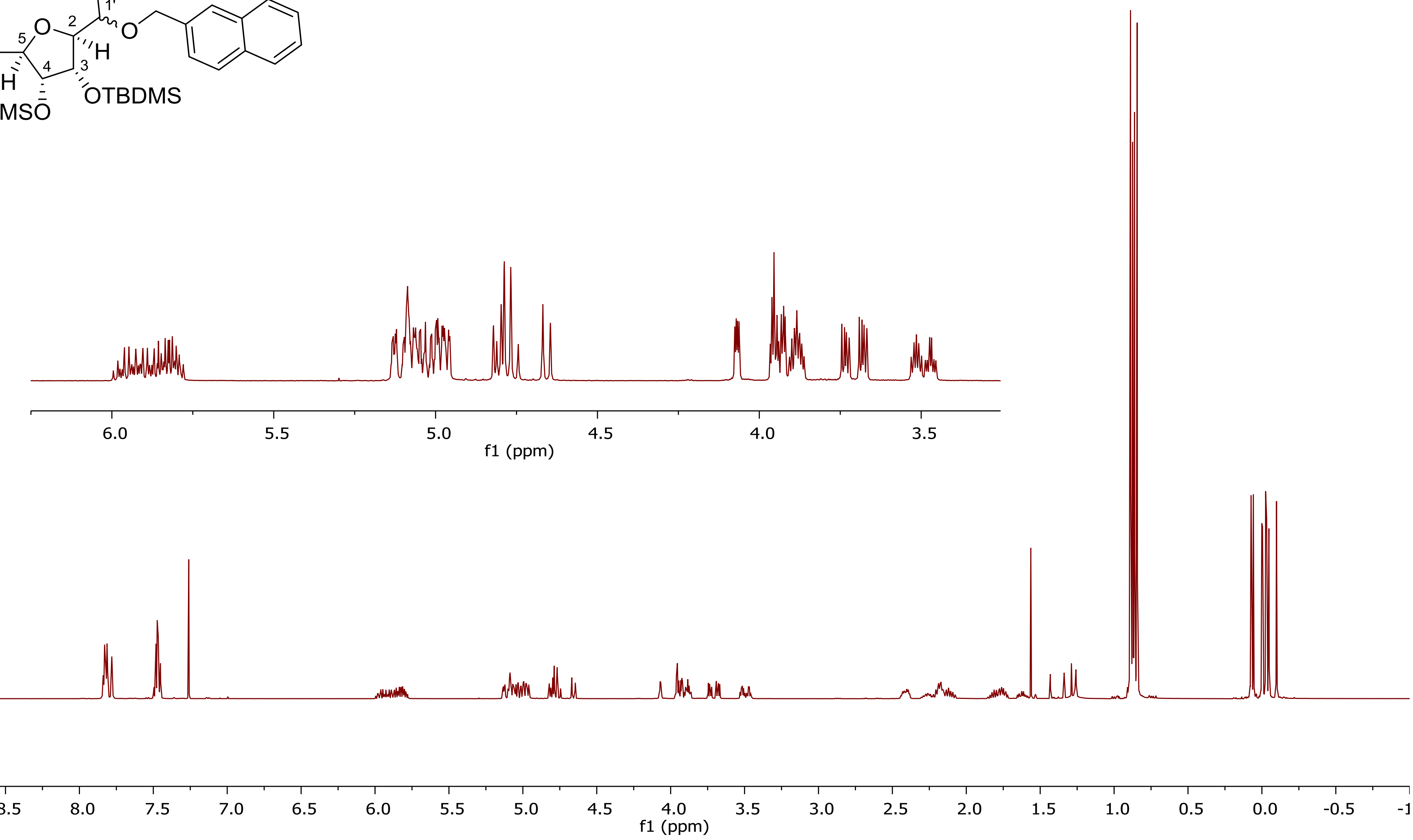

(1*R*)- and (1*S*)-*tert*-butyl({[(2*R*,3*R*,4*S*,5*S*)-4-[(*tert*-butyldimethylsilyl)oxy]-5-[1-(naphthalen-2-ylmethoxy)pent-4-en-1-yl]-2-(prop-2-en-1-yl)oxolan-3-yl]oxy})dimethylsilane (1c) DEPTq ^{13}C NMR

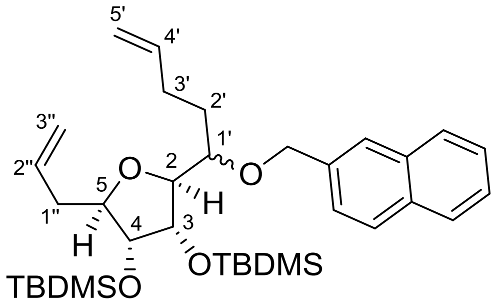

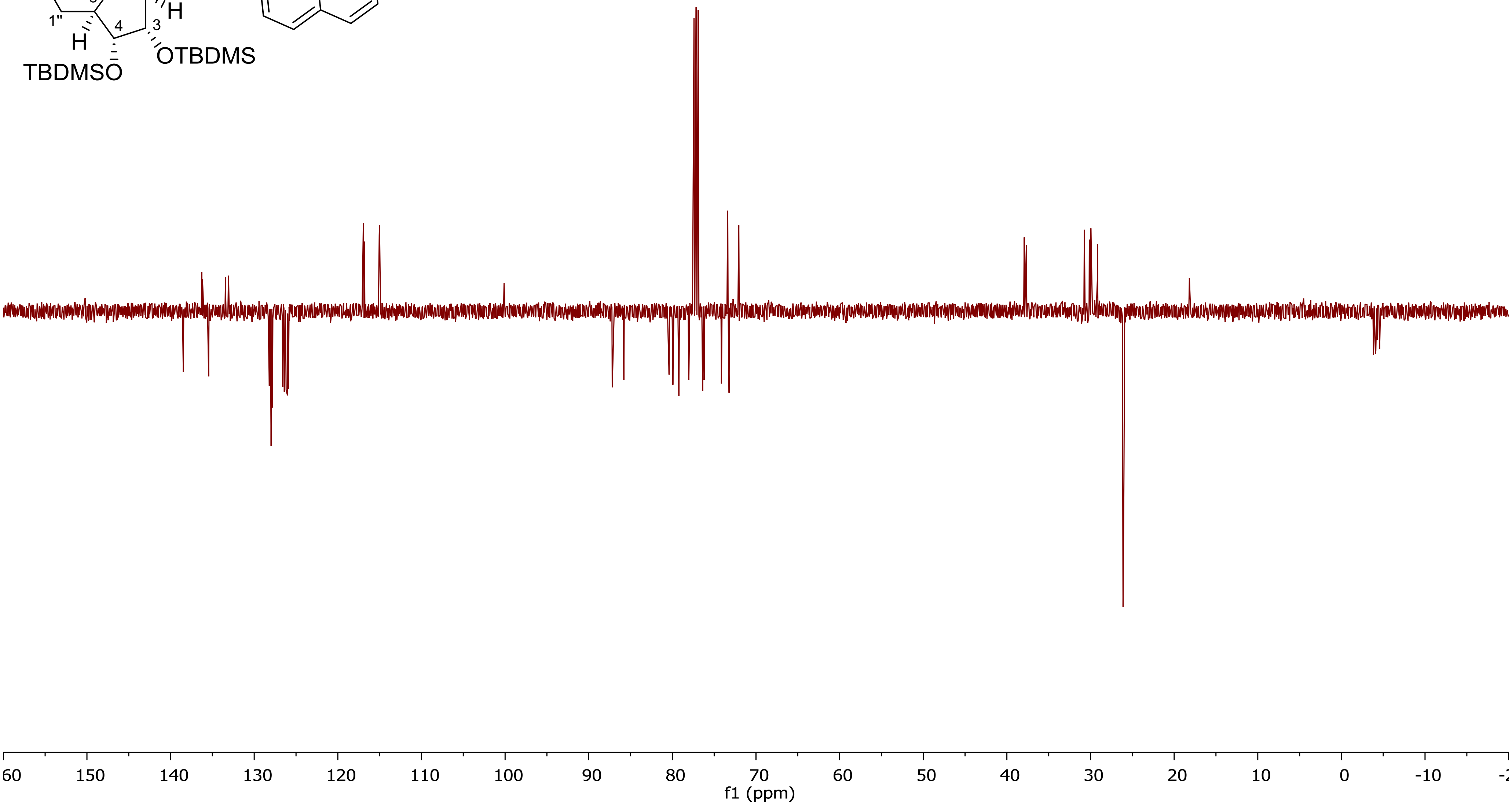

(1*S*)- and (1*R*)-(2*S*,3*R*,4*S*,5*R*)-2-[1-hydroxypent-4-en-1-yl]-5-(prop-2-en-1-yl)oxolane-3,4-diol ^{1}H NMR

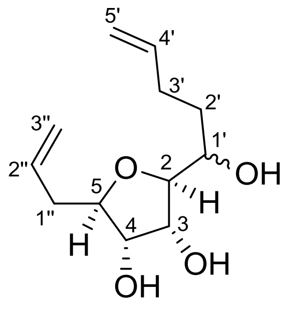

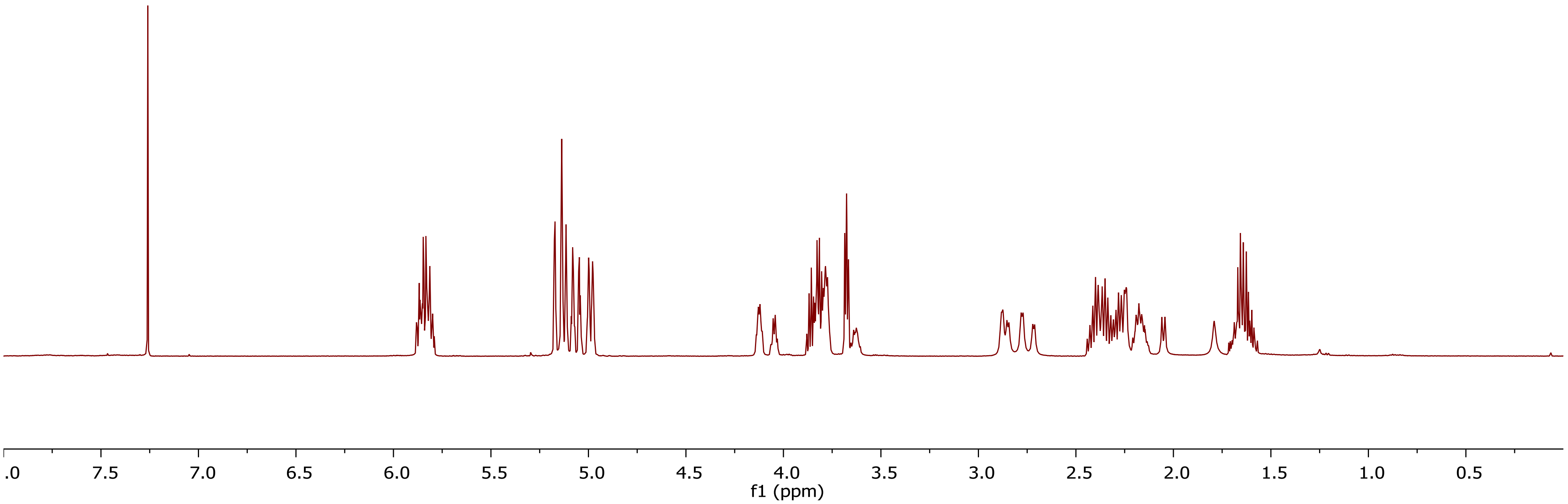

(1*S*)- and (1*R*)-(2*S*,3*R*,4*S*,5*R*)-2-[1-hydroxypent-4-en-1-yl]-5-(prop-2-en-1-yl)oxolane-3,4-diol UDEFT ^{13}C NMR

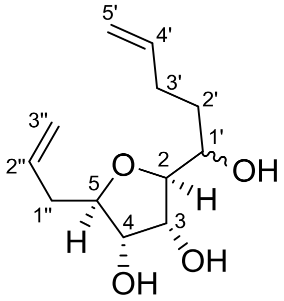

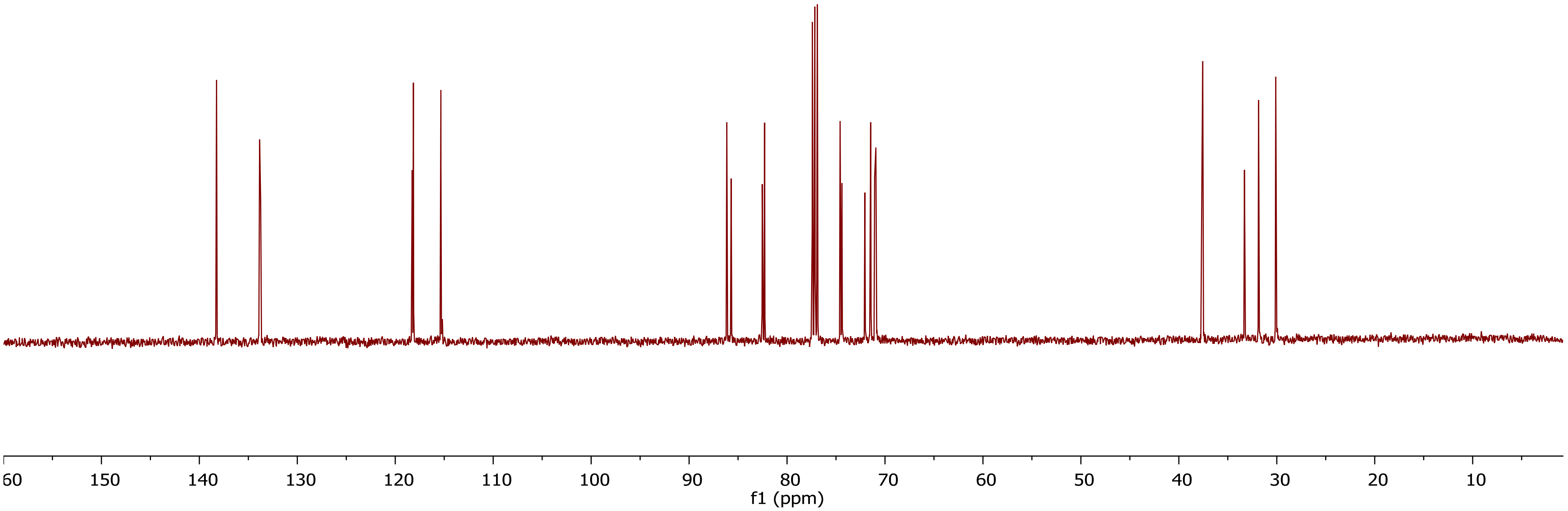

(1*R*)- and (1*S*)-*tert*-butyl({[(2*R*,3*R*,4*S*,5*S*)-4-[(*tert*-butyldiphenylsilyl)oxy]-5-[1-[(*tert*-butyldiphenylsilyl)oxy]pent-4-en-1-yl]-2-(prop-2-en-1-yl)oxolan-3-yl]oxy})diphenylsilane (1d) ^{1}H NMR

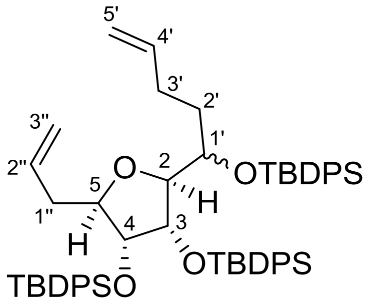

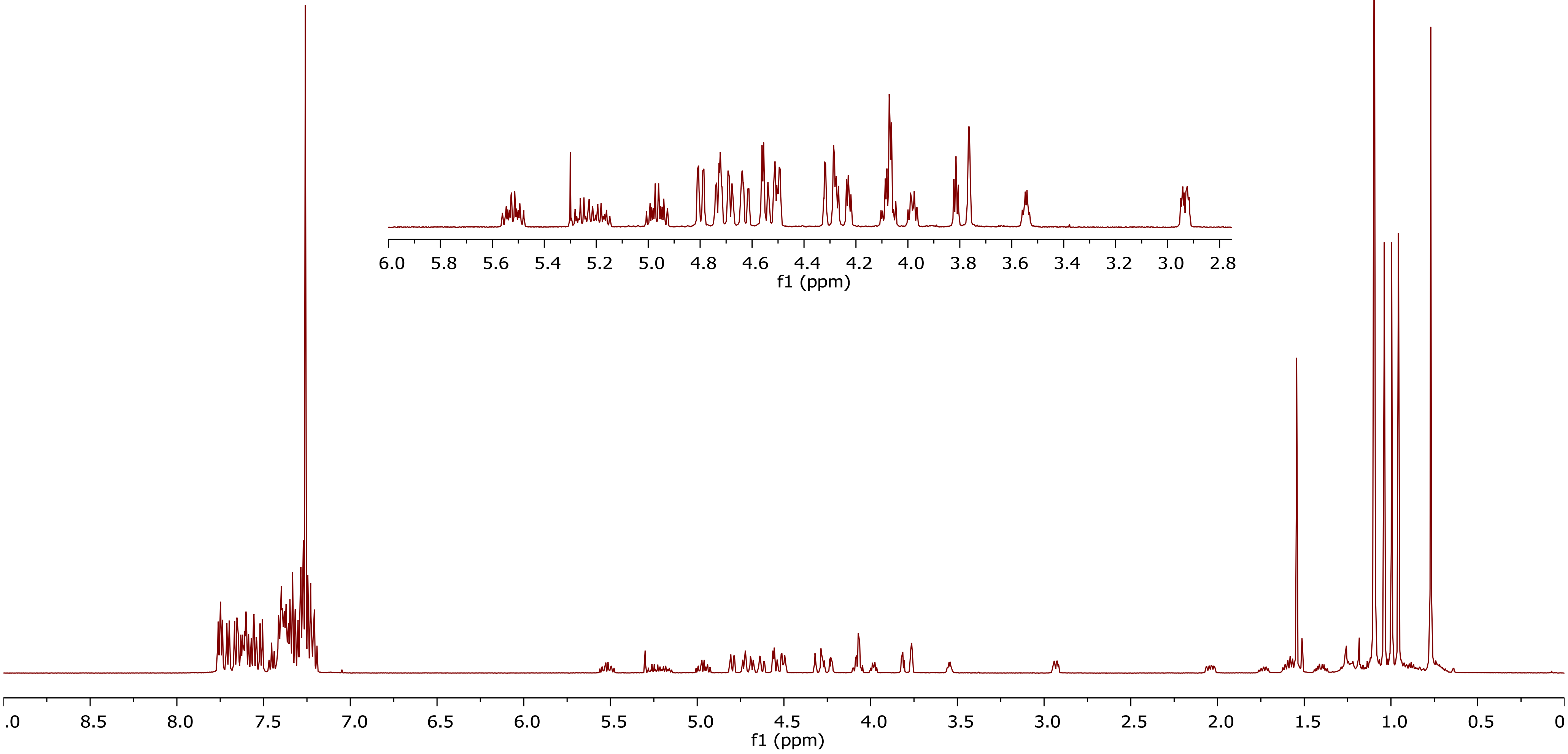

(1*R*)- and (1*S*)-*tert*-butyl({[(2*R*,3*R*,4*S*,5*S*)-4-[(*tert*-butyldiphenylsilyl)oxy]-5-[1-[(*tert*-butyldiphenylsilyl)oxy]pent-4-en-1-yl]-2-(prop-2-en-1-yl)oxolan-3-yl]oxy})diphenylsilane (1d) DEPTq ^{13}C NMR

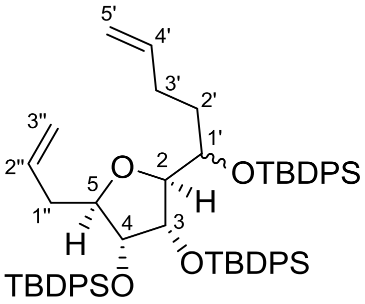

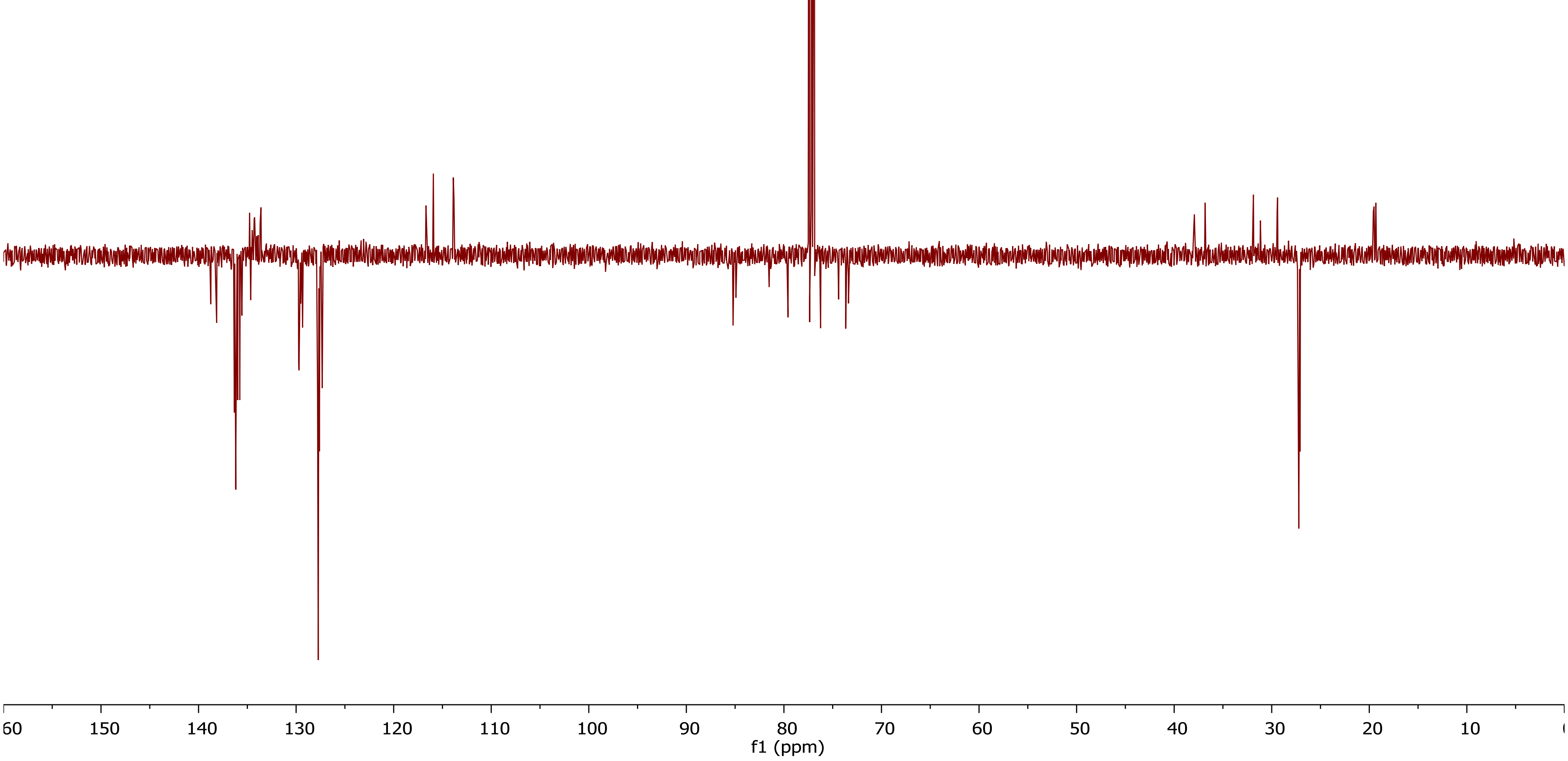

(1*R*)- and (1*S*)-*tert*-butyl({[(2*R*,3*R*,4*S*,5*S*)-4-[(*tert*-butyldimethylsilyl)oxy]-5-[1-[(*tert*-butyldimethylsilyl)oxy]pent-4-en-1-yl]-2-(prop-2-en-1-yl)oxolan-3-yl]oxy})dimethylsilane (1e) ^{1}H NMR

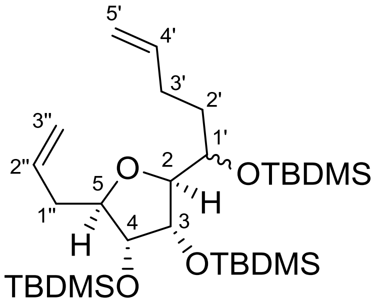

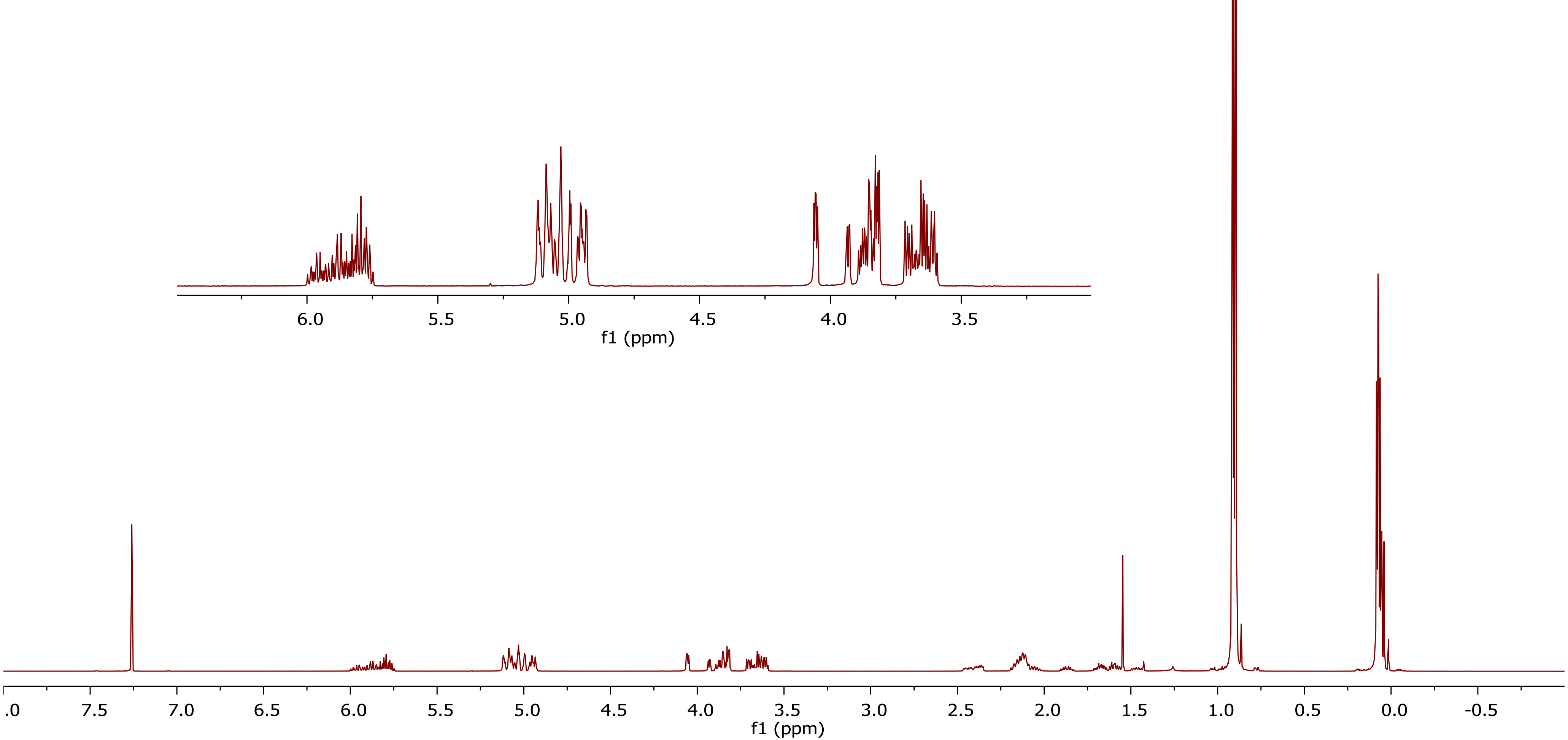

(1*R*)- and (1*S*)-*tert*-butyl({[(2*R*,3*R*,4*S*,5*S*)-4-[(*tert*-butyldimethylsilyl)oxy]-5-[1-[(*tert*-butyldimethylsilyl)oxy]pent-4-en-1-yl]-2-(prop-2-en-1-yl)oxolan-3-yl]oxy})dimethylsilane (1e) DEPTq ^{13}C NMR

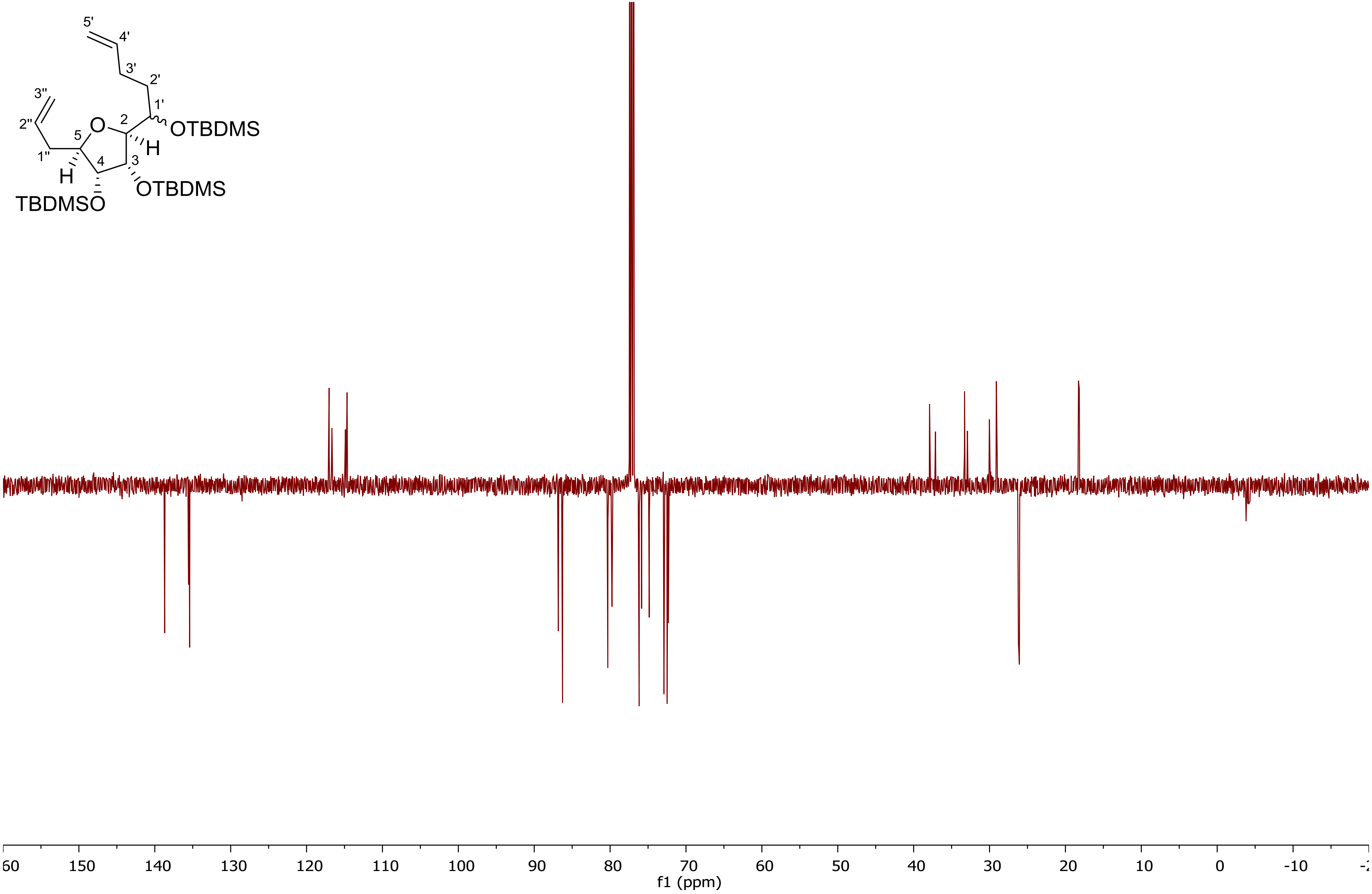

(1*S*)- and (1*R*)-trimethyl({[(2*S*,3*S*,4*R*,5*R*)-5-(prop-2-en-1-yl)-4-[(trimethylsilyl)oxy]-5-[1-[(trimethylsilyl)oxy]pent-4-en-1-yl]oxolan-3-yl]oxy})silane (1f) ^{1}H NMR

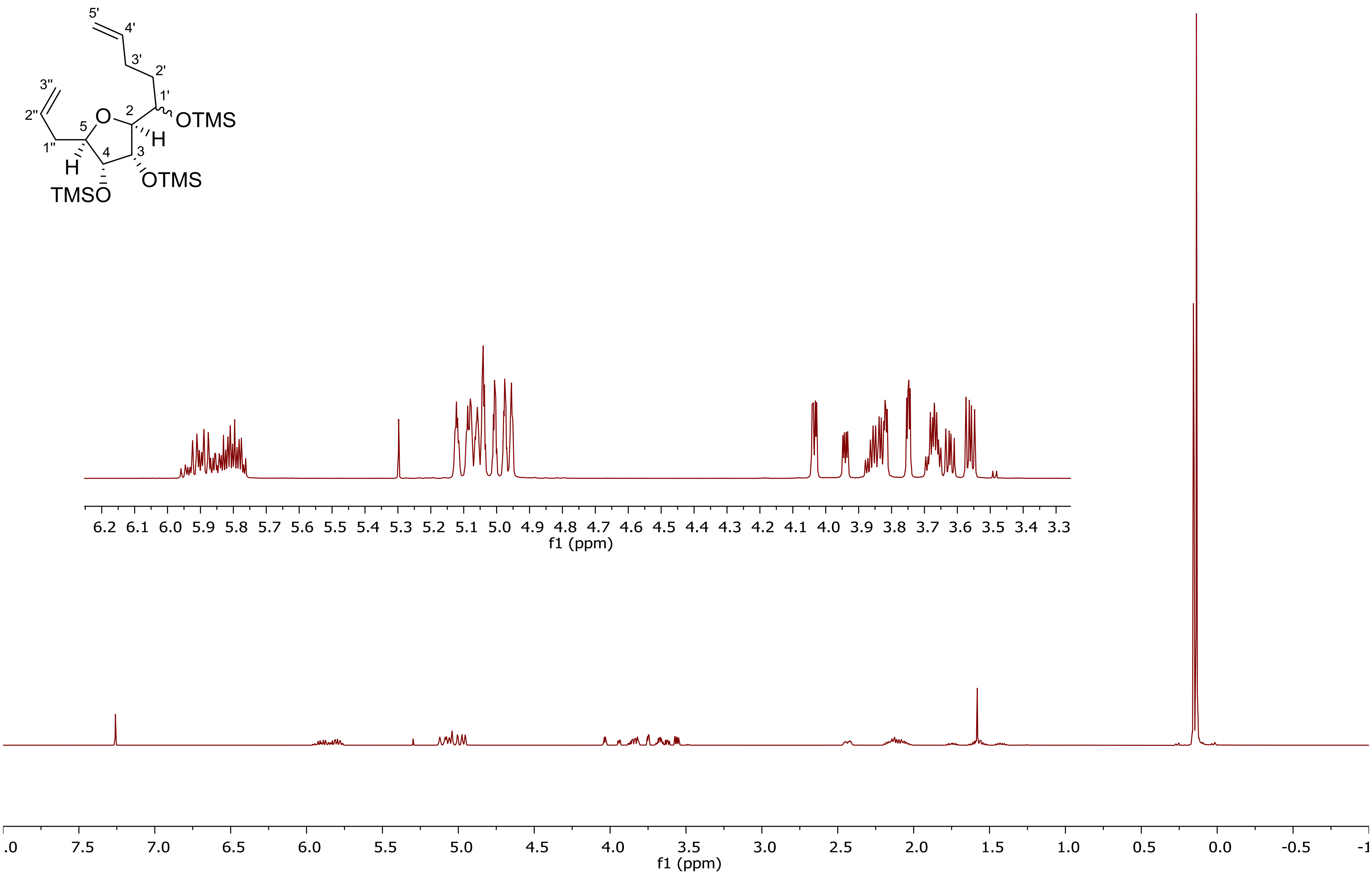

(1*S*)- and (1*R*)-trimethyl({[(2*S*,3*S*,4*R*,5*R*)-5-(prop-2-en-1-yl)-4-[(trimethylsilyl)oxy]-5-[1-[(trimethylsilyl)oxy]pent-4-en-1-yl]oxolan-3-yl]oxy})silane (1f) DEPTq ^{13}C NMR

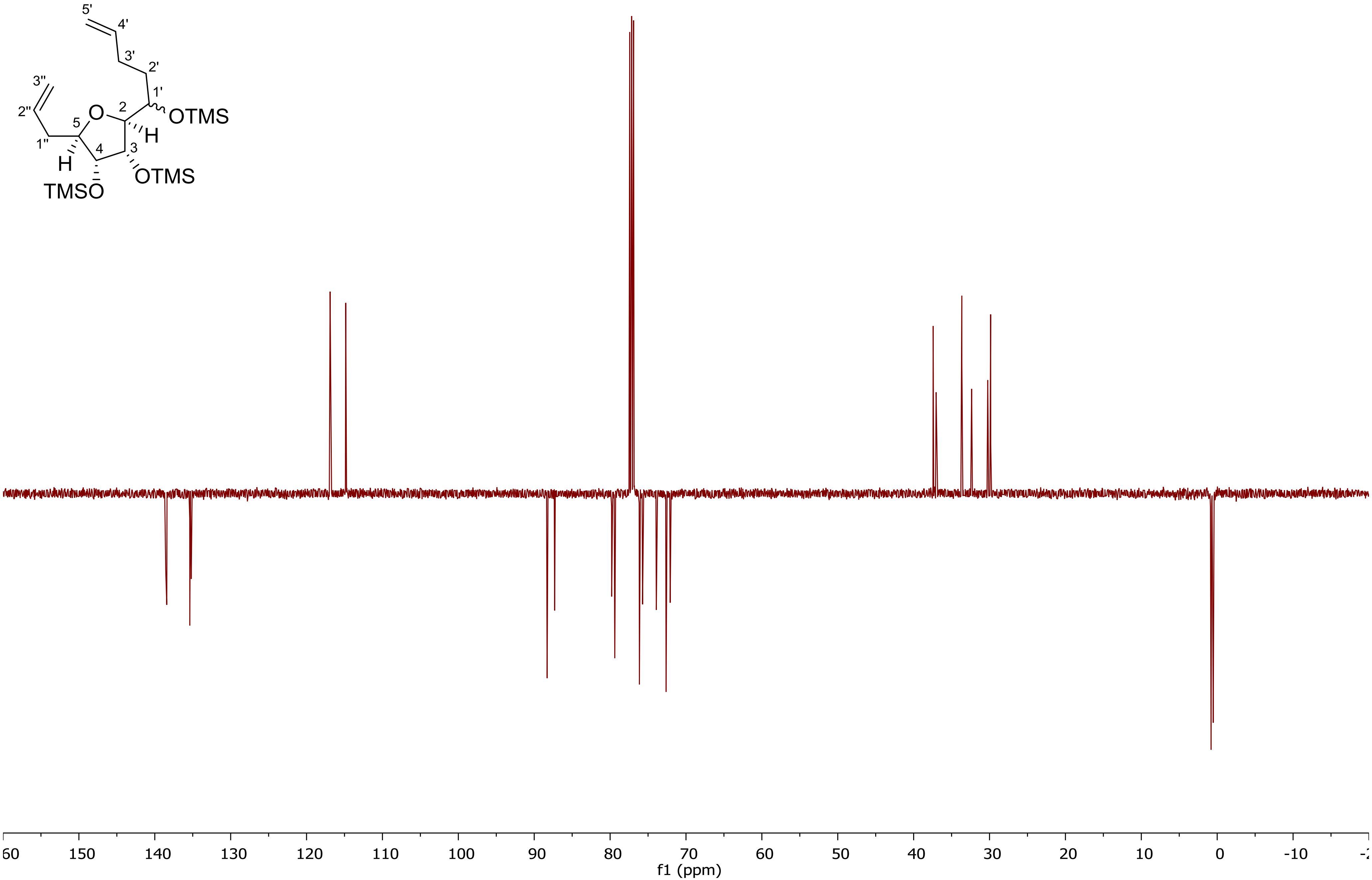

{[(1*S*)- and (1*R*)-1-[(3a*S*,4*R*,6*R*,6a*R*)-2,2-dimethyl-6-(prop-2-en-1-yl)-tetrahydro-2H-furo[3,4-*d*][1,3]dioxol-4-yl]pent-4-en-1-yl]oxy}(*tert*-butyl)dimethylsilane (1g) ^{1}H NMR

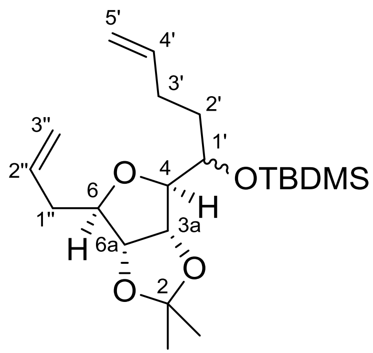

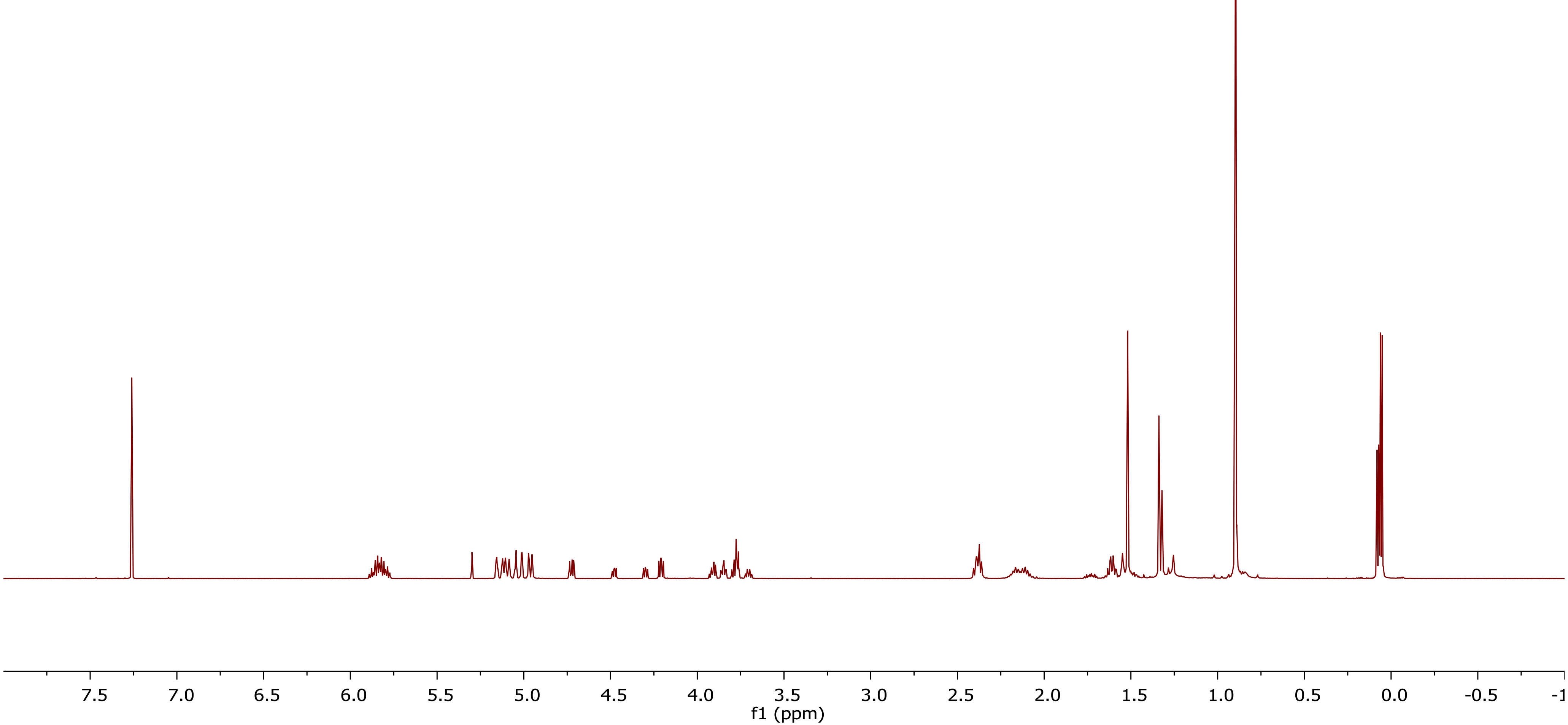

{[(1*S*)- and (1*R*)-1-[(3a*S*,4*R*,6*R*,6a*R*)-2,2-dimethyl-6-(prop-2-en-1-yl)-tetrahydro-2H-furo[3,4-*d*][1,3]dioxol-4-yl]pent-4-en-1-yl]oxy}(*tert*-butyl)dimethylsilane (1g) DEPTq ^{13}C NMR

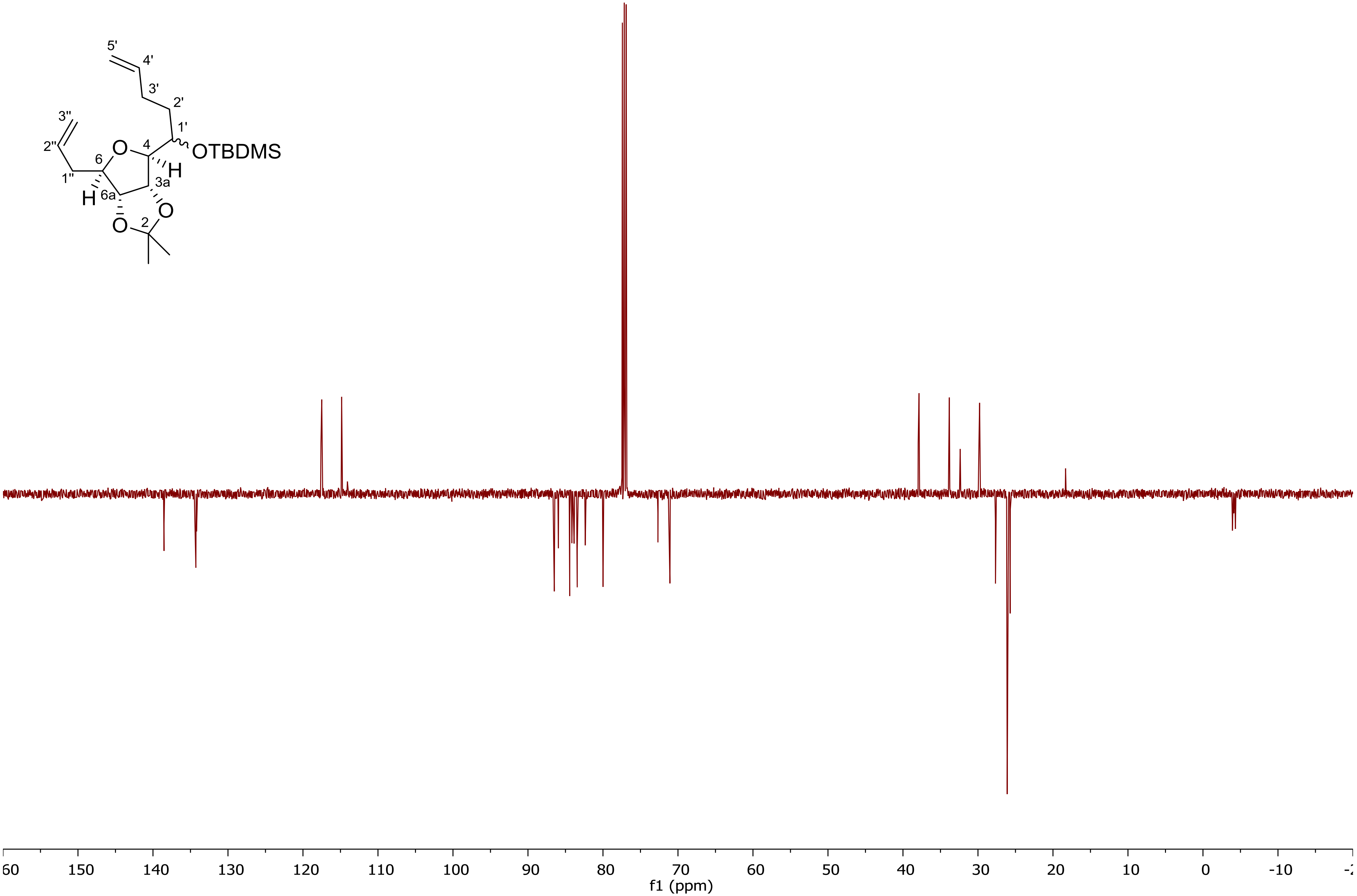

(1*R*)- and (1*S*)-({1-[(3a*S*,4*R*,6*R*,6a*R*)-2,2-dimethyl-6-(prop-2-en-1-yl)-tetrahydro-2H-furo[3,4-*d*][1,3]dioxol-4-yl]- pent-4-en-1-yl}oxy)(*tert*-butyl)diphenylsilane (1h) ^{1}H NMR

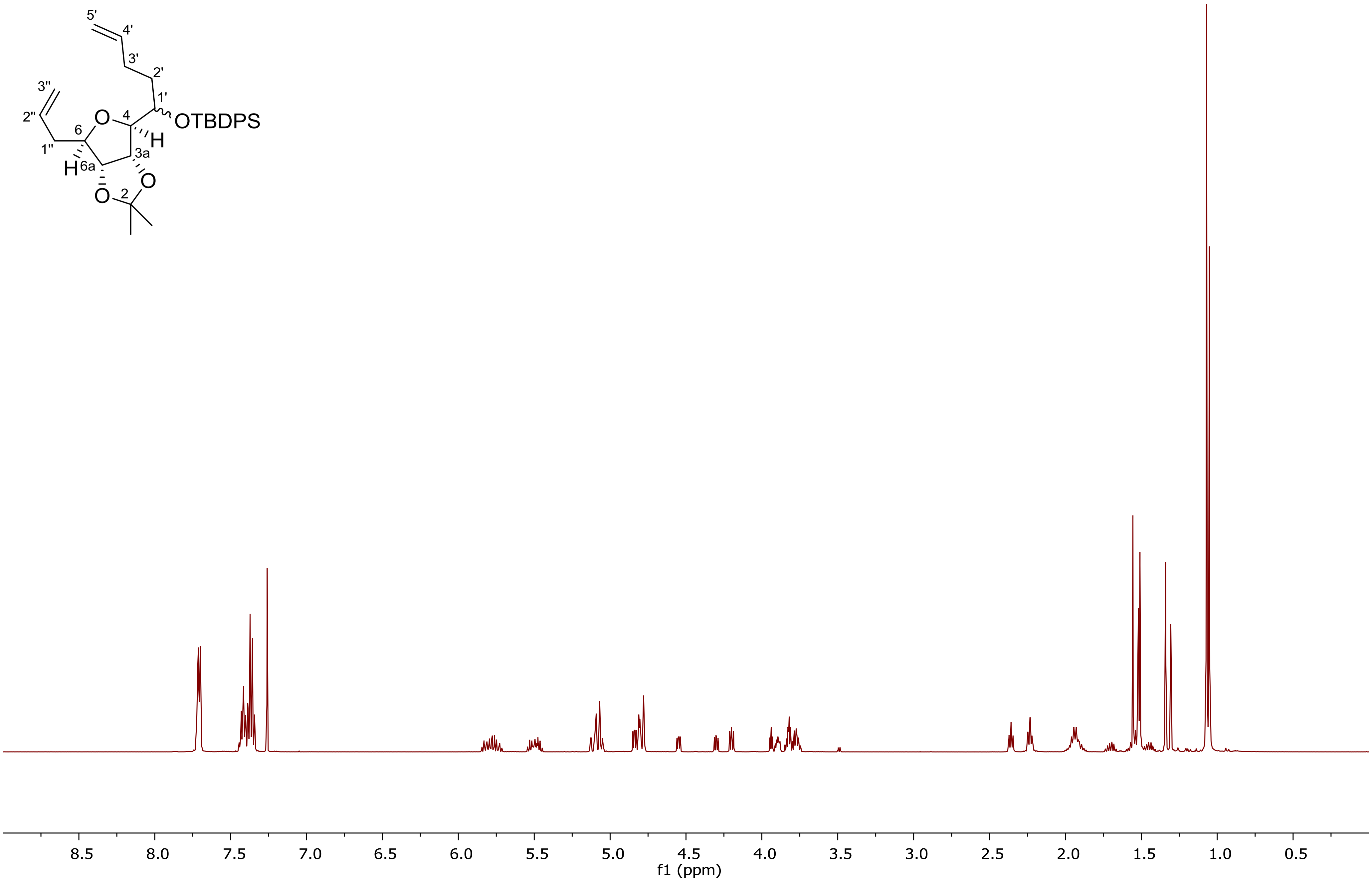

(1*R*)- and (1*S*)-({1-[(3a*S*,4*R*,6*R*,6a*R*)-2,2-dimethyl-6-(prop-2-en-1-yl)-tetrahydro-2H-furo[3,4-*d*][1,3]dioxol-4-yl]- pent-4-en-1-yl}oxy)(*tert*-butyl)diphenylsilane (1h) DEPTq ^{13}C NMR

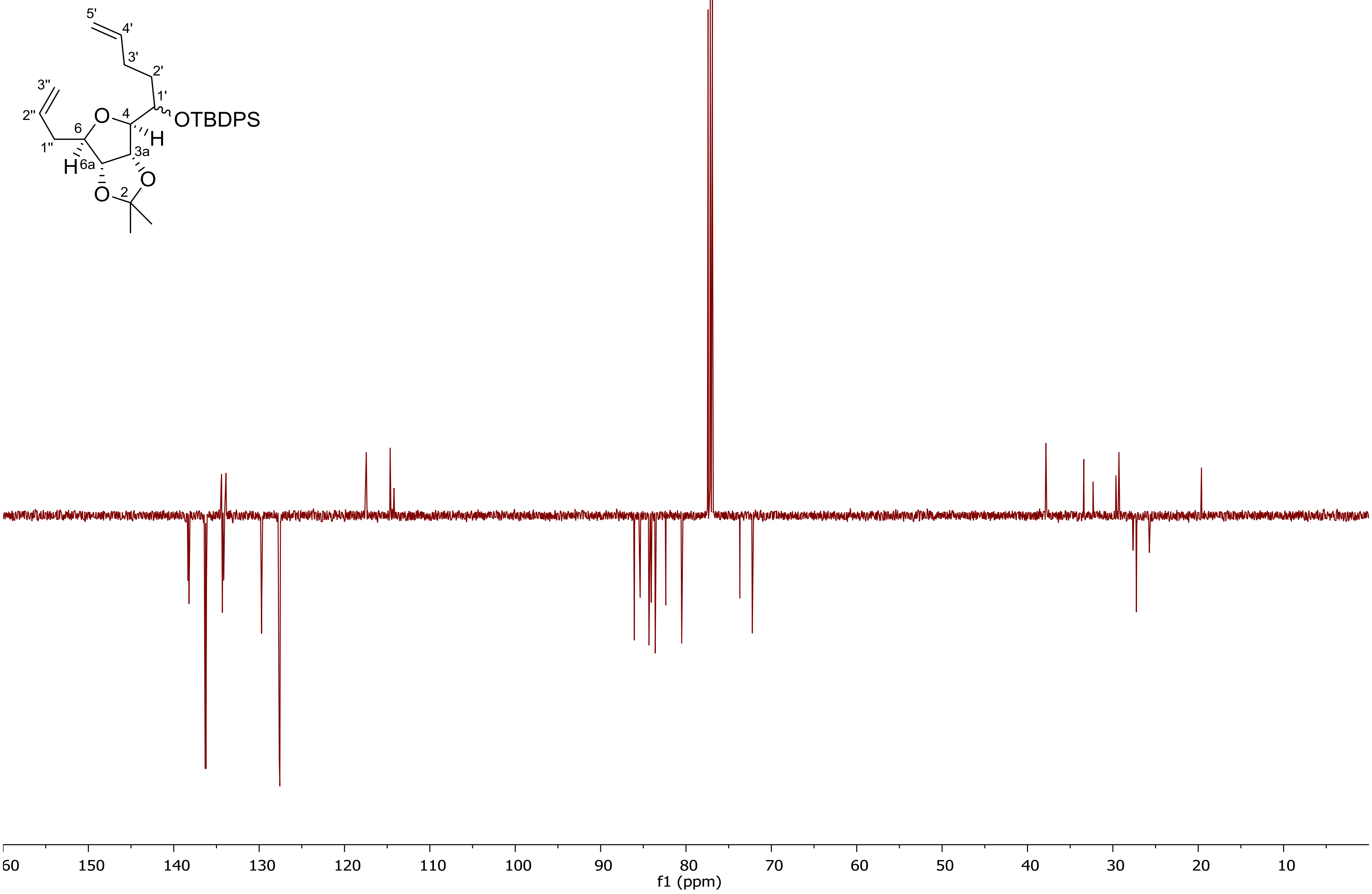

(1*R*)- and (1*S*)- (2*R*,3*R*,4*S*,5*R*)-2-[1-[(*tert*-butyldiphenylsilyl)oxy]pent-4-en-1-yl]-5-(prop-2-en-1-yl)oxolane-3,4-diol ^{1}H NMR

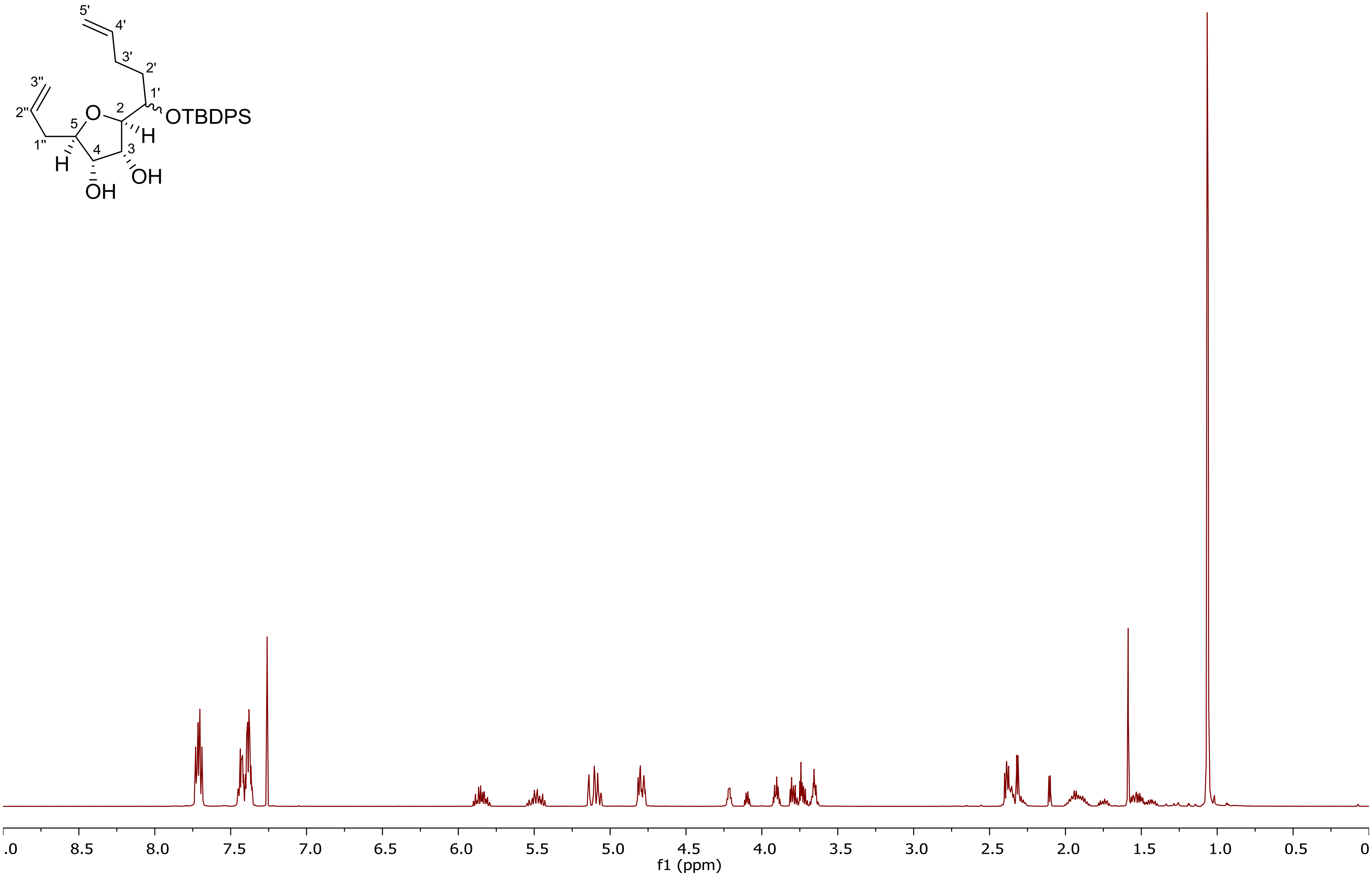

(1*R*)- and (1*S*)- (2*R*,3*R*,4*S*,5*R*)-2-[1-[(*tert*-butyldiphenylsilyl)oxy]pent-4-en-1-yl]-5-(prop-2-en-1-yl)oxolane-3,4-diol DEPTq ^{13}C NMR

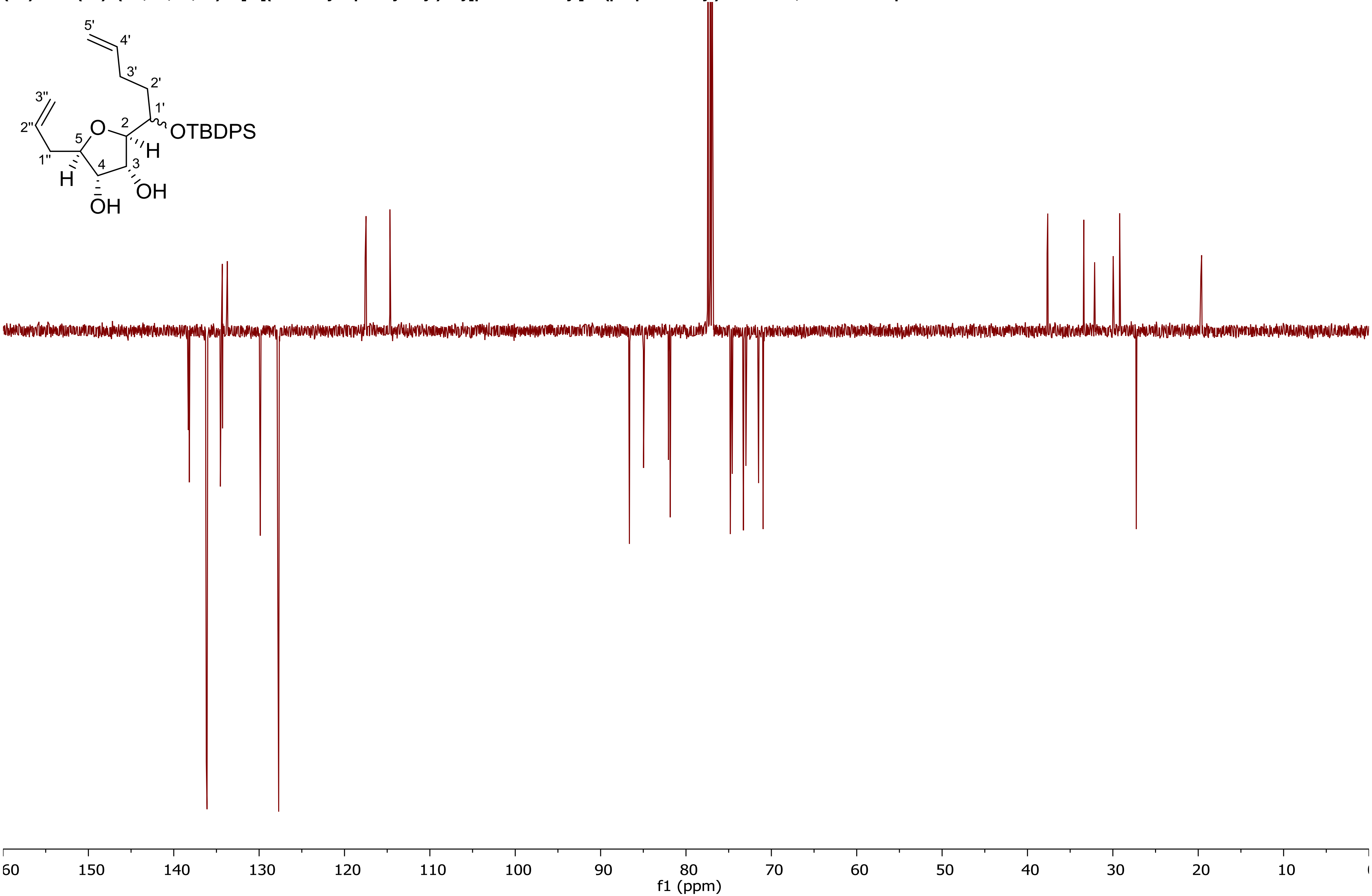

(1*R*)- and (1*S*)-{[1-[(2*R*,3*R*,4*R*,5*R*)-3,4-bis(benzyloxy)-5-(prop-2-en-1-yl)oxolan-2-yl]pent-4-en-1-yl]oxy}(*tert*-butyl)diphenylsilane (1i) ^{1}H NMR

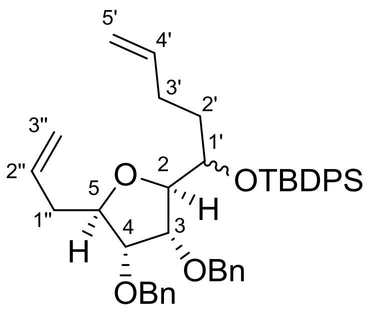

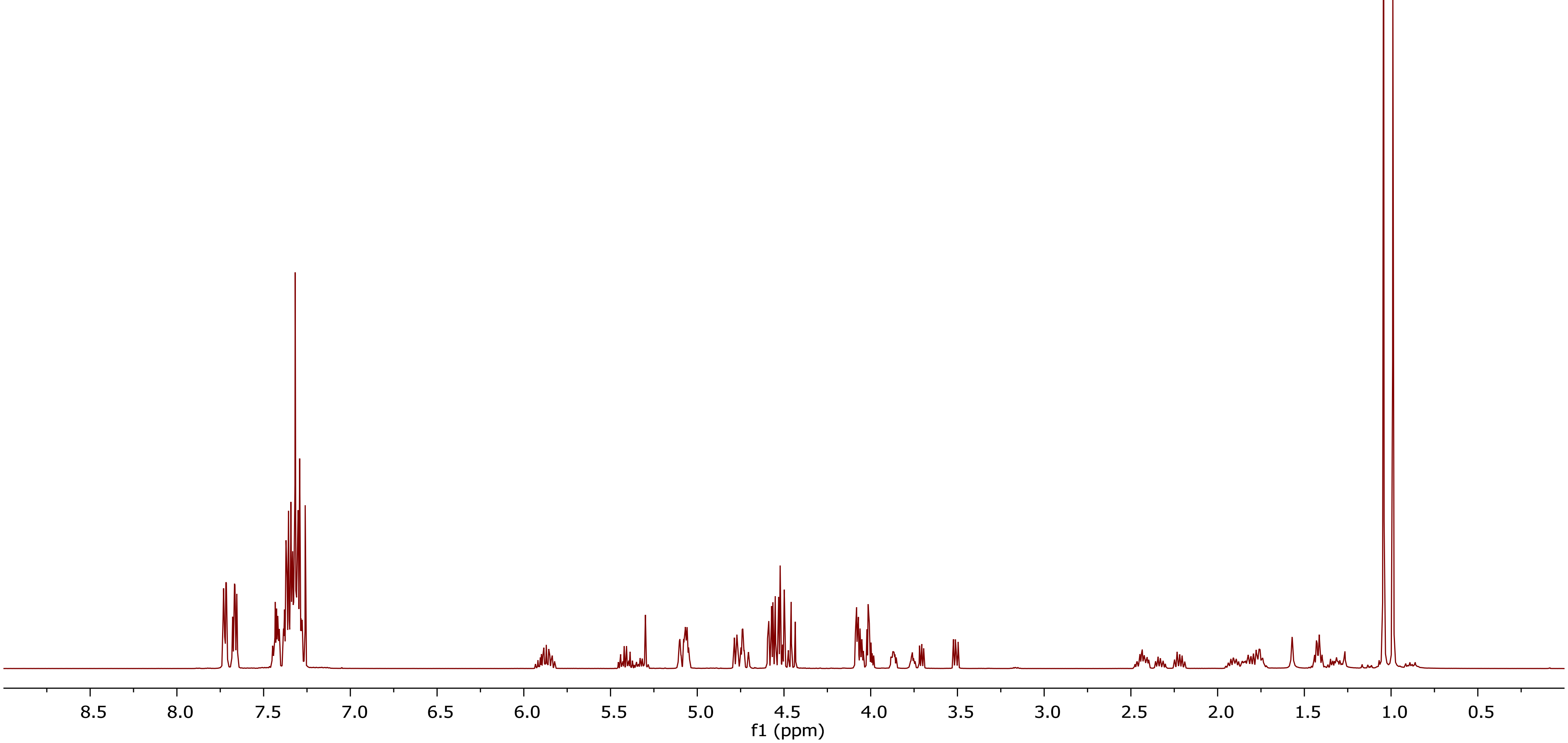

(1*R*)- and (1*S*)-{[1-[(2*R*,3*R*,4*R*,5*R*)-3,4-bis(benzyloxy)-5-(prop-2-en-1-yl)oxolan-2-yl]pent-4-en-1-yl]oxy}(*tert*-butyl)diphenylsilane (1i) DEPTq ^{13}C NMR

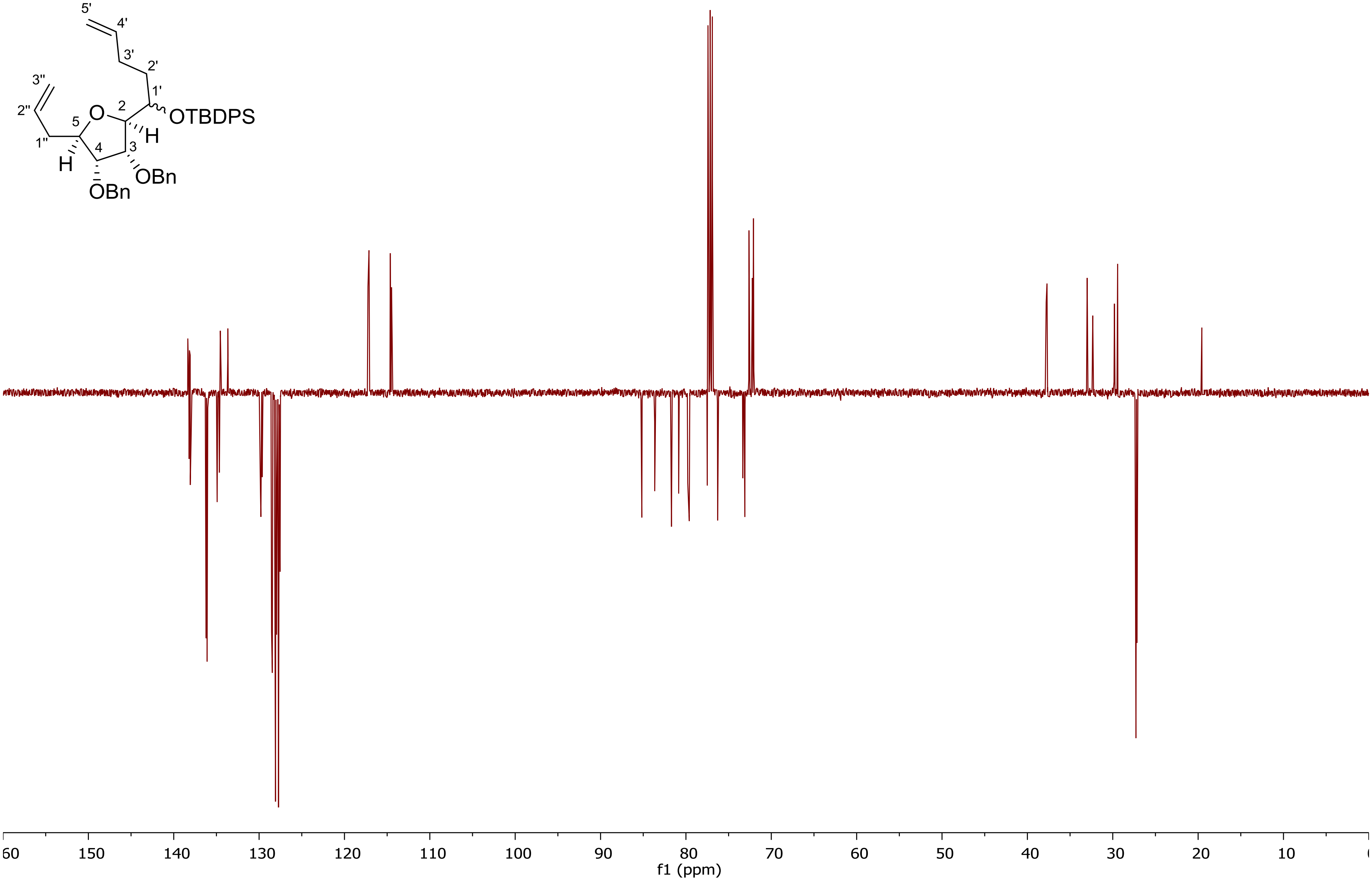

(1*R*)- and (1*S*)-{[1-[(3a*S*,4*S*,6*R*,6a*R*)-2,2-di-*tert*-butyl-6-(prop-2-en-1-yl)-tetrahydro-2H-furo[3,4-*d*][1,3,2]dioxasilol-4-yl]pent-4-en-1-yl]oxy}(*tert*-butyl)diphenylsilane (1j) ^{1}H NMR

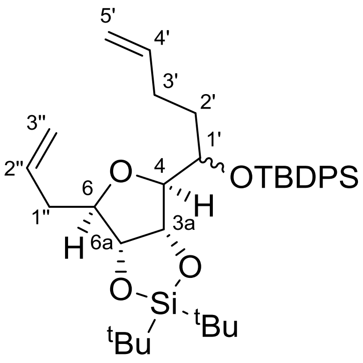

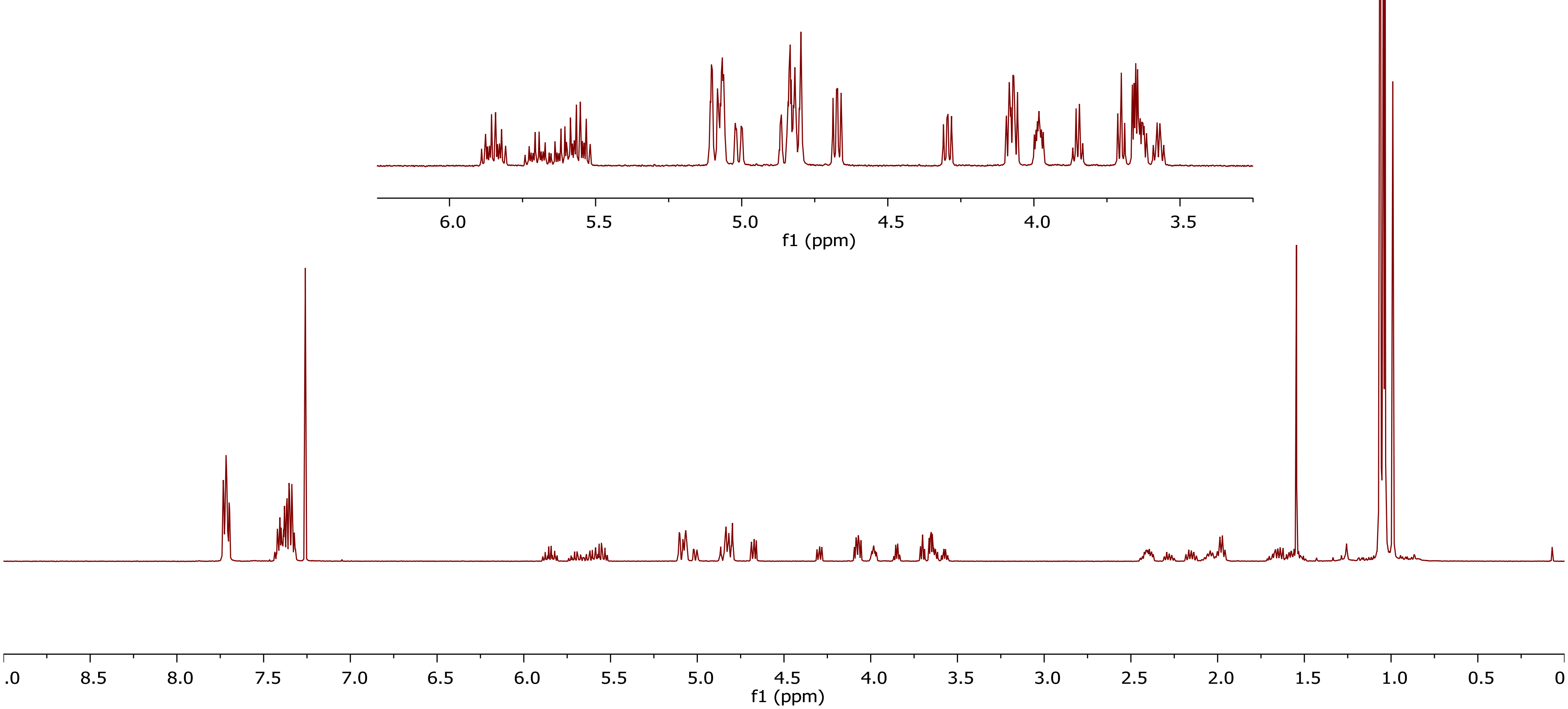

(1*R*)- and (1*S*)-{[1-[(3a*S*,4*S*,6*R*,6a*R*)-2,2-di-*tert*-butyl-6-(prop-2-en-1-yl)-tetrahydro-2H-furo[3,4-*d*][1,3,2]dioxasilol-4-yl]pent-4-en-1-yl]oxy}(*tert*-butyl)diphenylsilane (1j) DEPTq ^{13}C NMR

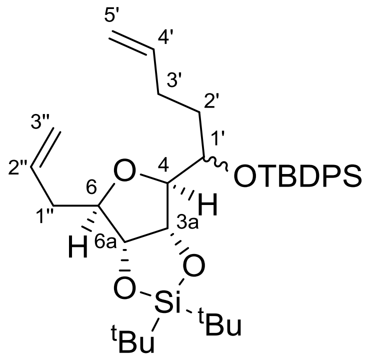

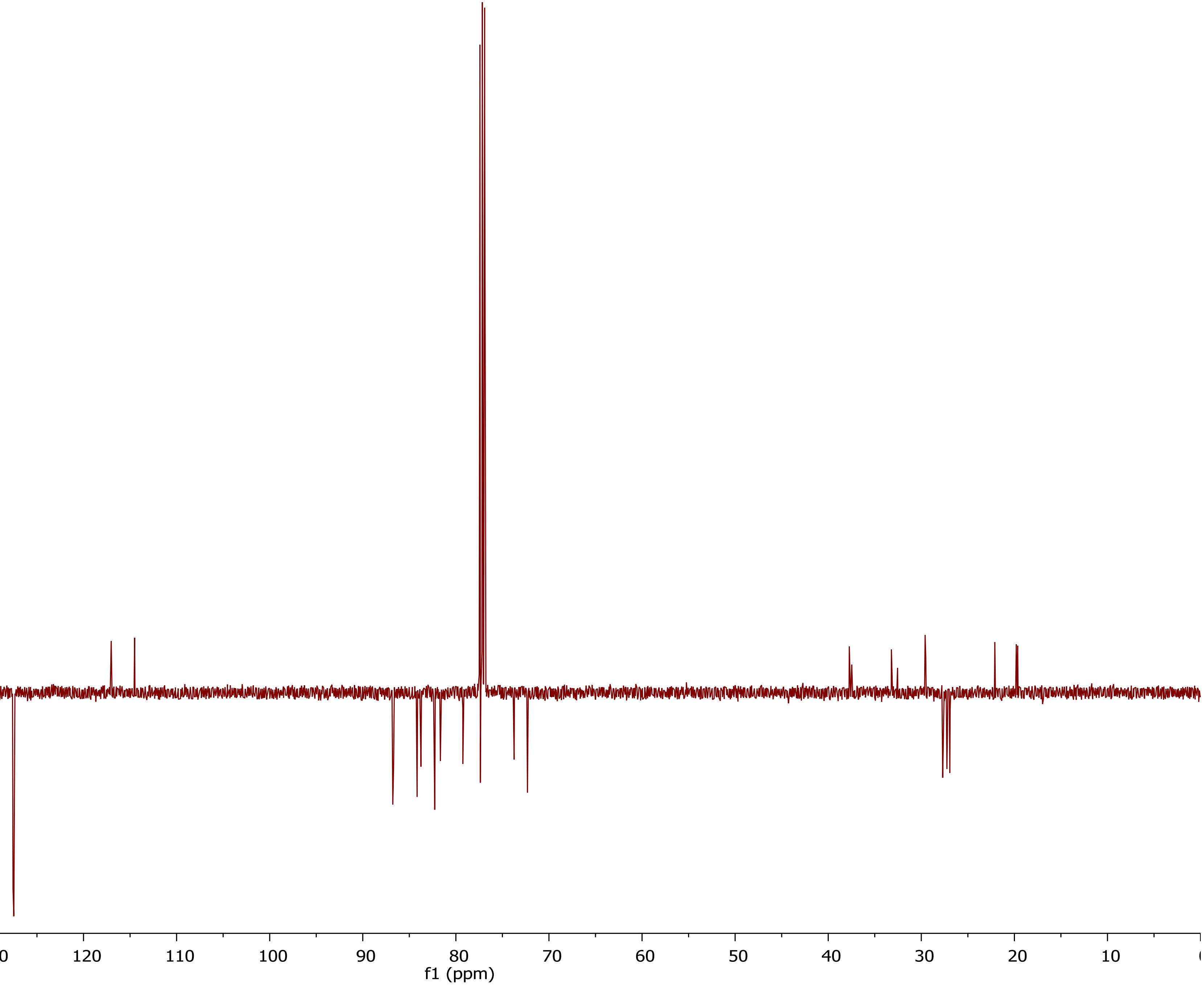

(1*R*,3*Z*,7*S*,8*S*,9*S*,13*R*)-11,11-dimethyl-7-(naphthalen-2-ylmethoxy)-10,12,14-trioxatricyclo[6.5.1.0^{9,13}]tetradec-3-ene (10a) ^{1}H NMR

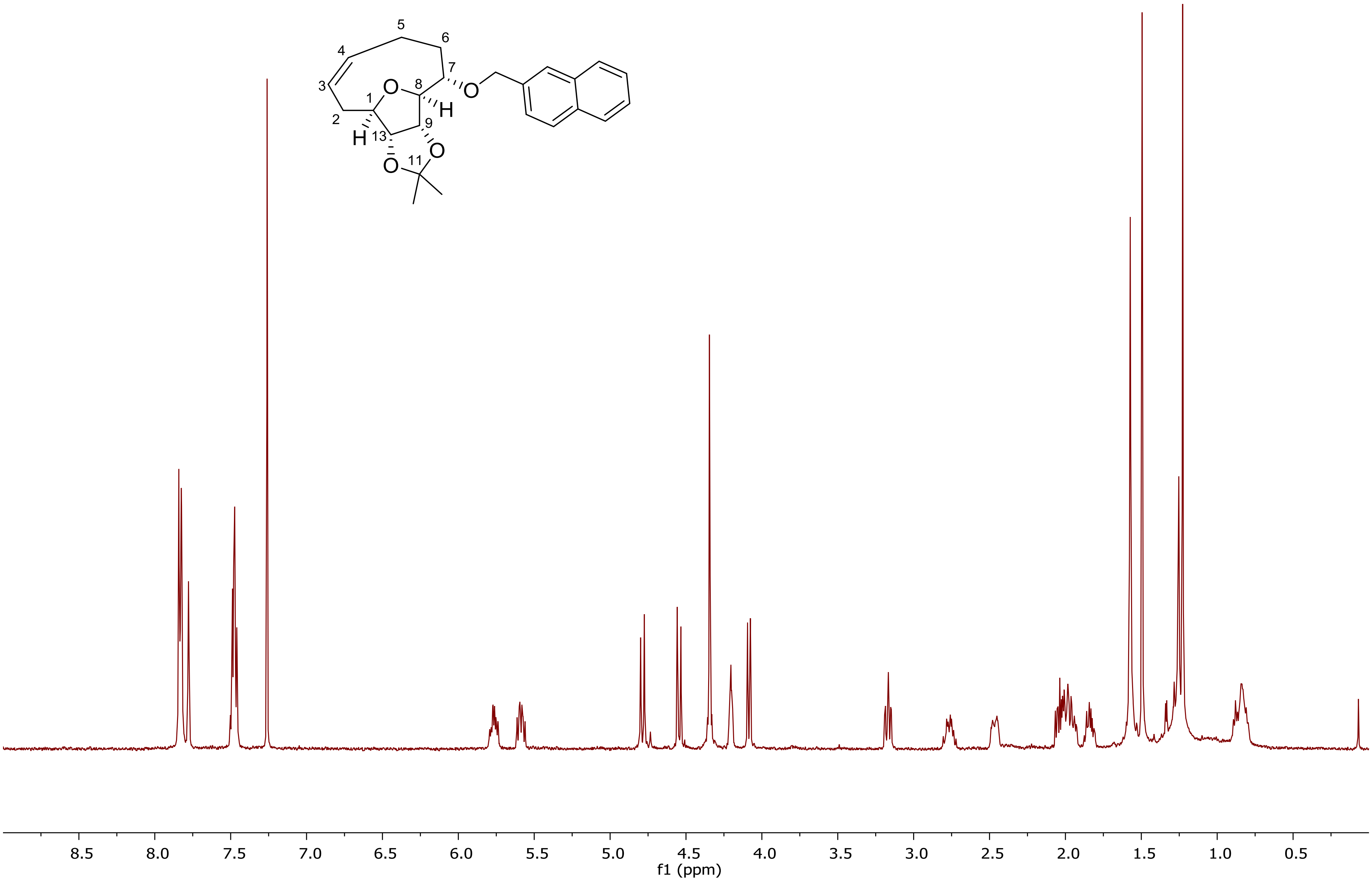

(1*R*,3*Z*,7*S*,8*S*,9*S*,13*R*)-11,11-dimethyl-7-(naphthalen-2-ylmethoxy)-10,12,14-trioxatricyclo[6.5.1.0^{9},^{13}]tetradec-3-ene (10a) UDEFT ^{13}C NMR

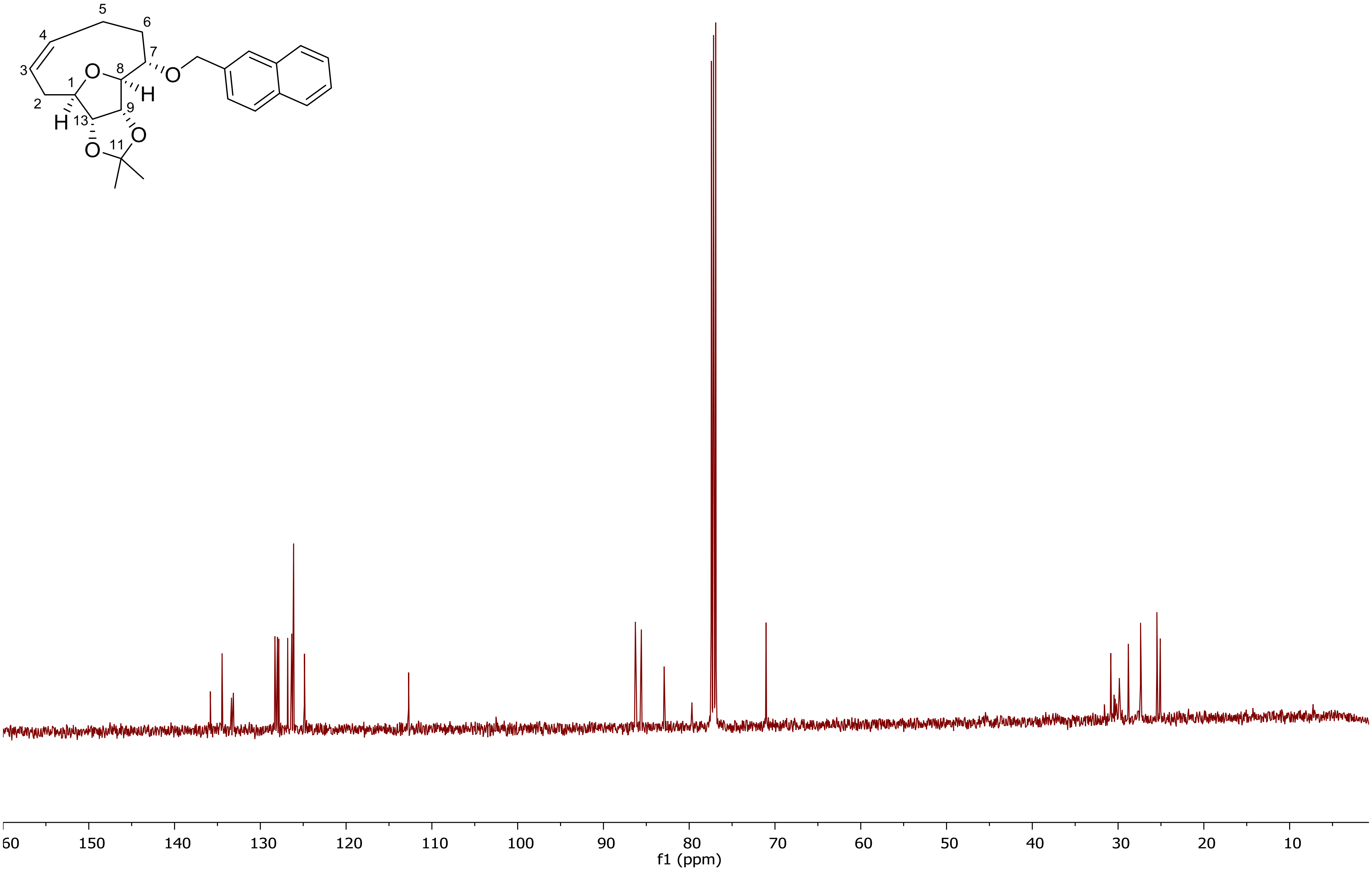

(7*R*)- and (7*S*)-{[(1*R*,3*Z*,8*S*,9*S*,10*R*)-7,10-bis[(*tert*-butyldiphenylsilyl)oxy]-11-oxabicyclo[6.2.1]undec-3-en-9-yl]oxy}(*tert*-butyl)diphenylsilane (10d) ^{1}H NMR

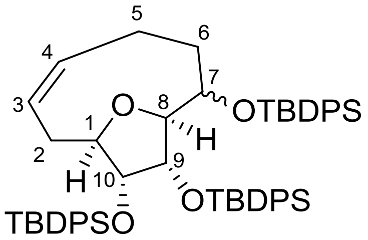

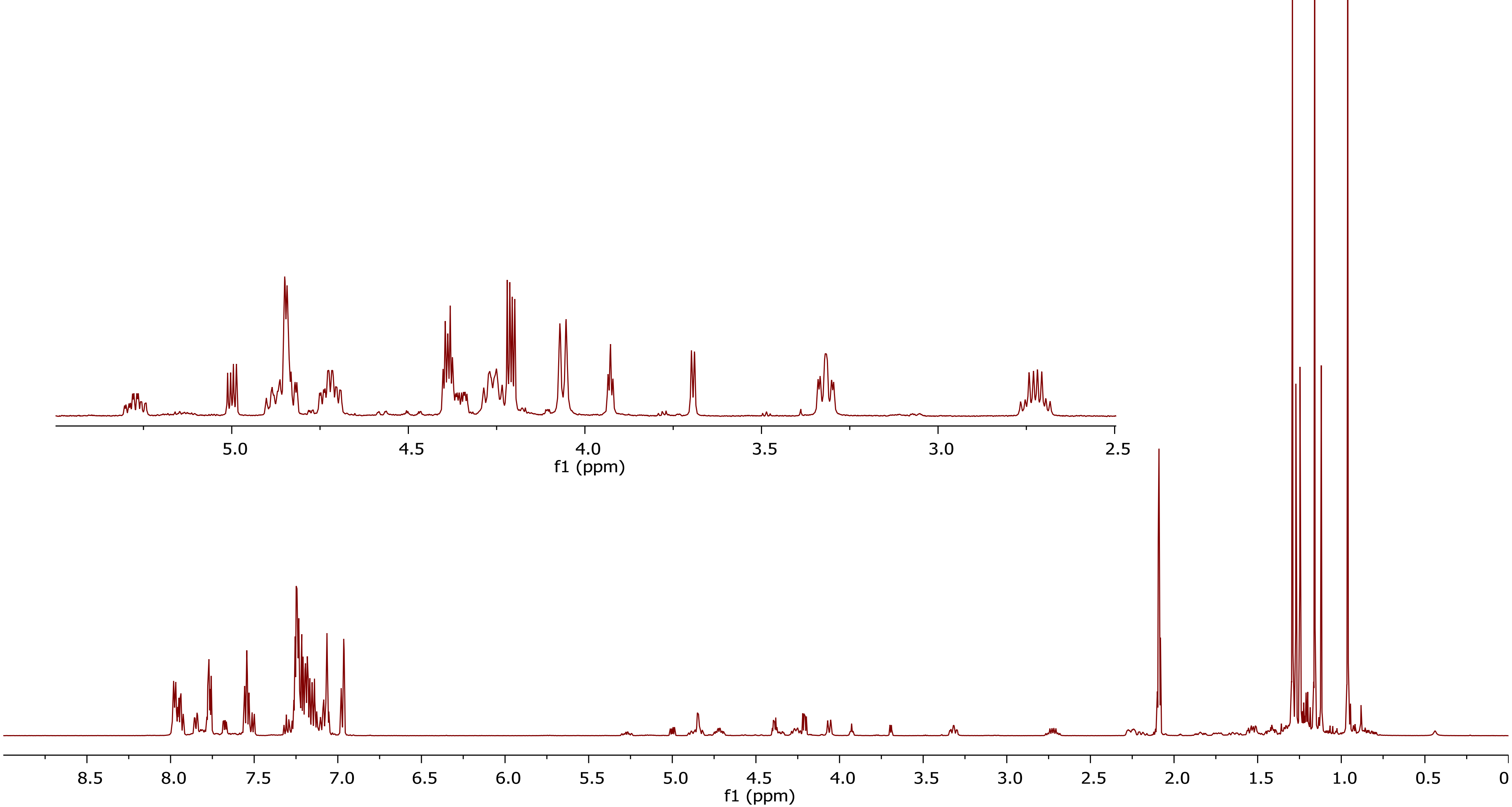

(7*R*)- and (7*S*)-{[(1*R*,3*Z*,8*S*,9*S*,10*R*)-7,10-bis[(*tert*-butyldiphenylsilyl)oxy]-11-oxabicyclo[6.2.1]undec-3-en-9-yl]oxy}(*tert*-butyl)diphenylsilane (10d) DEPTq ^{13}C NMR

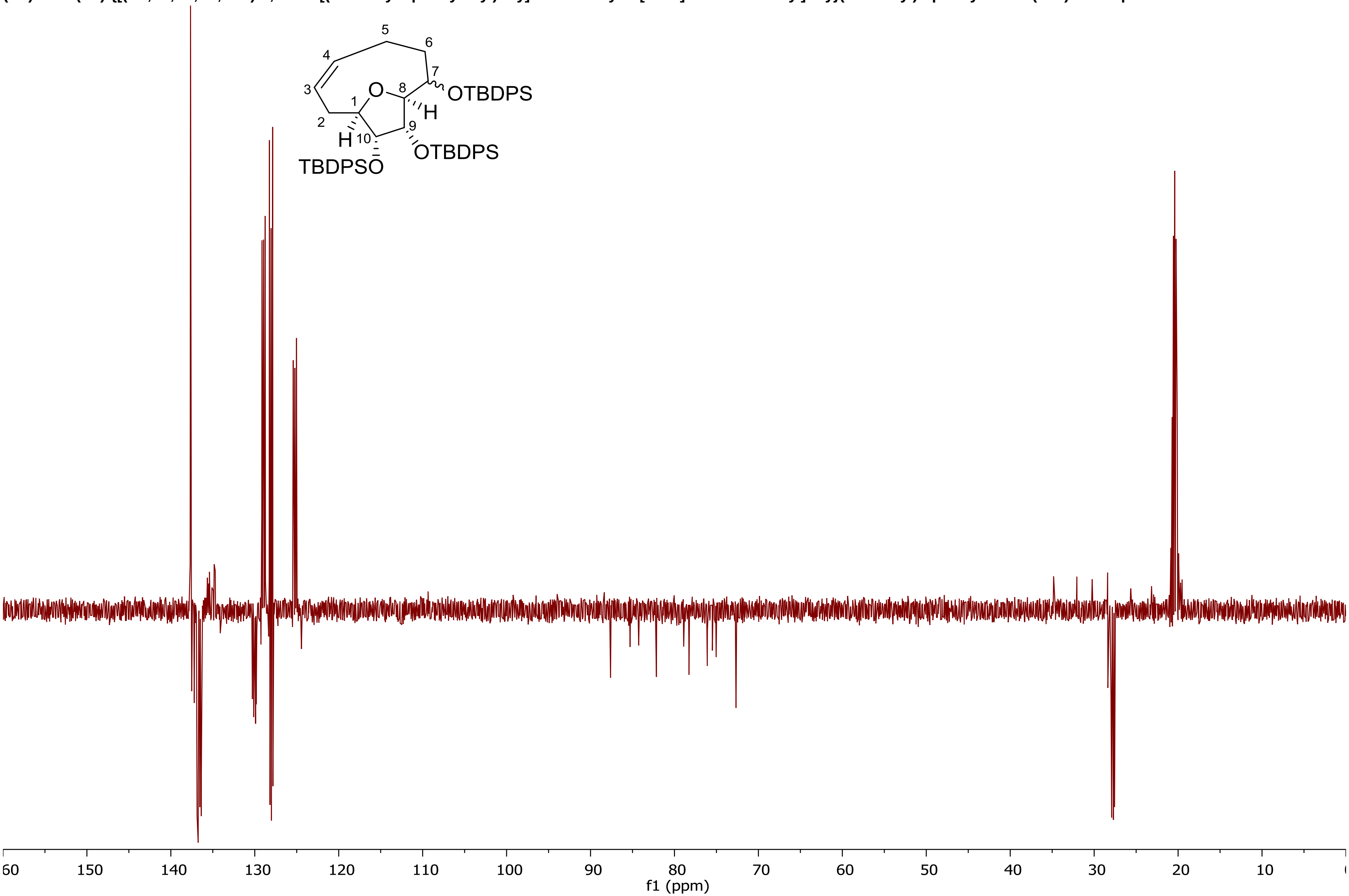

{[(1*S*,2*S*,5*Z*,8*R*,9*R*,10*S*)-2,10-bis[(*tert*-butyldimethylsilyl)oxy]-11-oxabicyclo[6.2.1]undec-5-en-9-yl]oxy}(*tert*-butyl)dimethylsilane (10e) ^{1}H NMR

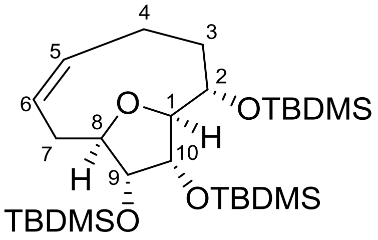

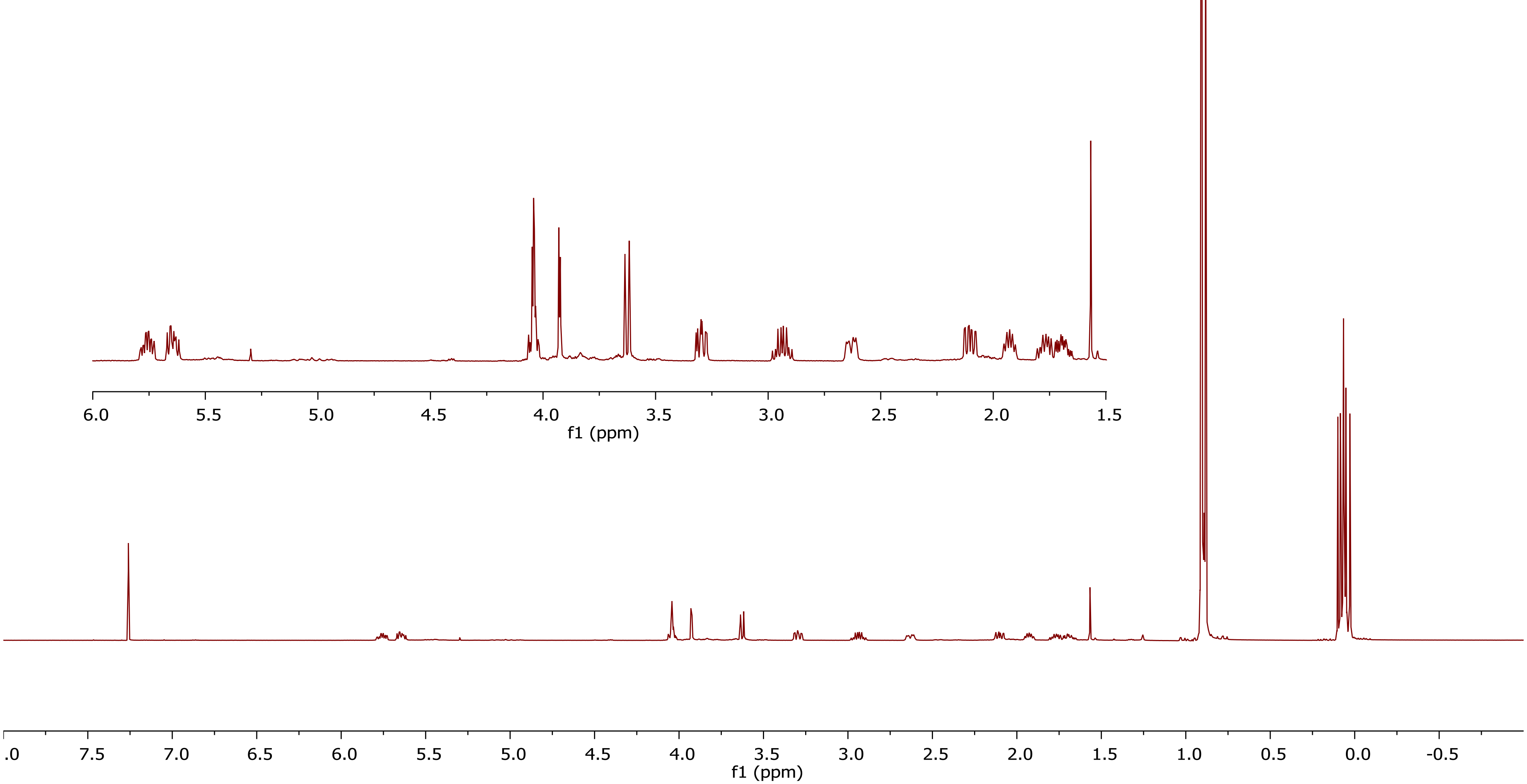

{[(1*S*,2*S*,5*Z*,8*R*,9*R*,10*S*)-2,10-bis[(*tert*-butyldimethylsilyl)oxy]-11-oxabicyclo[6.2.1]undec-5-en-9-yl]oxy}(*tert*-butyl)dimethylsilane (10e) DEPTq ^{13}C NMR

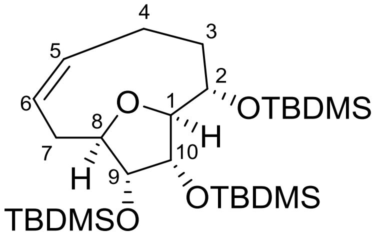

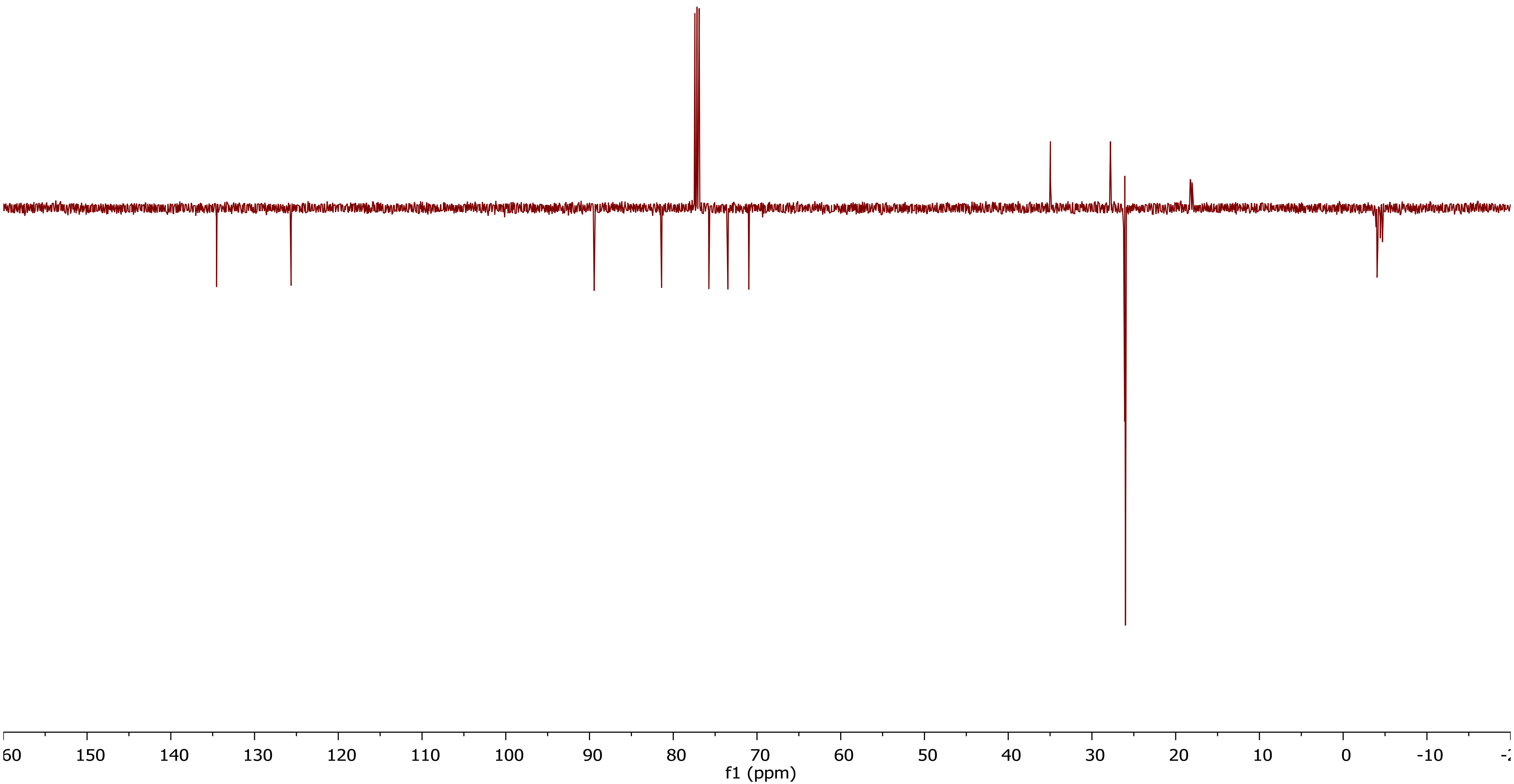

(1*R*,3*Z*,7*S*,8*S*,9*S*,13*R*)-11,11-di-*tert*-butyl-7-[(*tert*-butyldiphenylsilyl)oxy]-10,12,14-trioxa-11-silatricyclo[6.5.1.0^{9,13}]tetradec-3-ene (10j) ^{1}H NMR

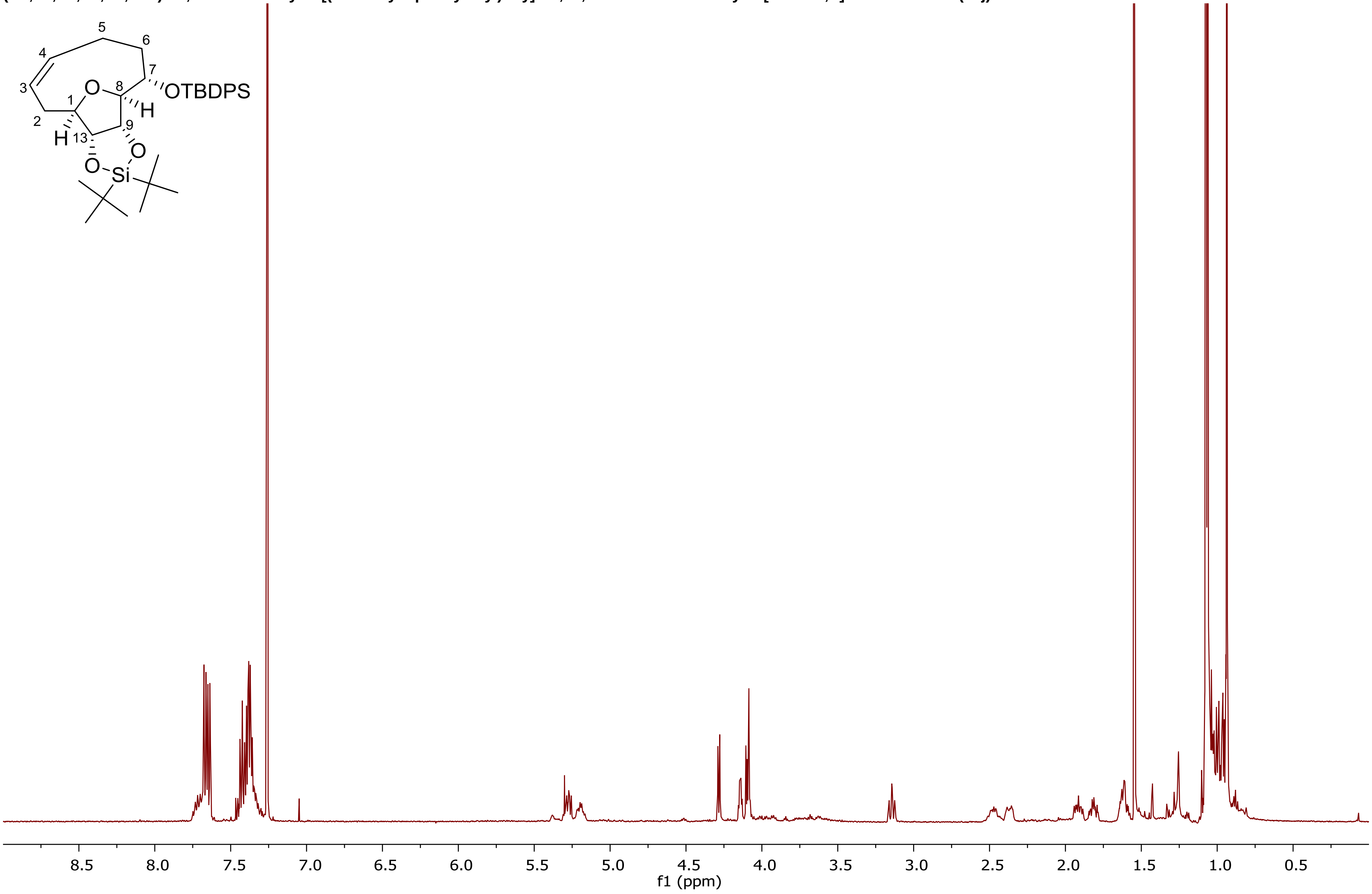

(1*R*,3*Z*,7*S*,8*S*,9*S*,13*R*)-11,11-di-*tert*-butyl-7-[(*tert*-butyldiphenylsilyl)oxy]-10,12,14-trioxa-11-silatricyclo[6.5.1.0^{9},^{13}]tetradec-3-ene (10j) UDEFT ^{13}C NMR

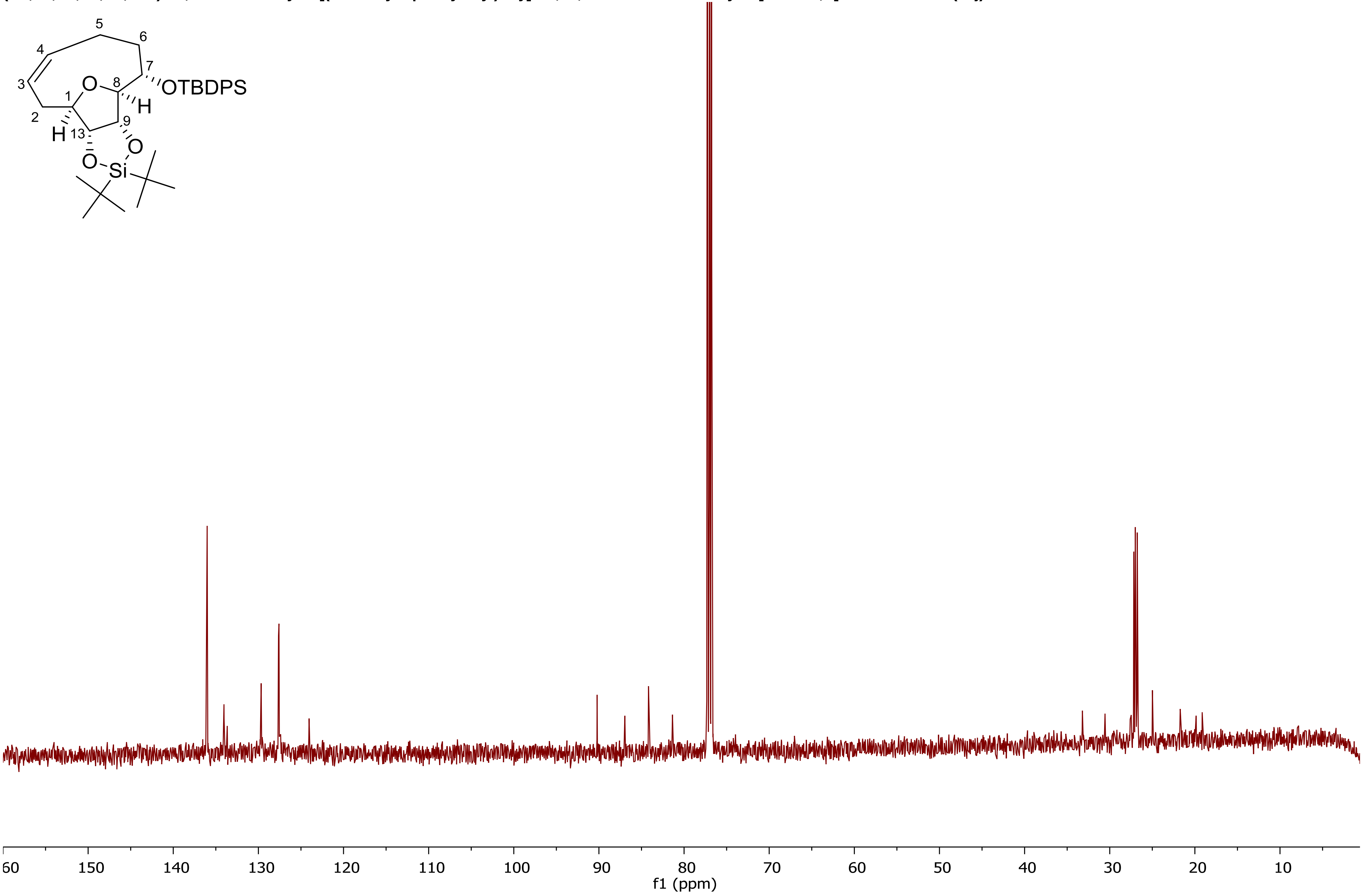

(1*S*,2*S*,5*Z*,8*R*,9*S*,10*R*)-11-oxabicyclo[6.2.1]undec-5-ene-2,9,10-triol (2) ^{1}H NMR

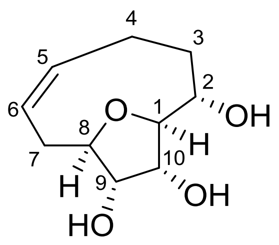

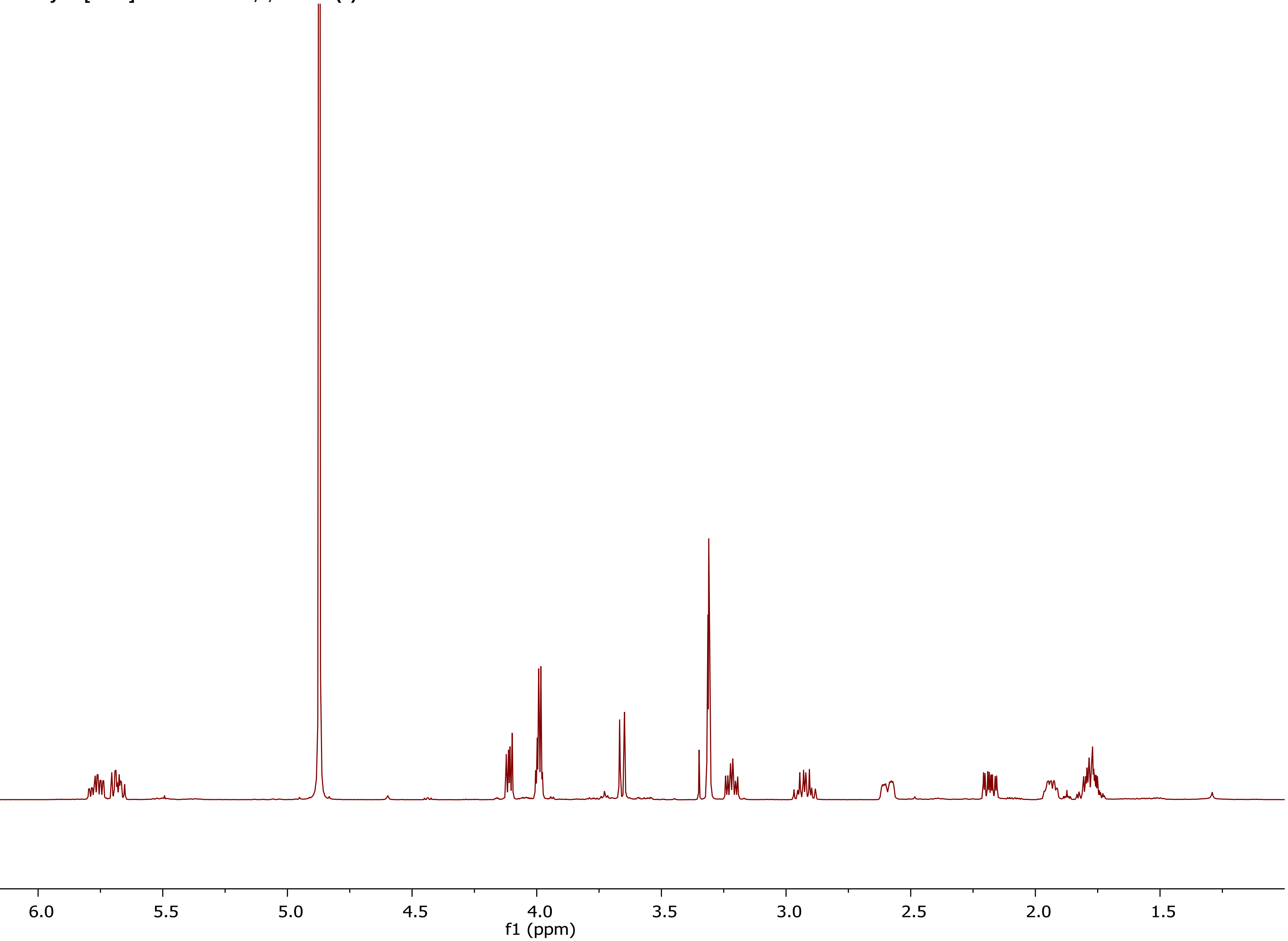

(1*S*,2*S*,5*Z*,8*R*,9*S*,10*R*)-11-oxabicyclo[6.2.1]undec-5-ene-2,9,10-triol (2) UDEFT ^{13}C NMR

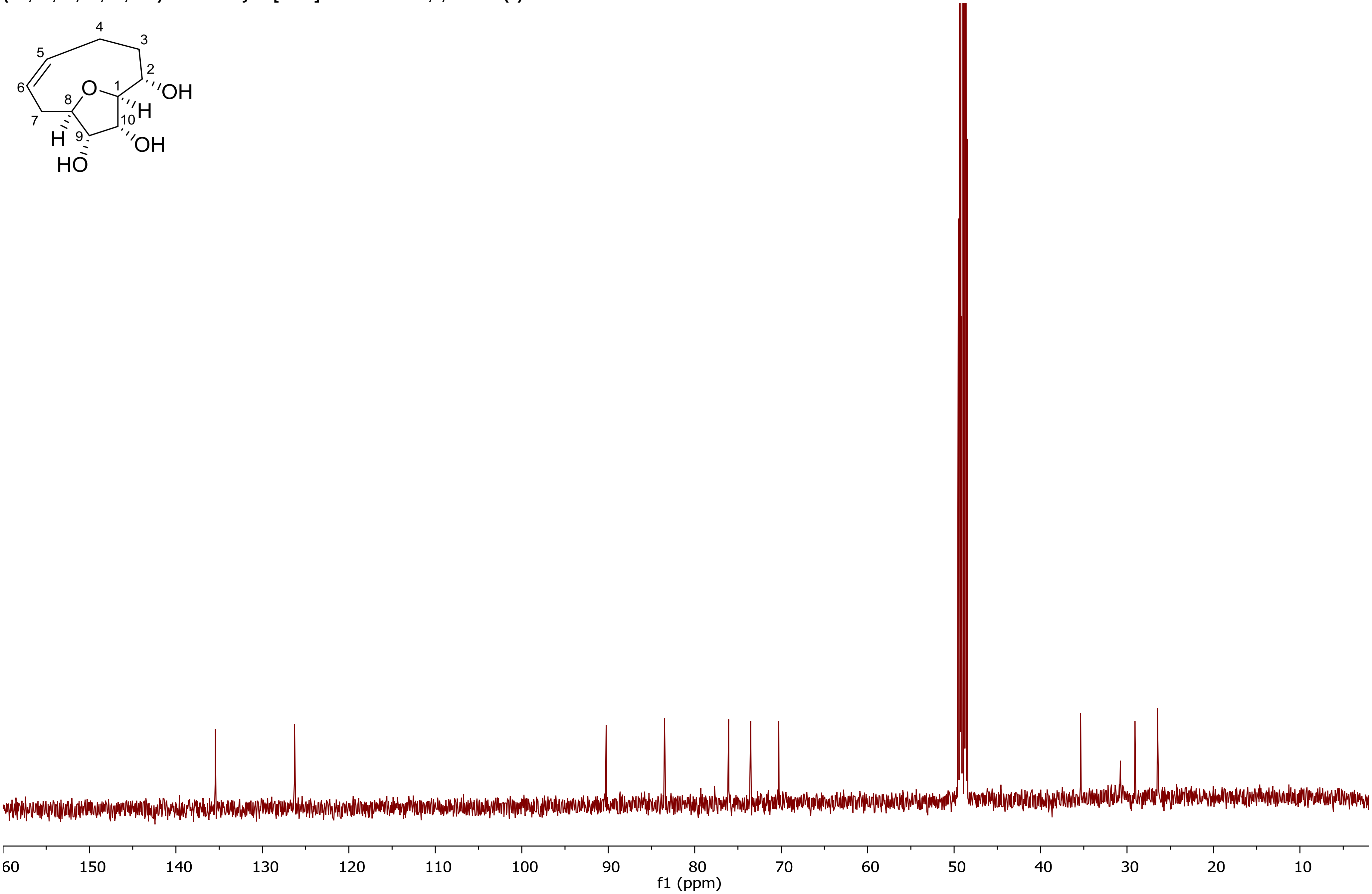

(1*S*,2*S*,5*Z*,8*R*,9*R*,13*S*)-11,11-dimethyl-10,12,14-trioxatricyclo[6.5.1.0^{9,13}]tetradec-5-en-2-ol (11) ^{1}H NMR

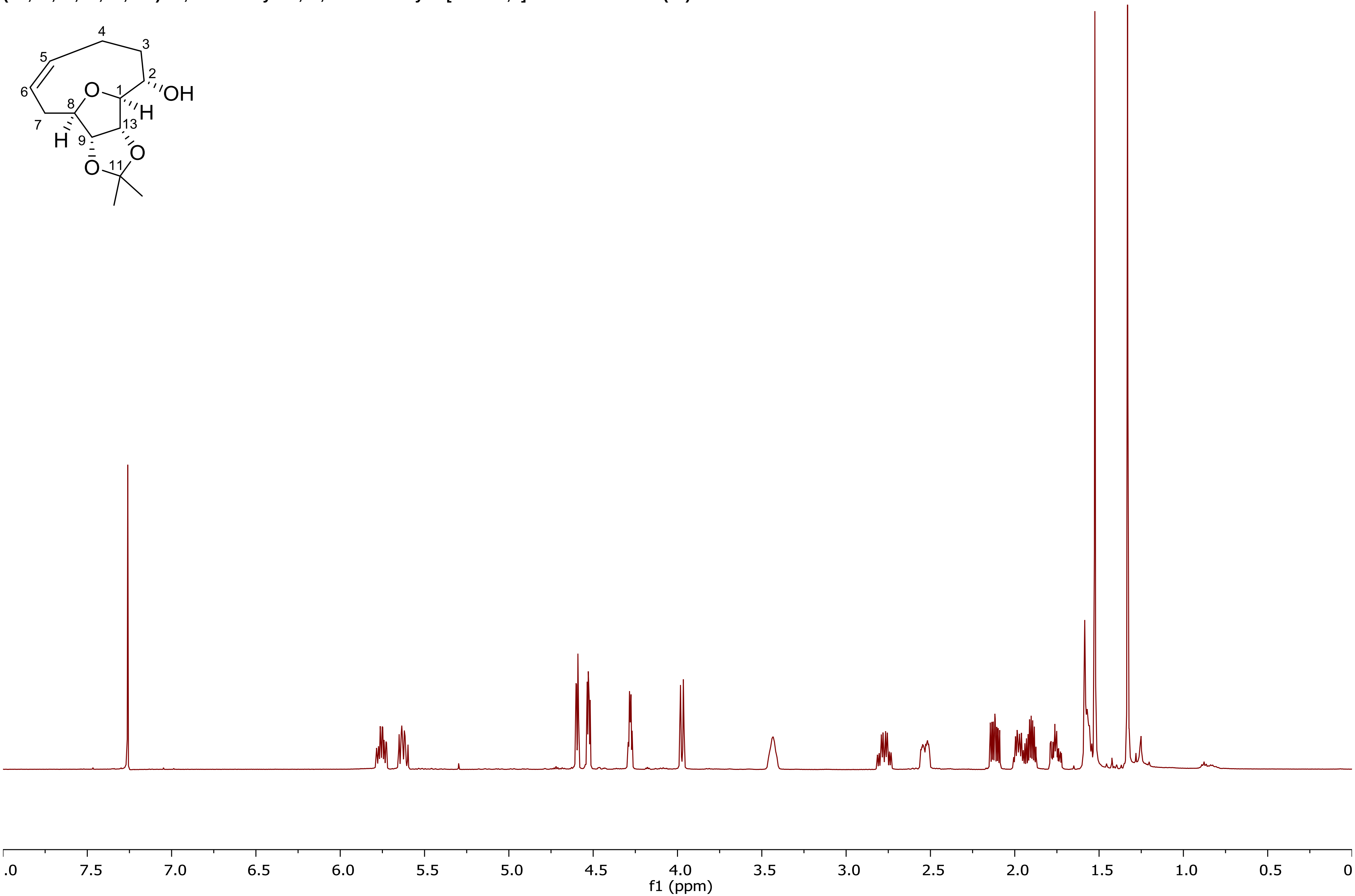

(1*S*,2*S*,5*Z*,8*R*,9*R*,13*S*)-11,11-dimethyl-10,12,14-trioxatricyclo[6.5.1.0^{9},^{13}]tetradec-5-en-2-ol (11) DEPTq ^{13}C NMR

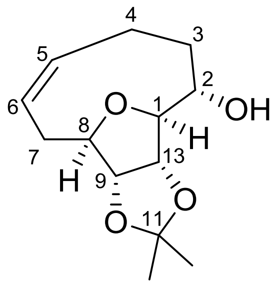

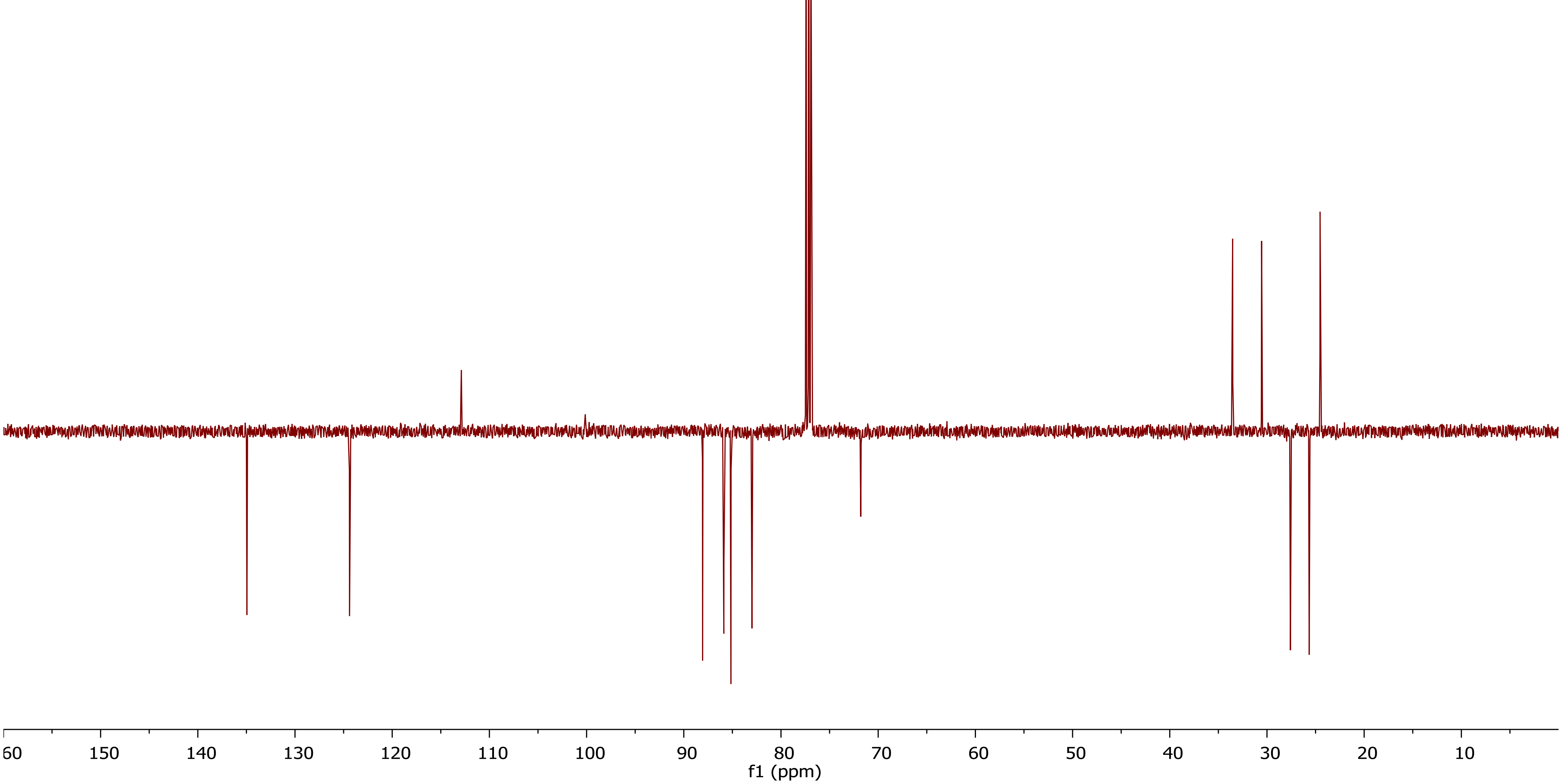

(1*S*,2*S*,8*R*,9*R*,13*S*)-11,11-dimethyl-10,12,14-trioxatricyclo[6.5.1.$0^{9,13}$]tetradecan-2-ol ^{1}H NMR

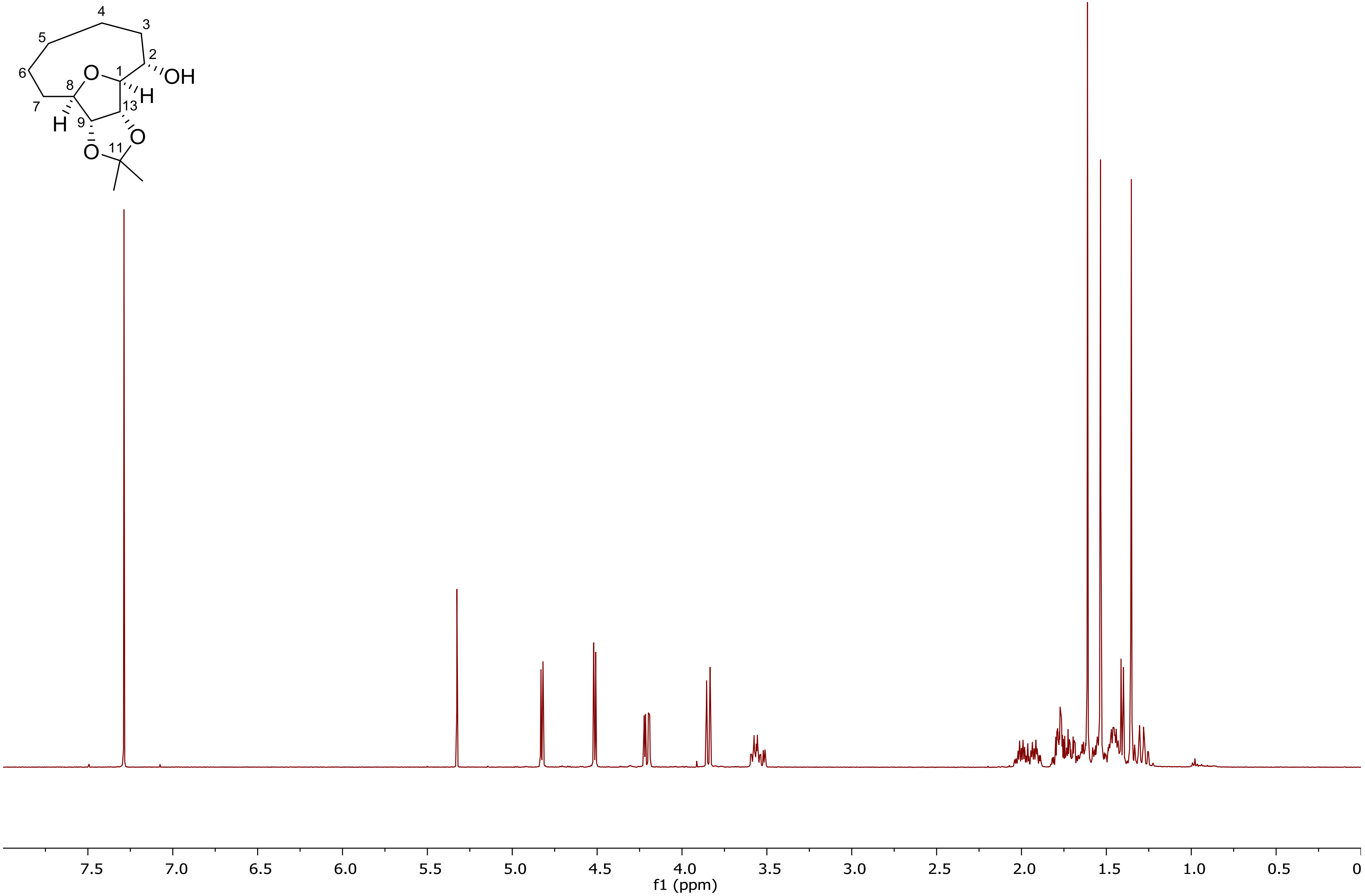

(1*S*,2*S*,8*R*,9*R*,13*S*)-11,11-dimethyl-10,12,14-trioxatricyclo[6.5.1.0⁹,¹³]tetradecan-2-ol UDEFT ^{13}C NMR

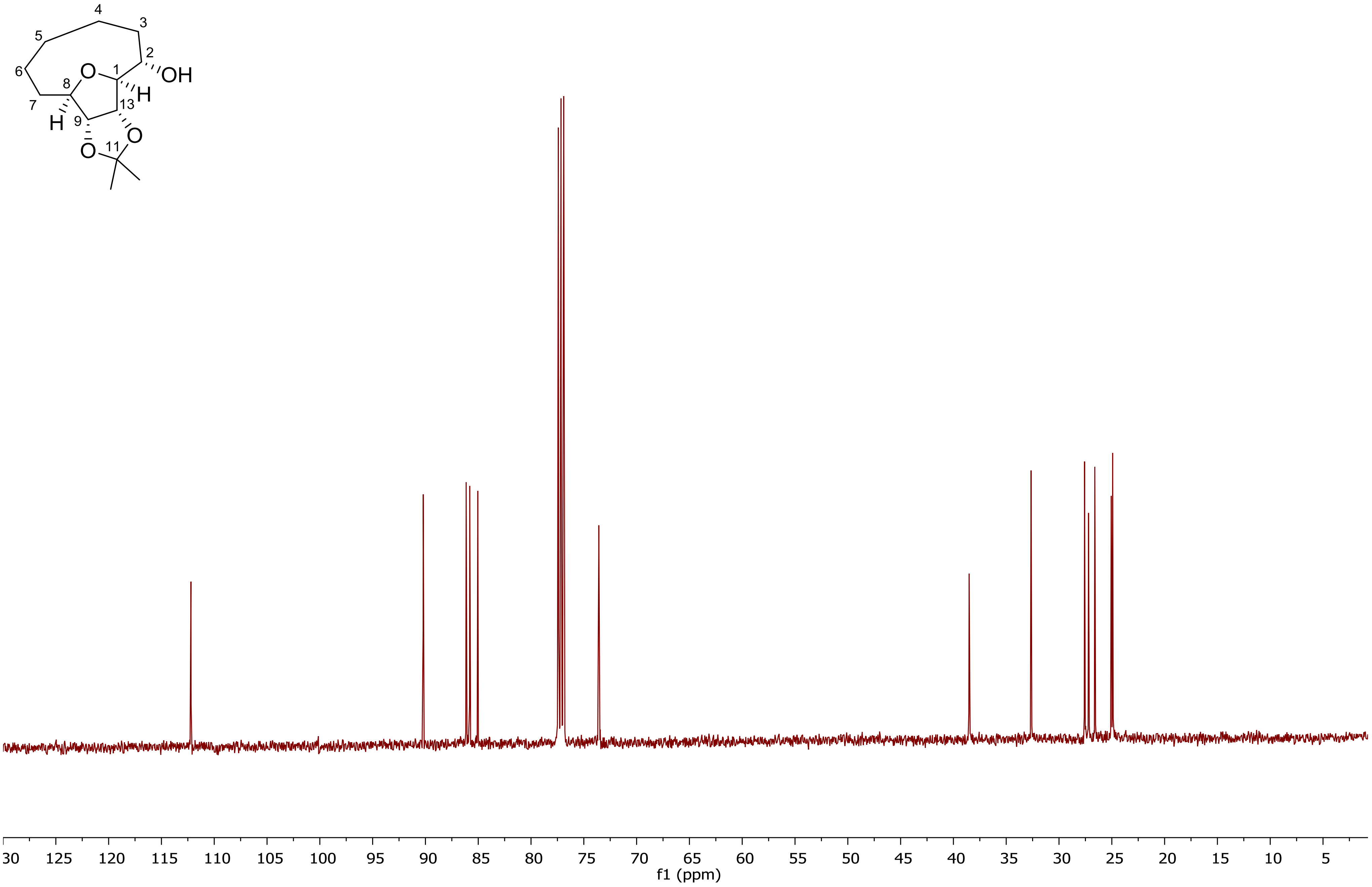

(1*S*,2*S*,8*R*,9*S*,10*R*)-11-oxabicyclo[6.2.1]undecane-2,9,10-triol (12) ^{1}H NMR

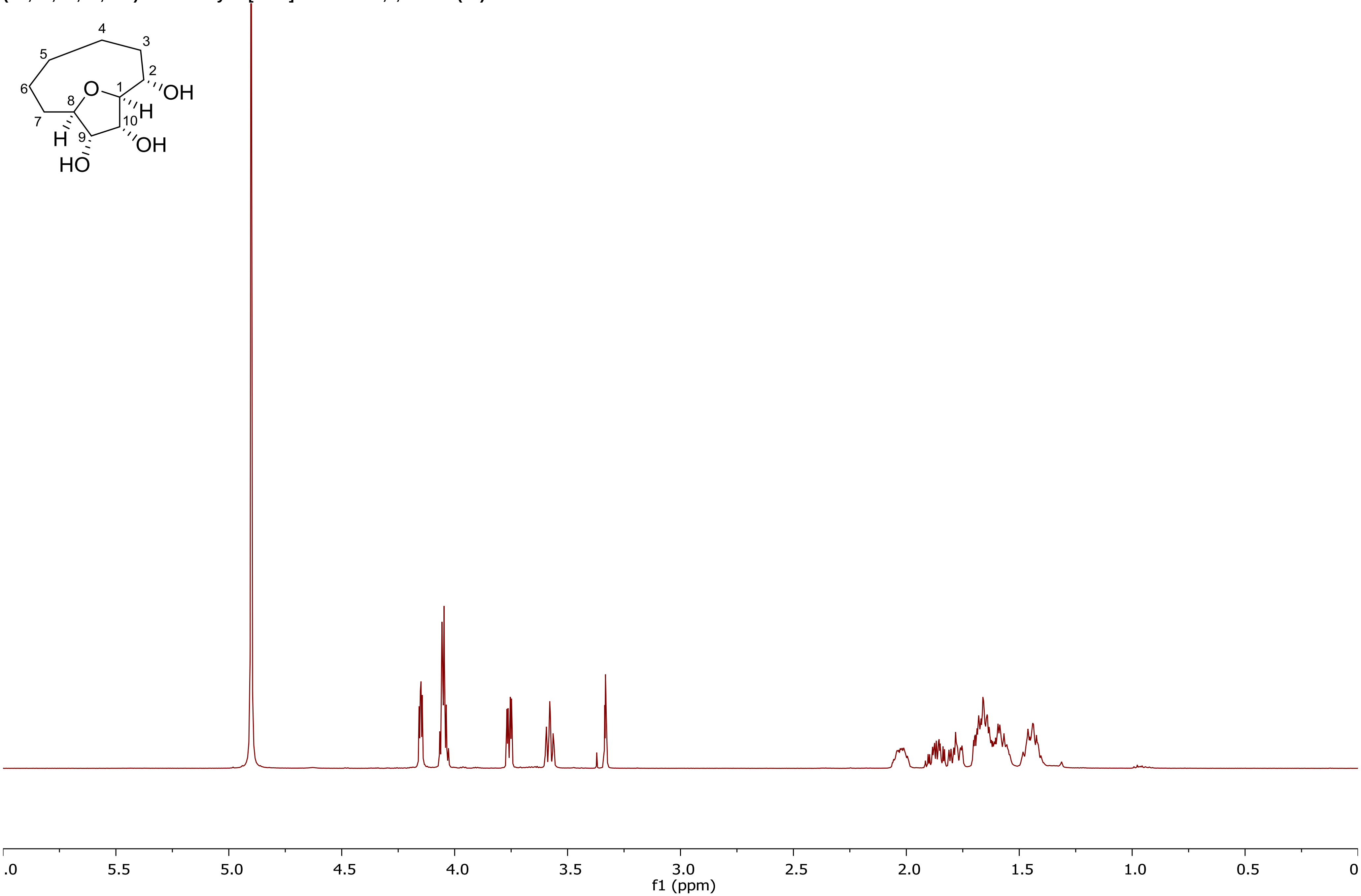

(1*S*,2*S*,8*R*,9*S*,10*R*)-11-oxabicyclo[6.2.1]undecane-2,9,10-triol (12) UDEFT ^{13}C NMR

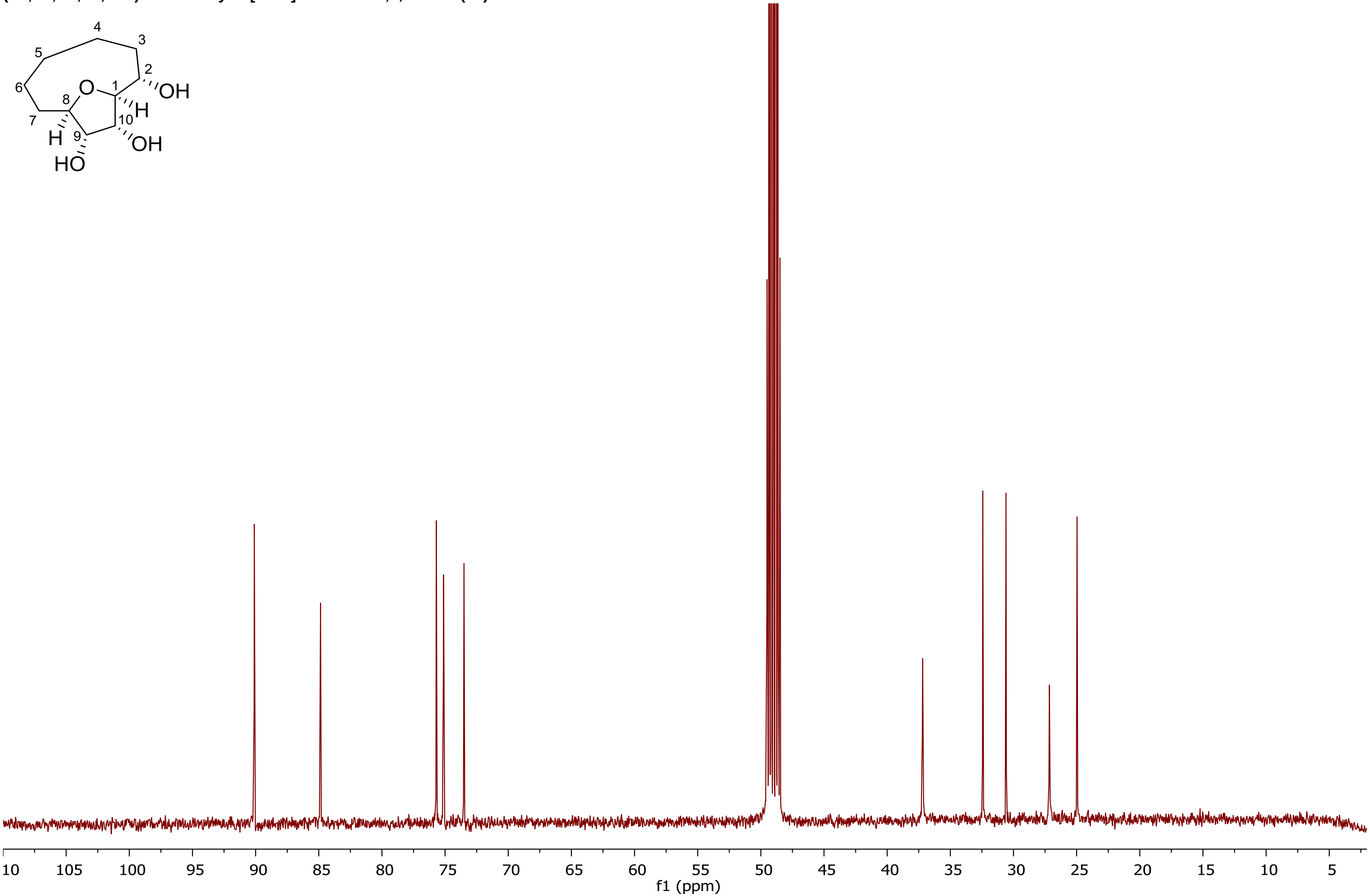

(1*R*,5*Z*,8*R*,9*R*,13*R*)-11,11-dimethyl-10,12,14-trioxatricyclo[6.5.1.0^{9},^{13}]tetradec-5-en-2-one ^{1}H NMR

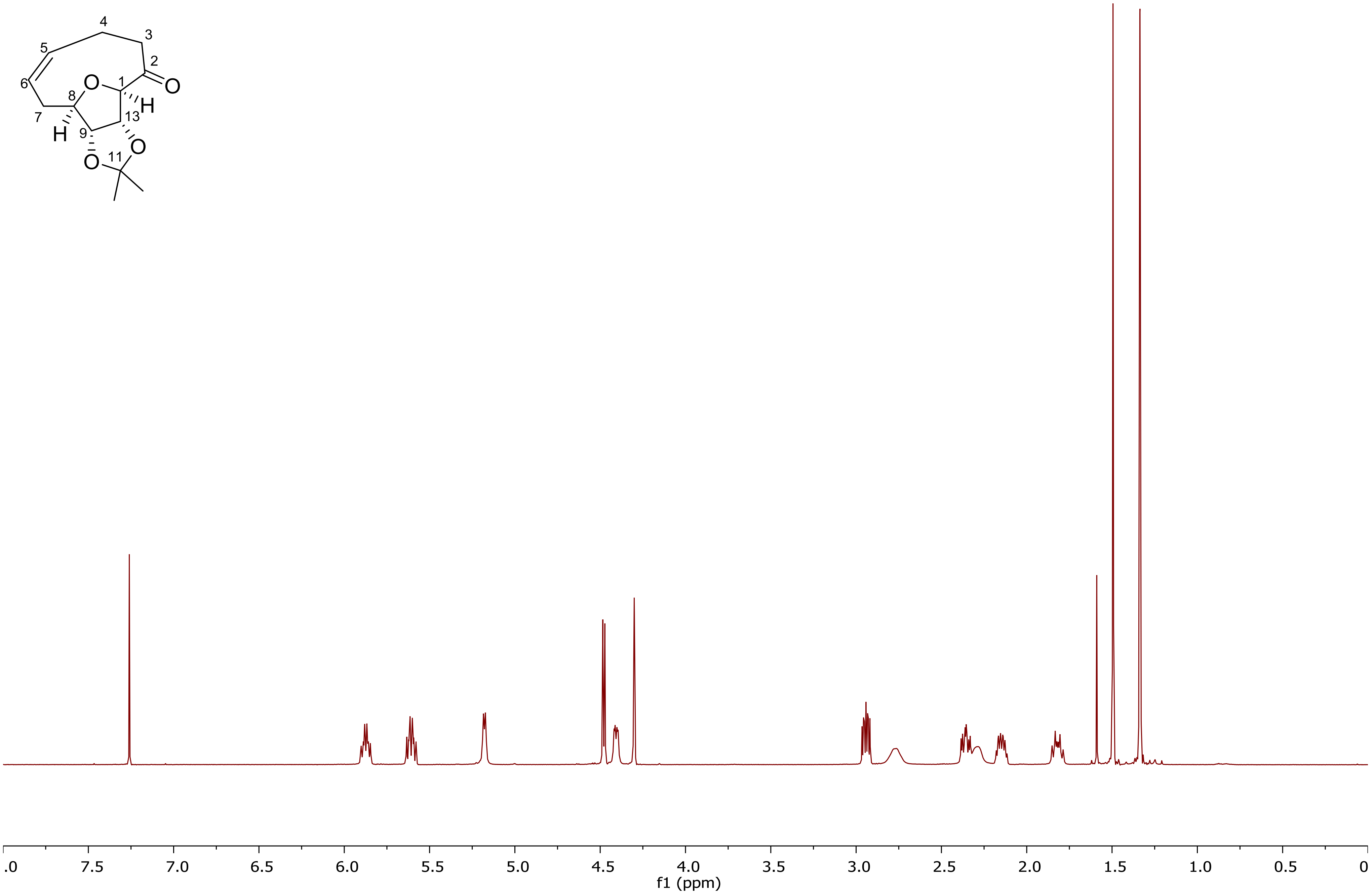

(1*R*,5*Z*,8*R*,9*R*,13*R*)-11,11-dimethyl-10,12,14-trioxatricyclo[6.5.1.0^{9,13}]tetradec-5-en-2-one ^{13}C NMR

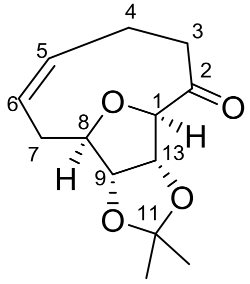

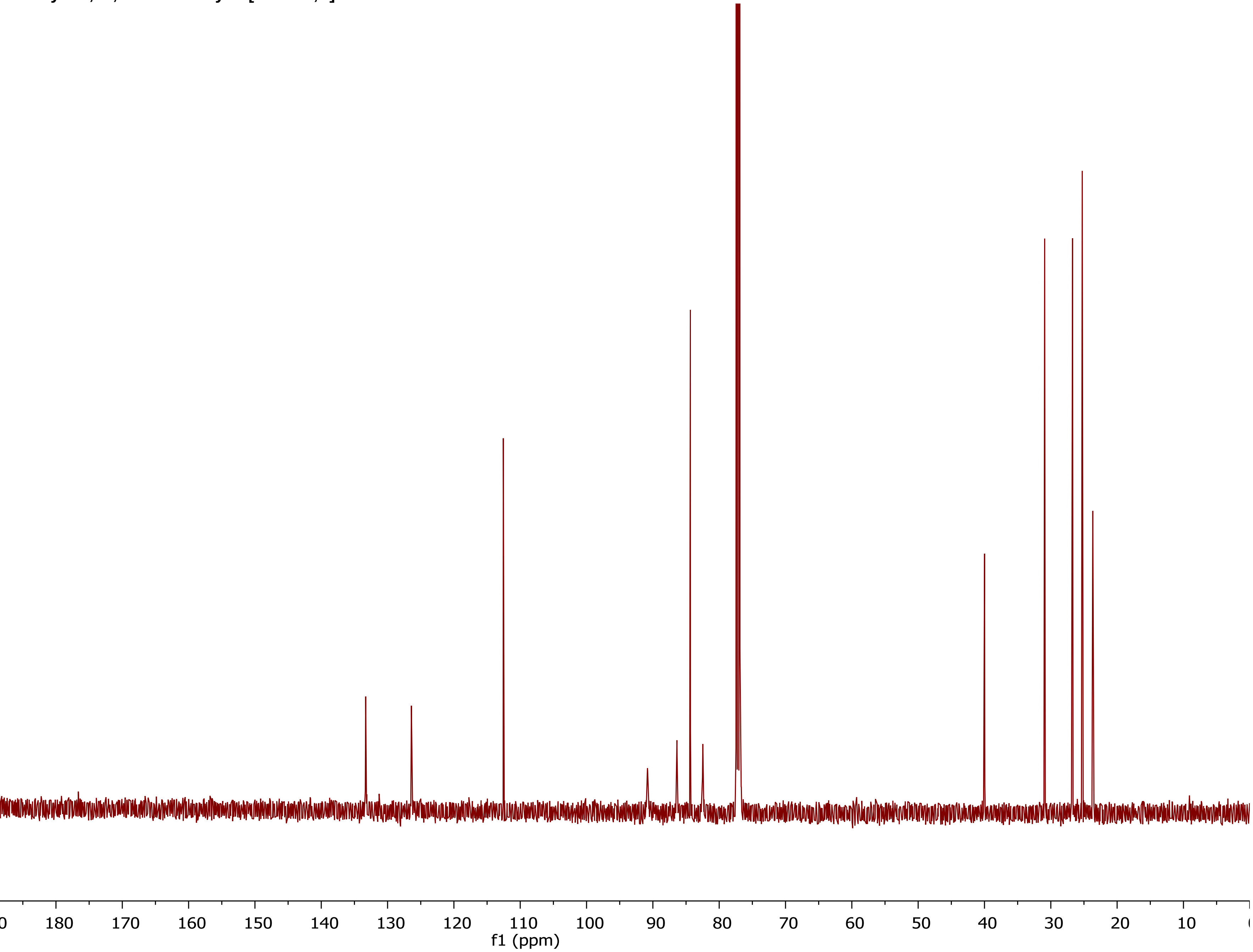

(2*S*)- and (2*R*)-(1*S*,5*Z*,8*R*,9*R*,13*S*)-11,11-dimethyl-10,12,14-trioxatricyclo[6.5.1.0⁹,¹³]tetradec-5-en-2-ol ¹H NMR

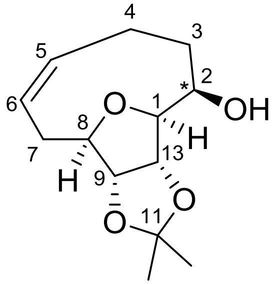

* Major epimer shown

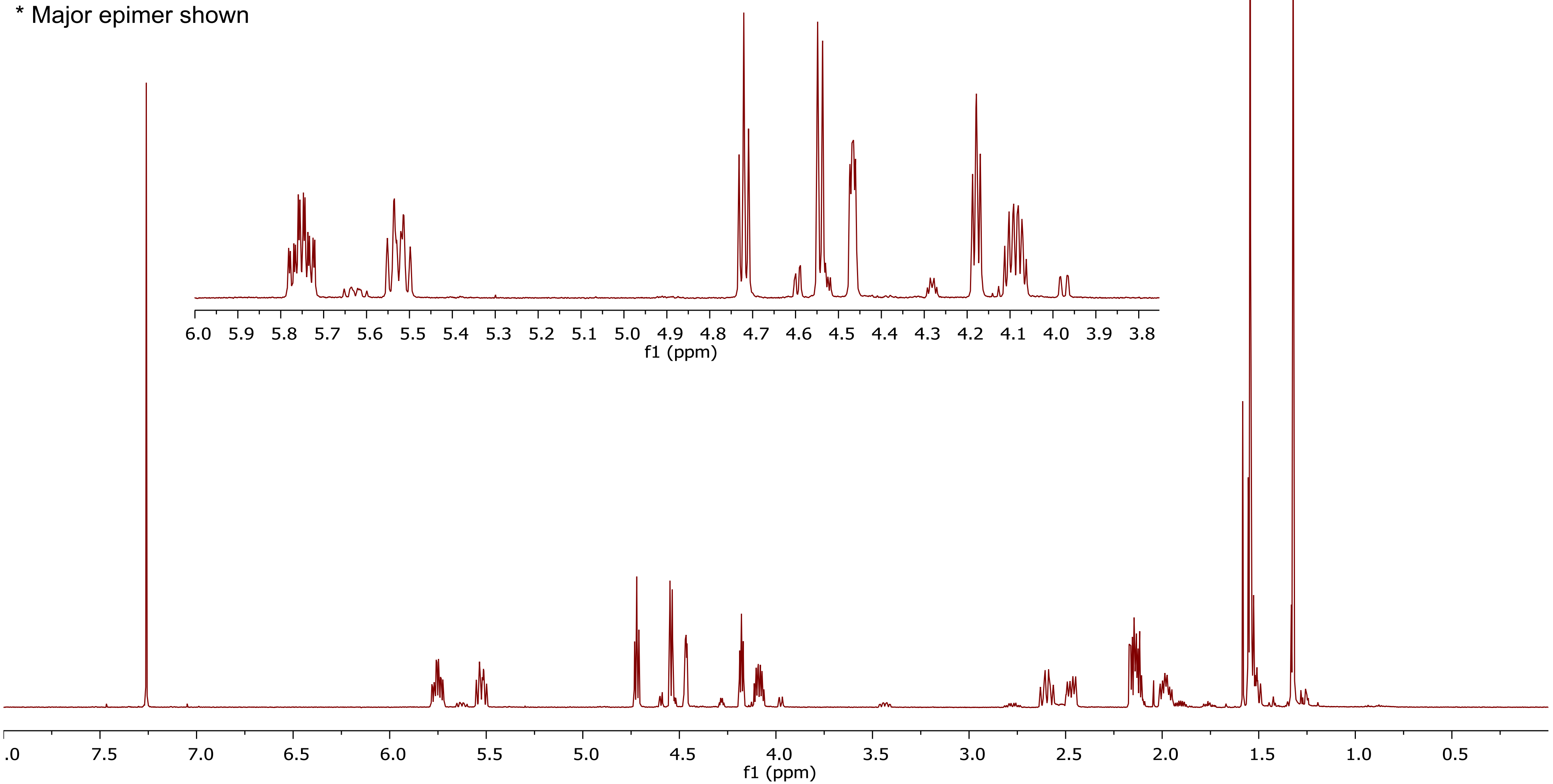

(2*S*)- and (2*R*)-(1*S*,5*Z*,8*R*,9*R*,13*S*)-11,11-dimethyl-10,12,14-trioxatricyclo[6.5.1.0^{9},^{13}]tetradec-5-en-2-ol UDEFT ^{13}C NMR

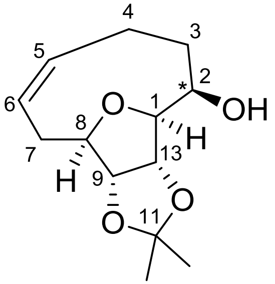

* Major epimer shown

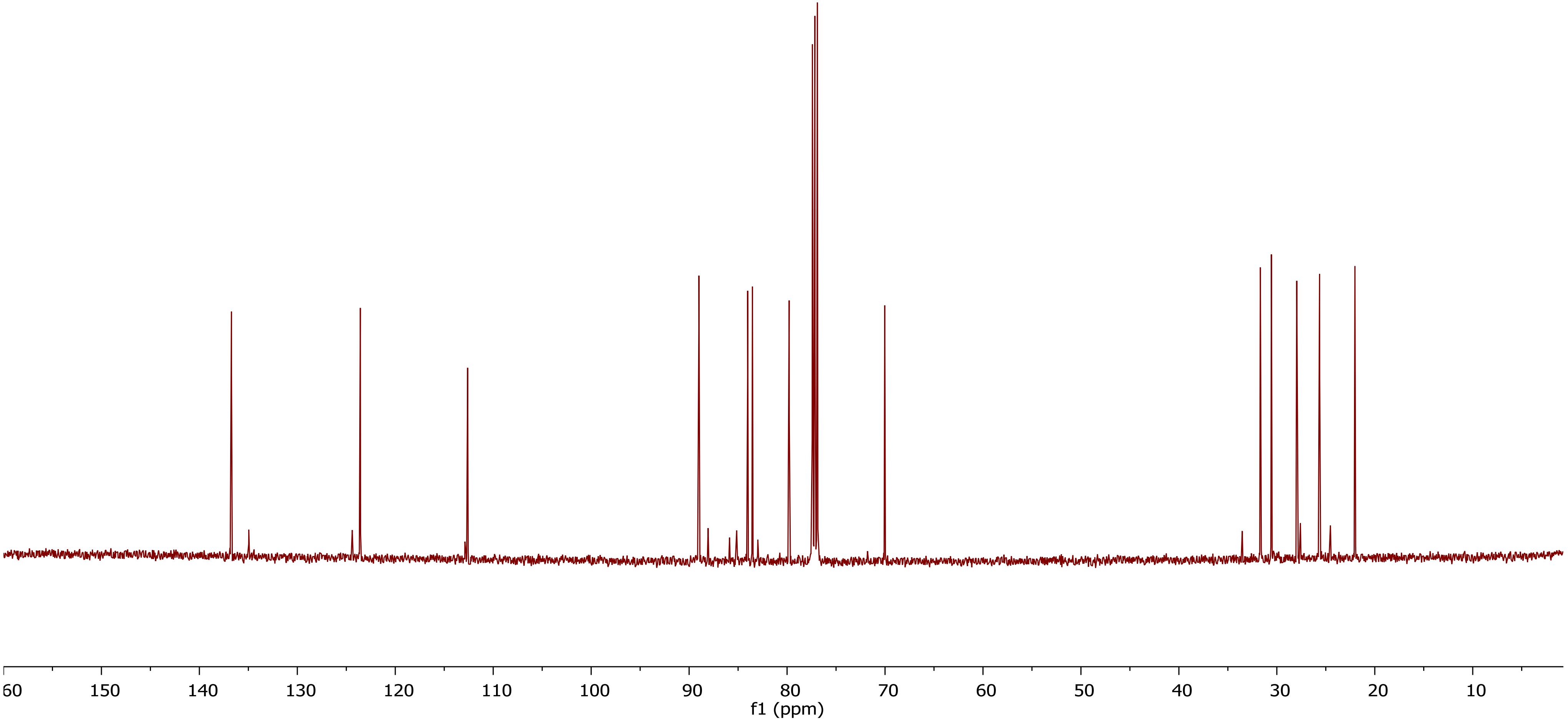

(1*R*,2*R*,5*Z*,8*R*,9*S*,10*R*)-11-oxabicyclo[6.2.1]undec-5-ene-2,9,10-triol (13) ^{1}H NMR

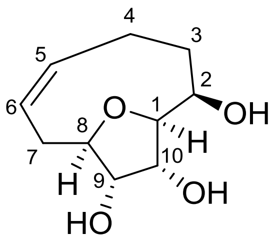

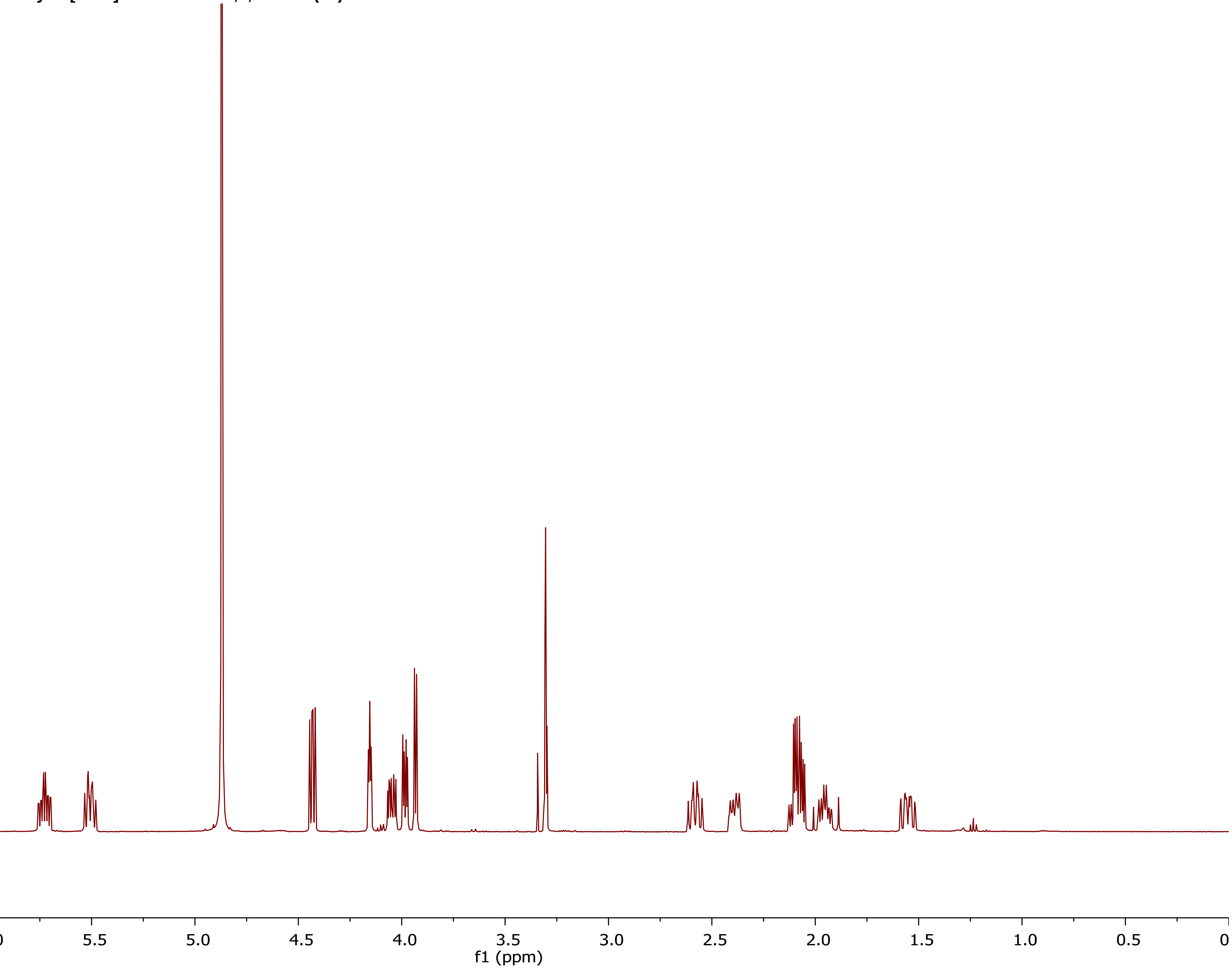

(1*R*,2*R*,5*Z*,8*R*,9*S*,10*R*)-11-oxabicyclo[6.2.1]undec-5-ene-2,9,10-triol (13) UDEFT ^{13}C NMR

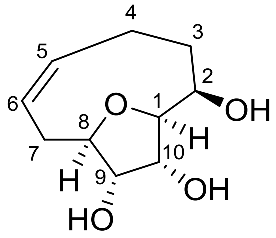

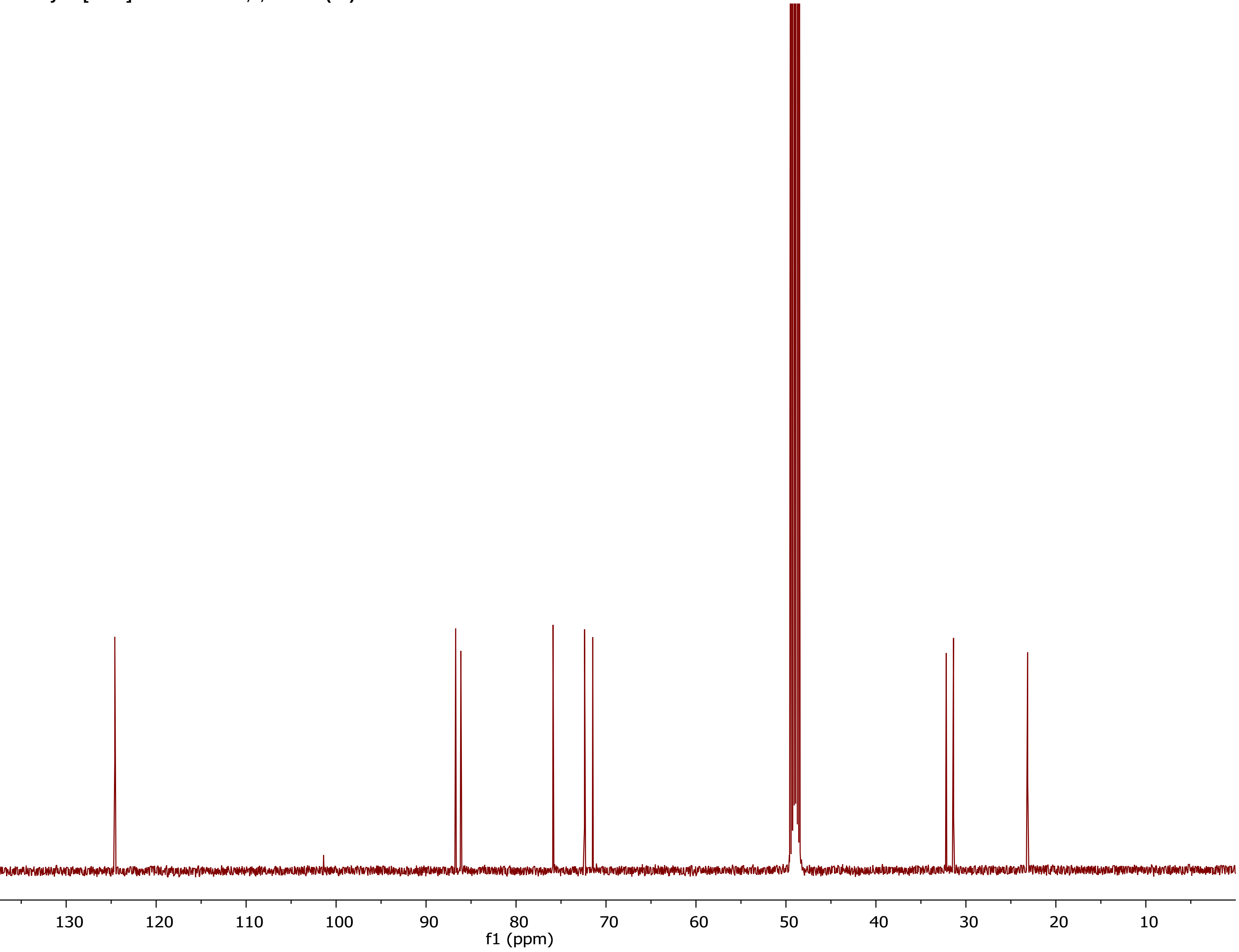

(1*S*,2*S*,5*Z*,8*R*,9*S*,10*R*)-11-oxabicyclo[6.2.1]undec-5-ene-2,9,10-triol (2) NOESY

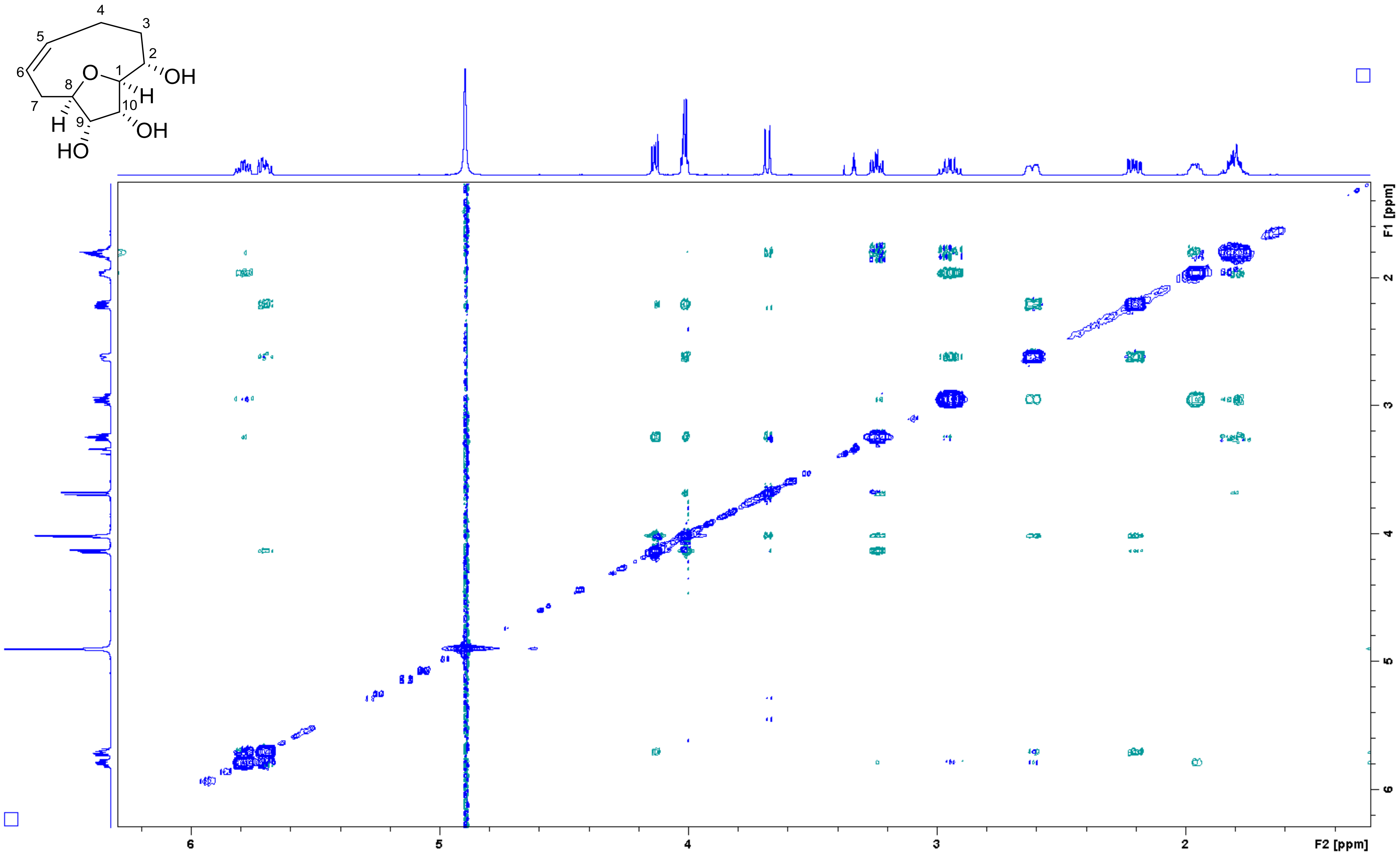

(1*S*,2*S*,8*R*,9*S*,10*R*)-11-oxabicyclo[6.2.1]undecane-2,9,10-triol (12) NOESY

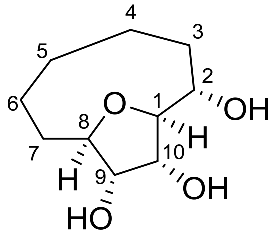

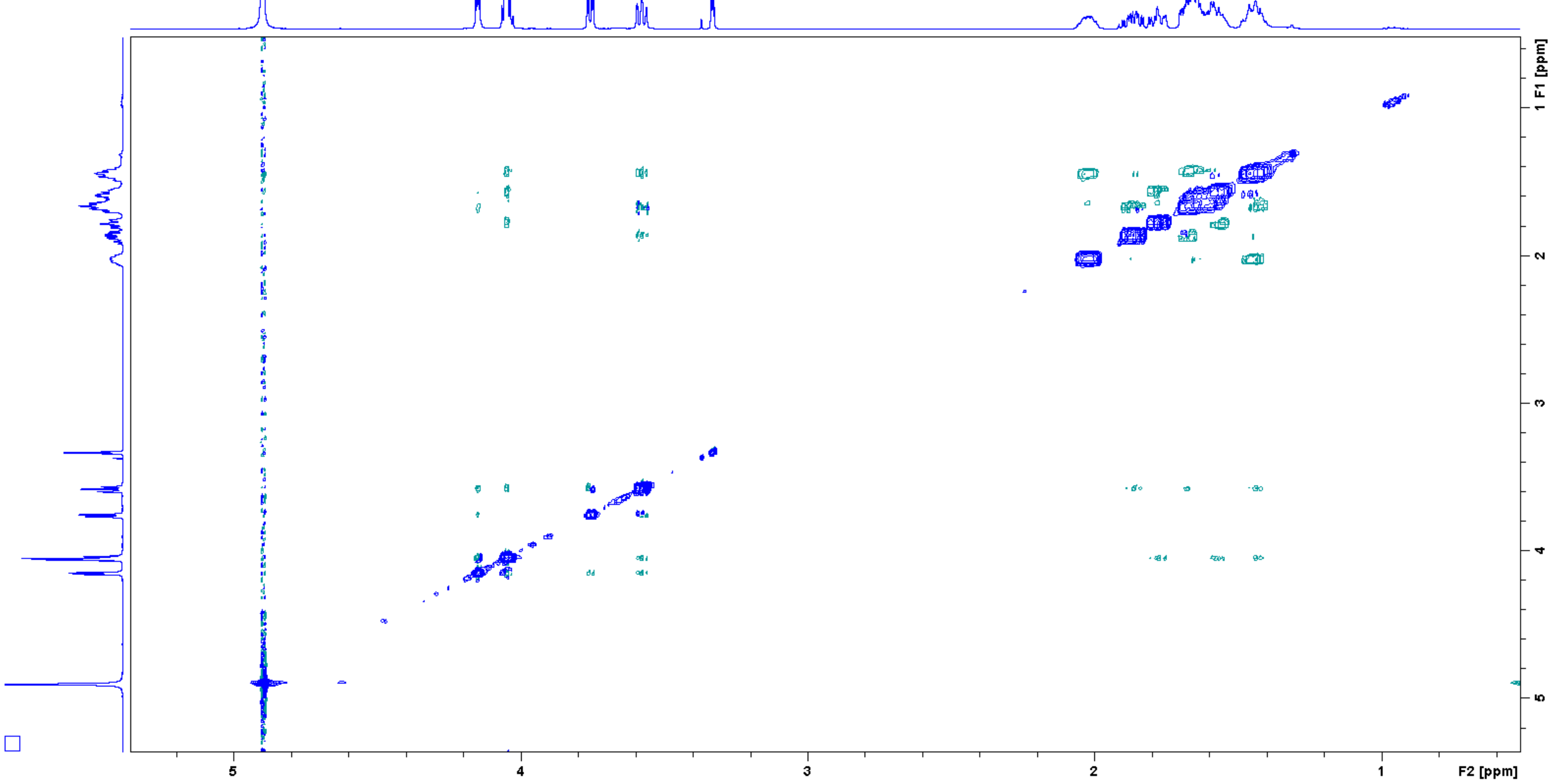

Supplement: Supplementary file 1 — Supporting Information [file EJOC-2016-4496-s001.pdf]
